# A Proteomic Analysis Indicates That Oxidative Stress Is the Common Feature Triggering Antibiotic Production in *Streptomyces coelicolor* and in the *pptA* Mutant of *Streptomyces lividans*

**DOI:** 10.3389/fmicb.2021.813993

**Published:** 2022-03-22

**Authors:** Clara Lejeune, Laila Sago, David Cornu, Virginie Redeker, Marie-Joelle Virolle

**Affiliations:** ^1^Institute for Integrative Biology of the Cell (I2BC), Université Paris-Saclay, CEA, CNRS, Gif-sur-Yvette, France; ^2^Institut Francois Jacob, Molecular Imaging Center (MIRCen), Laboratory of Neurodegenerative Diseases, Commissariat à l’Energie Atomique et aux Energies Alternatives (CEA), Centre National de la Recherche Scientifique, Université Paris-Saclay, Fontenay-aux-Roses, France

**Keywords:** metabolism, respiratory chain, phosphate, oxidative stress, antibiotics, proteomics, label-free protein quantification

## Abstract

In most *Streptomyces* species, antibiotic production is triggered in phosphate limitation and repressed in phosphate proficiency. However, the model strain, *Streptomyces coelicolor*, escapes this general rule and produces actinorhoddin (ACT), a polyketide antibiotic, even more abundantly in phosphate proficiency than in phosphate limitation. ACT was shown to bear “anti-oxidant” properties suggesting that its biosynthesis is triggered by oxidative stress. Interestingly, *Streptomyces lividans*, a strain closely related to *S. coelicolor*, does not produce ACT in any phosphate condition whereas its *pptA/sco4144* mutant produces ACT but only in phosphate limitation. In order to define the potentially common features of the ACT producing strains, these three strains were grown in condition of low and high phosphate availability, and a comparative quantitative analysis of their proteomes was carried out. The abundance of proteins of numerous pathways differed greatly between *S. coelicolor* and the *S. lividans* strains, especially those of central carbon metabolism and respiration. *S. coelicolor* is characterized by the high abundance of the complex I of the respiratory chain thought to generate reactive oxygen/nitrogen species and by a weak glycolytic activity causing a low carbon flux through the Pentose Phosphate Pathway resulting into the low generation of NADPH, a co-factor of thioredoxin reductases necessary to combat oxidative stress. Oxidative stress is thus predicted to be high in *S. coelicolor*. In contrast, the *S. lividans* strains had rather similar proteins abundance for most pathways except for the transhydrogenases SCO7622-23, involved in the conversion of NADPH into NADH. The poor abundance of these enzymes in the *pptA* mutant suggested a deficit in NADPH. Indeed, PptA is an accessory protein forcing polyphosphate into a conformation allowing their efficient use by various enzymes taking polyphosphate as a donor of phosphate and energy, including the ATP/Polyphosphate-dependent NAD kinase SCO1781. In phosphate limitation, this enzyme would mainly use polyphosphate to phosphorylate NAD into NADP, but this phosphorylation would be inefficient in the *pptA* mutant resulting in low NADP(H) levels and thus high oxidative stress. Altogether, our results indicated that high oxidative stress is the common feature triggering ACT biosynthesis in *S. coelicolor* and in the *pptA* mutant of *S. lividans*.

## Introduction

In all living organisms, phosphorus is a major constituent of essential biological macromolecules such as DNA, RNA, and membranous phospholipids (PLs). It also plays a key role in energy metabolism *via* oxidative phosphorylation and ATP synthesis. ATP itself fulfills many regulatory roles acting as an allosteric effector of some enzymes and regulators and as a trigger factor of proteins phosphorylation cascades. Since phosphate is often limited in the environment, the consequences of a limitation in phosphate (Pi) has been extensively studied in microorganisms and plants (Abel et al., 2002; Gupta and Laxman, 2021). This research field was especially active in *Streptomyces* species since the production of antibiotics by these Gram-positive filamentous soil bacteria is usually triggered in a condition of Pi limitation and strongly repressed in Pi proficiency (Martin, 2004; Martin and Liras, 2021). However, the extensively studied model strain, *Streptomyces coelicolor* (*SC*), escapes this general rule since it produces the blue pigmented polyketide antibiotic, actinorhoddin (ACT), in both phosphate conditions and even more abundantly in phosphate proficiency than in phosphate limitation (Esnault et al., 2017). In contrast, *Streptomyces lividans* (*SL*), a strain phylogenetically closely related to *SC*, which has the genetic capability to produce ACT, does not produce it in any Pi condition, whereas the *pptA* mutant derived from it produces ACT in phosphate limitation but not in phosphate proficiency (Shikura et al., 2021), as most *Streptomyces* species. The *pptA* mutant is an in-frame deletion of *sco4144* that encodes a phosin-like protein possessing a polyphosphate (polyP) binding module (CHAD domain) (Werten et al., 2019). PptA, which is mainly expressed in conditions of phosphate limitation and belongs to the Pho regulon (Shikura et al., 2021), was proposed to act as an accessory factor forcing polyP into a conformation allowing their efficient use by various enzymes using polyP as donor of phosphate and/or energy (Werten et al., 2019). These potentially include exo-polyphosphatases or general phosphatases involved in the degradation of polyP into phosphate (Shikura et al., 2021) or the polyphosphate kinase Ppk/SCO4145 that uses polyP to regenerate ATP from ADP (Chouayekh and Virolle, 2002; Ghorbel et al., 2006b). The *pptA* mutant thus suffers phosphate/energetic stress, and a strong induction of the expression of the two-component system (TCS) PhoR/PhoP that controls positively the expression of genes of the Pho regulon involved in phosphate scavenging and uptake was observed in this strain (Shikura et al., 2021). In contrast, the expression of PhoR/PhoP and proteins under its positive control was shown to be lower in *SC* than in *SL* in Pi limitation (Millan-Oropeza et al., 2020), and *SC* has a 2–3-fold higher intracellular ATP content than *SL* (Esnault et al., 2017). Furthermore, a mutant strain of *SC* deleted for the ACT cluster was shown to be far more sensitive to the oxidant diamide than the original strain (Millan-Oropeza et al., 2020). This demonstrated that ACT has an “anti-oxidant” function and suggested that its biosynthesis might be triggered by oxidative stress. In order to define the potentially common features responsible for the generation of high oxidative stress in the two ACT-producing strains, *SC* and the *pptA* mutant of *SL*, these strains as well as the *wt* strain of *SL* were grown in the classical R2YE medium limited (1 mM) or proficient (5 mM) in inorganic phosphate (Pi), and a comparative quantitative analysis of their proteomes that nicely covered most metabolic pathways was carried out. In *SL*, taken as the reference strain, proteins of each ontological class could be classified into three groups: group I, proteins whose abundance does not vary with Pi availability; group II, proteins upregulated in Pi proficiency (or downregulated in Pi limitation); and group III, proteins upregulated in Pi limitation (or downregulated in Pi proficiency). Groups II and III should include proteins under the, direct or indirect, negative and positive control, respectively, of the TCS PhoR/PhoP at least in *SL* (Allenby et al., 2012).

Our analysis revealed that proteins of most ontological classes of the two *SL* strains had a similar abundance and responded in a similar, but not identical, way to phosphate availability and thus belonged to the same groups. The slight differences in the response to Pi availability between the two *SL* strains are thought to be due to the previously reported upregulation of proteins of the Pho regulon involved in Pi scavenging and transport, in the *pptA* mutant in a condition of Pi limitation (Shikura et al., 2021). However, interestingly, the main difference between the *wt* strain of *SL* and its *pptA* mutant concerned enzymes involved in nicotinamide adenine dinucleotide phosphate (reduced form) [NADP(H)] metabolism. In contrast, the abundance and response to Pi availability of numerous proteins of *SC* differed drastically from that of the *SL* strains, especially that of the enzymes belonging to central carbon metabolism and to the respiratory chain. The proteins of these pathways thus belonged to different groups in the *SL* strains and in *SC*. The consequences of their different abundance patterns on the metabolism of the studied strains and more specifically on the generation of high oxidative stress are reported, and the possible cause of the utterly different metabolic features of these two closely related strains is discussed.

## Materials and Methods

### Bacterial Strains, Media, and Culture Conditions

Spores of *SC* M145 (Bentley et al., 2002), *SL* TK24 (Ruckert et al., 2015), and the *pptA* mutant derived from *SL* (Shikura et al., 2021) were prepared from solid soya flour medium (SFM) (Hopwood et al., 1985; Kieser et al., 2000). The three strains were grown, in quadruplets, on solid modified R2YE medium, with no sucrose added, in 5 cm diameter Petri dishes. This medium was supplemented with glucose (50 mM) as major carbon source and was either limited (1 mM, no K_2_HPO_4_ added) or proficient (4 mM, K_2_HPO_4_ added) in Pi. About 10^6^ spores of the strains were plated on the surface of cellophane disks (Focus Packaging and Design Ltd., Louth, United Kingdom) laid down on the top of agar plates and incubated at 28°C in darkness for 48 or 60 h. Cell growth was assessed by dry cell weight, every 12 h, from 24 h to 96 h of cultivation. To do so, mycelial lawns of the four independent biological replicates of each strain were collected with a spatula at each culture time, washed twice with deionized water, lyophilized, and weighted.

An in-depth shotgun label-free comparative analysis was carried out from four biological replicates of each strain grown for 48 and 60 h on solid modified R2YE medium either limited (1 mM) or proficient (5 mM) in Pi, for 48 and 60 h, as described in Millan-Oropeza et al. (2020). The time points of 48 and 60 h were chosen as they correspond to the beginning of the production of the blue pigmented polyketide antibiotic, ACT, in the two antibiotic-producing strains (Shikura et al., 2021).

### Total Proteins Extraction and Digestion

A total number of 48 samples (3 strains × 2 media × 2 culture times × 4 biological replicates) were thus subjected to a deep shotgun label-free comparative analysis. The samples were alkylated before digestion by lysyl endopeptidase (Wako) and sequencing-grade modified trypsin (Promega). The resulting proteolytic peptides were pre-cleaned, concentrated under vacuum, and stored before mass spectrometry analysis as described previously (Millan-Oropeza et al., 2020).

### Liquid Chromatography Tandem Mass Spectrometry Analysis

Proteolytic peptides were analyzed by nanoLC-MS/MS (liquid chromatography tandem mass spectrometry) using a nanoElute liquid chromatography system (Bruker) coupled to a timsTOF Pro mass spectrometer (Bruker). Protein digests (1 μg in 2 μl of 2% acetonitrile and 0.05% trifluoroacetic acid in water loading buffer) were loaded on an Aurora analytical column (ION OPTIK, 25 cm × 75 μm, C18, 1.6 μm) and eluted with a gradient of 0–35% of solvent B for 100 min. Solvent A was 0.1% formic acid and 2% acetonitrile in water, and solvent B was 0.1% formic acid and 99.9% acetonitrile. MS and MS/MS spectra were recorded, and raw data were processed and converted into mgf files as described previously (Shikura et al., 2021).

### Protein Identifications

Protein identifications were performed against *SC* and *SL* protein database from UniprotKB (15012020) using the MASCOT search engine (Matrix science, London, United Kingdom). Database searches were performed using the following parameters: specific trypsin digestion with two possible miscleavages; carbamidomethylation of cysteines as fixed modification and oxidation of methionines as variable modification. Peptide and fragment tolerances were 25 ppm and 0.05 Da, respectively. Protein identifications were validated when identified with at least two unique peptides in at least one replicate, identified with a score higher than the identity threshold, and a false-positive discovery rate of less than 1% (Mascot decoy option).

### Label-Free MS-Based Relative Protein Quantification

Protein abundance changes were determined using two label-free mass spectrometry-based quantification methods: spectral count (SC) or MS1 ion intensities named XIC (for extracted ion current). For spectral counting, total MS/MS SC values were extracted from Scaffold software (version Scaffold_4.11.1, Proteome software Inc., Portland, OR, United States) filtered with 95% probability and 1% false discover rate (FDR) for protein and peptide thresholds, respectively. For MS1 ion intensity, MS raw files were analyzed with Maxquant software (v 1.6.10.43) using the maxLFQ algorithm with default settings and 4D feature alignment corresponding to a match between run function including collisional cross sections (CCS) alignment. Normalization was set as default. Identifications with Andromeda were performed using the same search parameters as those described previously for MASCOT searches.

### Protein Abundance Changes and Statistical Analysis

Statistical quantitative analyses were based on two different generalized linear models depending on the type of quantification method used and data that were generated: either spectral counting for a rough relative quantitative protein quantification or XIC from MS1 ion intensities for more accurate and sensitive relative quantifications of low abundant proteins or small abundant changes. The discrete SC (1) and continuous XIC (2) abundances values were modeled, respectively, as follows and as described previously (Shikura et al., 2021):

1. SC = μ + strain + medium + time + replicate + strain* medium + strain*time + medium*time + strain*medium* time + ε∼ Pois(λ)
2. Log_2_(LFQ) = μ + strain + medium + time + replicate + strain*medium + strain*time + medium*time + strain* medium*time + ε∼ N(0,σ).

Terms represent fixed effect of the different conditions and their interactions for each protein abundance. The residual error (ε) follows a Poisson distribution [Pois(λ)] or a normal distribution for SC and Log_2_ (LFQ), respectively. Effects were estimated by maximum likelihood. Statistical significances were calculated using likelihood ratio tests based on the analysis of deviance. *p*-values were adjusted using the Benjamini–Hochberg procedure for multiple testing correction. For each protein, first, a significant difference in abundance for the 66 combinations of pairwise comparisons was set at 10 for SC and 1 Log_2_ fold change for LFQ values. Second, a threshold of 10 significant pairs (minimum number of significant pairwise comparisons expected for a differential protein abundance in at least two conditions) and an adjusted *p*-value of 0.05 was used to consider a protein abundance as significantly variable. These statistical analyses were performed in R Studio (versión 1.4.17.17) using a homemade R script using the described models and parameters.

### Data Set Analysis and Deposit

Descriptive analysis of the protein abundances was performed using heatmap representations in R 3.3.2. Heatmaps were constructed using hierarchical clustering based on Euclidean distances. For proteins that were quantified with both methods (XIC and SCs), only the values obtained by XIC-based approach were used in the heatmap constructions since the latter is a finer quantitative approach compared to the SCs-based method.

Our mass spectrometry proteomics data have been deposited to the ProteomeXchange (Deutsch et al., 2020). Consortium *via* the PRIDE (Perez-Riverol et al., 2019) partner repository with the dataset identifier PXD029263 and 10.6019/PXD029263.

## Results

### Bacterial Growth and Conditions of Proteome Analysis

**FIGURE 1:**
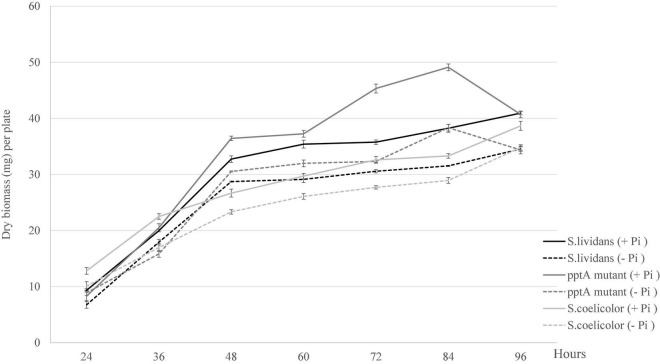
Growth curves of the strains, *S. lividans* TK24 (black lines), mutant *pptA* derived from *S. lividans* TK24 (dark gray lines), and *S. coelicolor* (light gray lines) grown on solid modified R2YE medium proficient (5 mM, continuous lines) or limited (1 mM, dotted lines) in phosphate (1 mM).

In order to determine the impact of Pi availability on growth of the strains under study, growth was estimated by dry biomass weight per plate. As anticipated, the growth of the three strains was slightly more active in Pi proficiency than in Pi limitation (Figure 1). Unexpectedly, an important increase in the biomass yield of the *pptA* mutant was observed between 60 and 84 h of cultivation, in a condition of Pi proficiency. Previous work carried out with *SL* indicated that at these late time points, the Pi of the growth medium is exhausted (Smirnov et al., 2015). This exhaustion would trigger the expression of genes of the Pho regulon providing sufficient Pi to promote temporary growth. This phase of active growth was followed by an abrupt decrease of the biomass between 84 and 96 h suggesting cell lysis.

### Proteome Analysis

In order to determine the impact of Pi availability on primary and specialized metabolism, total protein extracts of 48 samples resulting from four biological replicates of *SL* WT (Ruckert et al., 2015), its *pptA/sco4144* (Shikura et al., 2021) deletion mutant, and *SC* M145 (Bentley et al., 2002) grown on R2YE limited (1 mM) and proficient (5 mM) in Pi for 48 and 60 h were subjected to an in-depth shotgun label-free comparative analysis as described in section “Materials and Methods” and in Millan-Oropeza et al. (2020). These two time points were chosen as they correspond to the beginning of the production of the blue pigmented polyketide antibiotic, ACT, in the two antibiotic-producing strains (Shikura et al., 2021). Proteomes of the different strains grown in Pi proficiency or limitation were compared using label-free mass spectrometric-based relative quantification with either spectral counting or MS1 ion intensities named XIC. Effects of the different strains and growth conditions were analyzed using an optimized statistical quantitative analyses described in section “Materials and Methods.”

Temporal proteomic profiles were constructed using the relative protein abundances obtained from XIC and SC approaches of proteins showing significant abundance variation; these values were scaled using self-organizing tree algorithm (SOTA) clustering. Data analysis was conducted in R 3.3.2 (R Core Team, 2013), using the package “made4” (Culhane et al., 2005). Protein abundance was represented as heatmaps that were constructed using hierarchical clustering based on Euclidean distances. For proteins that were quantified with both methods (XIC and SCs), only the values obtained with the XIC-based approach were used in the heatmap constructions since the latter is a finer quantitative approach than the SC-based method. In most heatmaps the quantification methods are displayed in vertical bars indicating proteins quantified by spectral counting (orange) or XIC (black). However, when these bars are absent, this indicates that all proteins were quantified by XIC. The larger set of proteins even published in *Streptomyces* (over 4,000 proteins) was obtained. Proteins were classified into ontological classes to facilitate analysis, and protein identifiers are indicated as SCO numbers that correspond to genes of *S. coelicolor* and by predicted functions. We choose to show in the body of the paper only the proteomes of the pathways that are relevant to our scientific question. All the other data that are likely to be of great interest for the *Streptomyces* scientific community, since they reveal how protein abundance varies with Pi availability, will be provided as heatmaps in Supplementary Data.

### In-Depth Shotgun Label-Free Comparative Proteomic Analysis

#### Central Carbon Metabolism

##### Glycolysis

**FIGURE 2:**
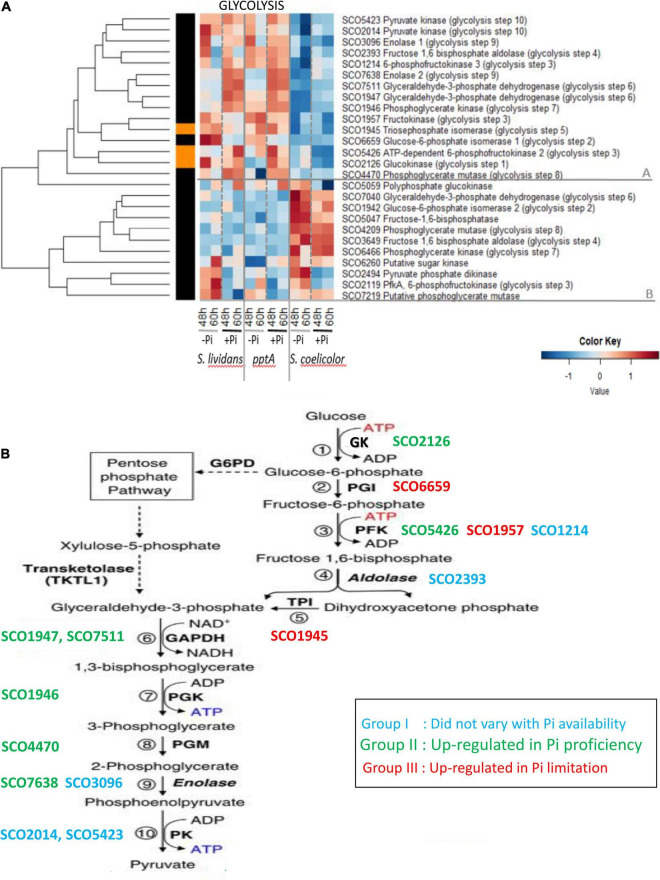
**(A)** Heatmap representation of glycolytic/gluconeogenic enzymes. **(B)** Schematic representation of glycolysis and different abundance features of glycolytic enzymes, identified by their *S. coelicolor* names (SCO), according to phosphate availability. Group I (blue): enzymes whose abundance did not vary with Pi availability; Group II (green): enzymes upregulated in Pi proficiency; Group III (red): enzymes upregulated in Pi limitation.

The heatmap of Figure 2 clearly indicates that the expression pattern of glycolytic enzymes could be divided into two main clusters, A and B, that have contrasted abundance features between the *SC* and *SL* strains.

Cluster A includes 15 proteins that were far less abundant in *SC* than in the *SL* strains. In *SC*, the abundance of proteins of cluster A did not vary with Pi availability, whereas in the *SL* strains, these proteins were distributed among the three previously defined groups as summarized in Figure 2B. Proteins of group I whose abundance did not vary with Pi availability included the phosphofructokinase SCO1214 (PFK, step 3), the fructose 1, 6 bisphosphate aldolase SCO2393 (step 4), the enolase SCO3096 (step 9), and the pyruvate kinases SCO2014 and SCO5423 (step 10). Proteins of group II, upregulated in Pi proficiency, included the glucose kinase SCO2126 (step 1), phosphofructokinase SCO5426 (step 3), the glyceraldehyde-3-phosphate dehydrogenases SCO7511 and SCO1947 (GAPDH, step 6), the phosphoglycerate kinase SCO1946 (PGK, step 7), the phosphoglycerate mutase SCO4470 (step 8), and the enolase SCO7638 (ENO, step 9). Proteins of group III, downregulated in Pi proficiency/upregulated in Pi limitation, included the glucose 6 phosphate isomerase SCO6659 (GPI, step 2), the phosphofructokinase SCO1957 (PFK, step 3), ortholog of the phosphofructokinases SCO1214 (group I) and SCO5426 (group II), and the triose phosphate isomerase SCO1945 (TPI, step 5). Glc6P being the entry point of the pentose phosphate pathway (PPP), the low abundance of the GPI/SCO6659 as well as that of the PFK/SCO1957 and TPI/SCO1945, in Pi proficiency (group III), might contribute to direct a larger proportion of the glycolytic flux toward the PPP in such condition. Conversely, the carbon flux through the PPP should be reduced in Pi limitation, and this might contribute to lower NADP generation and thus higher oxidative stress in such condition.

Cluster B includes 10 glycolytic enzymes that were more abundant in *SC* than in the *SL* strains and only slightly more abundant in Pi limitation than in Pi proficiency in that strain. This cluster includes some enzymes orthologous of those present in cluster A and potentially catalyzing the glycolytic steps 2 (glucose 6 phosphate isomerase, SCO1942/SCO6659), 4 (fructose 6 bisphosphate aldolase SCO3649/SCO2393), 6 (GAPDH SCO7040/SCO7511 and SCO1947), 7 (phosphoglycerate kinase SCO6466/SCO1946), and 8 (phosphoglycerate mutase SCO4209/SCO4470). Interestingly, the fructose 1, 6 bisphosphatase SCO5047, a gluconeogenic enzyme, belongs to this cluster suggesting that enzymes of this cluster that are abundant in *SC* act as gluconeogenic enzymes.

Interestingly, the glucokinase SCO2126 (step 1) and the phosphoglycerate mutase SCO4470 (step 8) of cluster A were less abundant in the *pptA* mutant strain than in the *wt* strain of *SL* in Pi limitation, whereas in contrast SCO1947 (glyceraldehyde dehydrogenase, step 6) and SCO1946 (phosphoglycerate kinase, step 7) of cluster A were slightly more abundant in the *pptA* mutant than in the *wt* strain of *SL*. The glucose kinase activity consumes ATP, whereas steps 6 and 7 of glycolysis generate NADH and ATP, respectively. The respective downregulation and upregulation of these enzymes in the *pptA* mutant might be linked to the energetic deficit of this strain. Energy-generating enzymes would be upregulated, and ATP-consuming enzymes would be downregulated to maintain the energetic balance of this strain. Consistently, one notes that three other ATP-consuming enzymes of cluster B, the sugar kinase SCO6260, the pyruvate phosphate dikinase SCO2494, and the phosphofructokinase SCO2119, were also less abundant in the *pptA* mutant than in the *wt* strain in Pi limitation.

The lower abundance of glycolytic enzymes in *SC*, compared to the *SL* strains, suggested that *SC* has a weaker glycolytic activity than the latter. This is consistent with previous studies demonstrating that *SC* consumes preferentially the amino acids present in the R2YE medium as carbon source rather than glucose and is thus forced to undergo gluconeogenesis (Millan-Oropeza et al., 2017). *SC* was reported to have a lower PL and triacylglycerol content than *SL* (Lejeune et al., 2021) indicating that the gluconeogenic metabolism of *SC* generates lower amounts of acetylCoA than the glycolytic metabolism of *SL*. Furthermore, and most importantly, the weak carbon flux through glycolysis in *SC* is predicted to lead to a reduced carbon flux through the PPP that is the main NADPH-generating route within the cell.

##### Pentose Phosphate Pathway

**FIGURE 3:**
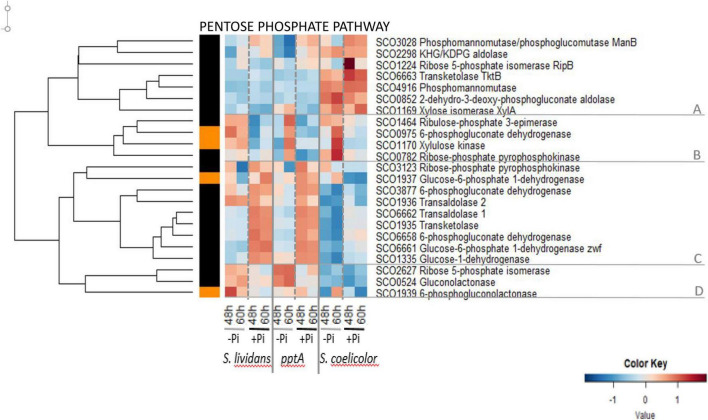
Heatmap representation of enzymes of the pentose phosphate pathway (PPP).

The heatmap of Figure 3 indicates that the abundance pattern of enzymes of the PPP could be divided into four main clusters. Most of the seven enzymes of cluster A that belong to the non-oxidative part of the PPP were more abundant in SC than in the *SL* strains and belonged to group I since their abundance did not vary with Pi availability. The four enzymes of cluster B were more abundant in Pi limitation than in Pi proficiency (group III) in the wt strain of *SL* at both time points as well as in the pptA mutant and SC but mainly at 60 h. This cluster includes enzymes belonging to the non-oxidative part of the PPP. In contrast, the nine enzymes of cluster C were far more abundant in the *SL* strains than in SC and more abundant in Pi proficiency than in Pi limitation (group II) especially in the *SL* strains. This abundance pattern is reminiscent of that of proteins of the Pho regulon under the negative control of PhoP, This cluster includes enzymes of the oxidative part of the PPP that generate NADPH, the glucose-6-phosphate dehydrogenases SCO1937 and SCO6661 (zwf, converting glucose-6P into 6-phosphogluconolactone), the glucose-1-deshydrogenase SCO1335 (converting glucose into gluconolactone), the 6-phosphogluconate dehydrogenases SCO3877 and SCO6658, and enzymes of the non-oxidative part of PPP, the transaldolases SCO1936 and SCO6662, and the ribose-phosphate pyrophosphokinase SCO0782 involved in nucleotides (purines and pyrimidines) and NAD/NADP synthesis.

The low glycolytic activity of *SC* results inevitably into a reduced carbon flux through the PPP, and one notes that enzymes of the oxidative part of the PPP (Figure 3, cluster C) were far less abundant in *SC* than in the *SL* strains. The low activity of the oxidative part of the PPP in *SC* should lead to a reduced generation of NADPH. NADPH is a necessary co-factor of thioredoxins reductases, enzymes that play an important role in the resistance to oxidative stress since they catalyze the re-oxidation of reduced thioredoxins, small proteins involved in the reduction of illegitimate di-sulfur bonds formed in proteins in conditions of oxidative stress (Paget et al., 1998). Consequently, a reduced generation of NADPH in *SC* is predicted to lead to high oxidative stress that was proposed to be an important trigger of ACT biosynthesis in *SC* (Esnault et al., 2017; Virolle, 2020). Consistently, reports in the literature mentioned that a reduced carbon flux through PPP, a consequence of the deletion of genes encoding the first enzymes of the PPP, the isoenzymes glucose 6-P-dehydrogenase (zwf1/SCO6661 zwf2/SCO1937), and/or the 6-phosphogluconolactonase (devD/SCO1939), resulted in increased ACT levels (Butler et al., 2002), whereas conversely, the over-expression of these enzymes led to reduced ACT levels (Jin et al., 2017). At last, it should be stressed that, the abundance pattern of enzymes of the PPP being similar in the two *SL* strains, a lower activity of the PPP cannot account for a higher oxidative stress triggering ACT production in the *pptA* mutant.

##### Tricarboxylic Acid Cycle

The catabolism of amino acids can yield directly acetylCoA (Trp, Tyr, Phe, Thr, Leu, Ile, and Lys), pyruvate (Ala, Gly, Cys, Ser, Thr, Trp), or intermediates of the tricarboxylic acid (TCA) cycle such as α-ketoglutarate (Pro, Arg, His, Gln, Glu), succinate (Thr, Ile, Val, Met), fumarate (Tyr, Phe, Asp), or oxaloacetate (Asp, Asn). Proline being the most abundant amino acids present in R2YE, its conversion into α-ketoglutarate feeds the TCA cycle and supports gluconeogenesis. The catabolism of amino acids and key reactions of the TCA cycle are predicted to generate more reduced co-factors than glycolysis (Millan-Oropeza et al., 2017), and the re-oxidation of the latter by the respiratory chain likely contributes to the high energetic state of *SC* (Esnault et al., 2017). However, one cannot totally exclude that the slower growth rate of *SC*, compared to the *SL* strains, results in ATP saving, contributing to the high ATP content of this strain.

**FIGURE 4:**
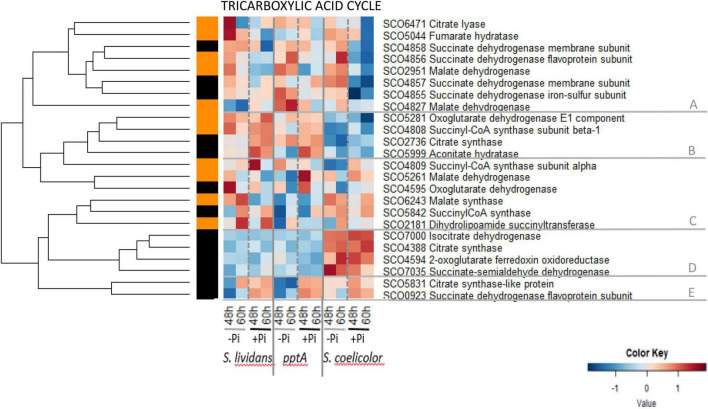
Heatmap representation of enzymes of tricarboxylic acid cycle (TCA).

The heatmap of Figure 4 indicated that the abundance pattern of enzymes of the TCA cycle is complex but can still be divided into five main clusters. Cluster A contains eight enzymes that had a similar abundance in the three strains in Pi limitation but that were less abundant in *SC* than in the *SL* strains in Pi proficiency. This cluster includes the succinate dehydrogenase (sub-units SCO4855–58) that generates FADH2, the fumarate hydratase SCO5044, the malate dehydrogenases SCO2951 and SCO4827 that generate NADH, and the ATP-dependent citrate lyase SCO6471 that catalyzes the interconversion of citrate into oxaloacetate and acetylCoA that can be used for fatty acid biosynthesis. Cluster B contains four enzymes that were far more abundant in the *SL* strains than in *SC* in both Pi conditions and only slightly more abundant in Pi proficiency than in Pi limitation (group II). It includes the citrate synthase SCO2736, the aconitate hydratase SCO5999, the α-ketoglutarate dehydrogenase E1 component SCO528 that generates NADH, and the α subunit (SCO4808) of the succinyl-CoA synthase that generates guanosine triphosphate (GTP). Cluster C contains six enzymes that have a complex abundance pattern. It includes sub-units of ATP-generating succinylCoA synthases SCO4809 and SCO5842, as well as the malate dehydrogenase SCO5261, the α-ketoglutarate dehydrogenase SCO4595, and the dihydrolipoamide succinyltransferase SCO2181 that generates NADH. The malate synthase SCO6243 of the glyoxylate cycle also belongs to this cluster and is the only enzyme showing a clear upregulation in Pi limitation in the *wt* strain of *SL* (but not in the *pptA* mutant). Cluster D includes four enzymes that were clearly far more abundant in *SC* than in the *SL* strains, and their abundance did not vary with Pi availability (group I). This cluster includes the citrate synthase SCO4388, isocitrate dehydrogenase SCO7000, the α-ketoglutarate ferredoxin oxidoreductase SCO4594 that belongs to the α-ketoglutarate dehydrogenase complex, and the succinate-semi aldehyde dehydrogenase SCO7035 that converts succinate-semi aldehyde, originating from GABA deamination, into succinate. Three of these enzymes catalyze reactions generating NADH. These enzymes are thus likely to contribute to the previously reported high ATP content of *SC* (Esnault et al., 2017). Cluster E contains two enzymes that were more abundant in Pi proficiency than in Pi limitation (group II) in the three strains. It includes the citrate synthase-like protein SCO5831 and the FADH2-generating succinate dehydrogenase SCO0923.

These data revealed that most enzymes of the TCA cycle did not show a strong regulation by Pi availability in the *SL* strains even if eight were slightly upregulated in Pi limitation (cluster A and SCO6243) and seven were slightly upregulated in Pi proficiency (clusters B and E). The abundance of proteins of clusters B and D was highly contrasted between *SC* and the *SL* strains. The low abundance of proteins of cluster B in *SC* might be compensated by the high abundance of orthologous proteins of cluster D. Interestingly, orthologs of citrate synthase, the entry point of the TCA cycle, are present in clusters A, B, D, and E and thus show different regulatory patterns. Such genetic redundancy likely serves regulatory diversity to adjust to different physiological conditions.

Reduced co-factors generated by the TCA donate electrons to the respiratory chain that yields ATP as well as potentially reactive oxygen species (ROS)/reactive nitrogen species (RNS) (oxidative stress); we thus next examined the abundance pattern of enzymes playing a role in oxidative phosphorylation in the three strains.

#### Oxidative Phosphorylation/Respiration

**FIGURE 5:**
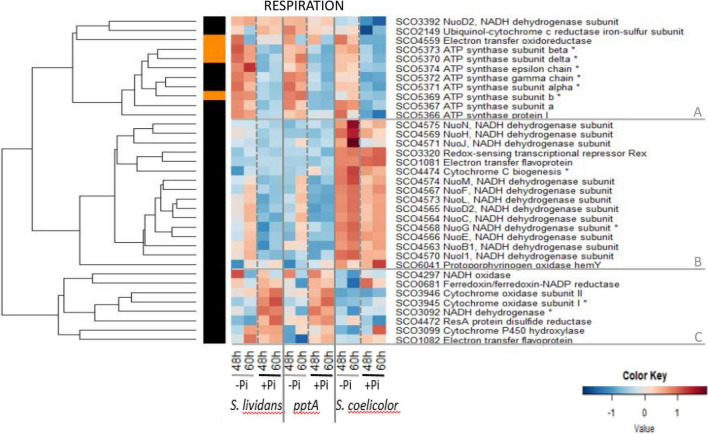
Heatmap representation of proteins involved in respiration.

Among the 35 enzymes of the respiratory chain detected, 25 are known to be under the control of the Rex/SCO3320 regulator constituting the Rex regulon.^1^ Rex is known to repress the transcription of genes encoding subunits of the NADH dehydrogenase (complex I, nuoABCDEFG HIJKLMN/SCO4562–75) and of the ATP synthase (atpIBEFHAGDC/SCO5366–74), as well as that of the uroporphyrinogen-III synthase (hemACD/SCO3317–19), of proteins involved in the biogenesis of cytochrome c (SCO4472–75), of the cytochrome d ubiquinol oxidase/permease (CydABCD/SCO3945–47), and of the putative H + translocating pyrophosphate synthase hppA/SCO3547. The repressing effect of Rex is thought to be alleviated when NADH is abundant within the cell. Such regulation adjusts the abundance of proteins of the respiratory chain to the amount of NADH generated by the cellular metabolism (Brekasis and Paget, 2003). The heatmap of Figure 5 indicates that the abundance pattern of respiratory enzymes fall into three main clusters, A, B, and C.

**FIGURE 6:**
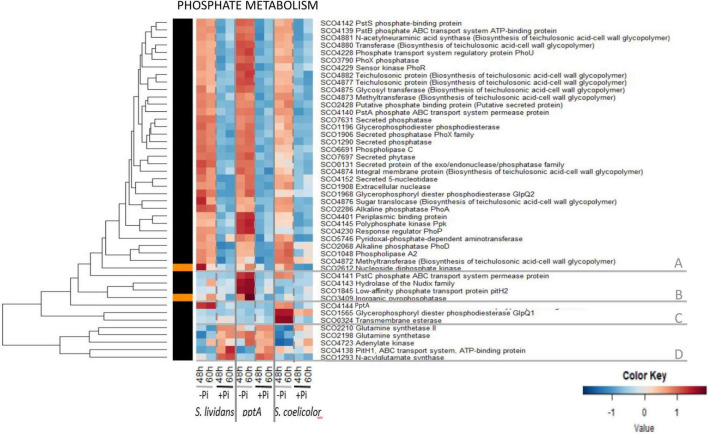
Heatmap representation of proteins belonging to the pho regulon according to Allenby et al. (2012).

Cluster A includes most sub-units of the ATP synthase (SCO5366–SCO5374). Proteins of this cluster show a similar abundance pattern in the three strains. These proteins belong to group III, being clearly upregulated in Pi limitation. This high expression is thought to result from the relieving of the Rex-dependent repression in a condition of high NADH availability that obviously occurs in a condition of Pi limitation. However, this abundance pattern being reminiscent of that of proteins of the Pho regulon under the positive control of PhoP (Figure 6, cluster A), the expression of these genes might result both from the NADH-dependent relieving of Rex repression and from a PhoP-dependent activation. This also indicated that an activation of the oxidative metabolism takes place in Pi limitation as an attempt to restore the cellular energetic balance, as proposed previously (Esnault et al., 2017; Virolle, 2020).

Cluster B includes most sub-units of the NADH dehydrogenase complex (complex I, nuoABCDEFGHIJKLMN/SCO4562–75) of the respiratory chain as well as the redox-sensing transcriptional repressor Rex SCO3320 (Brekasis and Paget, 2003). Sub-units of complex I, thought to generate ROS (Vinogradov and Grivennikova, 2016), were clearly far more abundant in *SC* than in the *SL* strains in both Pi conditions and only slightly more abundant in Pi limitation than in Pi proficiency in *SC* as well as in the *SL* strains but mainly late in growth (60 h) in the latter. At last and in contrast to proteins of cluster A, proteins of cluster C were more abundant in Pi proficiency than in Pi limitation (group II) in the *SL* strains, whereas most of these proteins had a low and similar abundance in both Pi conditions in *SC*. This abundance pattern is reminiscent of that of proteins of the Pho regulon under the negative control of PhoP (Figure 6, cluster D). This cluster includes sub-units of the cytochrome oxidase (SCO3945–46), the last enzyme of the respiratory chain that receives electrons from cytochrome c molecules and transfers them to O_2_ to yield H_2_O.

This study unexpectedly revealed that the Rex regulator SCO3320 and sub-units of the NADH dehydrogenase complex I (cluster B) were far more abundant in *SC* than in the *SL* strains, in both Pi conditions, suggesting a total de-repression of their expression. Such de-repression might be due to the existence of higher level of NADH in both Pi conditions in *SC*, compared to the *SL* strains, linked to its highly active oxidative metabolism. It also implies that Rex autoregulates negatively its own synthesis. It is noteworthy that in the *SL* strains, the sub-units of the ATP synthase were strongly upregulated in a condition of Pi limitation, whereas those of the NADH dehydrogenase complex I were only weakly upregulated in that condition and mainly late in growth (60 h). This might indicate that Rex has a higher affinity for its operator sites in the promoter region of the genes encoding the sub-units of NADH dehydrogenase complex I than in those of the ATP synthase. Consequently, the relieving of the Rex-mediated repression of enzymes of NADH dehydrogenase complex I would thus require higher level of NADH than that of the sub-units of the ATP synthase, levels that can only be reached in *SC*.

However, one can also envisage that the expression of the genes encoding the sub-units of the NADH dehydrogenase complex I are under the negative control of another regulator, besides Rex. This regulator would be present and functional in the *SL* strains but absent or not functional in *SC*.

In any case, the great abundance of sub-units of complex I of the respiratory chain and the low abundance of enzymes of cluster C (Figure 5) as well as menaquinone (Supplementary Figure 14A) in *SC* is predicted to lead to an alteration of the usual stoichiometry between these elements of the respiratory chain and of the ATP synthase. This might alter the proper functioning of the respiratory chain, leading to electron leakage toward secondary acceptors, generating ROS/RNS and thus oxidative stress (Imlay, 2003). Interestingly, in *SC*, the onset of ACT biosynthesis was shown to coincide with an abrupt drop in the intracellular ATP concentration (Esnault et al., 2017). Coincidence does not mean causality but since ACT has anti-oxidant properties thought to be due to its ability to capture excess electrons of ROS/RNS with its quinone groups (Lu et al., 2006), it might also be able to capture electrons of the respiratory chain conferring to ACT an “anti-respiratory” function (Esnault et al., 2017). In a condition of Pi limitation, ACT would thus also reduce ATP generation to adjust it to low Pi availability (Virolle, 2020).

Respiration and ATP synthesis being closely linked and requiring phosphate availability, we next examined the abundance features of proteins involved in phosphate metabolism.

#### Phosphate Metabolism

The heatmap of Figure 6 indicated that the abundance pattern of the 46 proteins listed as belonging to the Pho regulon in Allenby et al. (2012) could be divided into five main clusters.

Cluster A includes 35 proteins showing similar abundance features in the three strains, being far more abundant in Pi limitation than in Pi proficiency (group III). However, a close inspection of these heatmaps confirmed that, in a condition of Pi limitation, the regulator PhoP and most proteins under its positive control were more abundant in the *pptA* mutant than in the *wt* strain of *SL* (Shikura et al., 2021) and less abundant in *SC* than in *SL* (Millan-Oropeza et al., 2020). Cluster B includes four proteins involved in phosphate re-cycling/scavenging and uptake, the hydrolase of the Nudix family SCO4143 encoded by the gene located downstream of *pptA*, the inorganic pyrophosphatase SCO3409 as well as the permease of the high-affinity phosphate transporter PstC/SCO4141, and the low-affinity phosphate transporter pitH2/SCO1845. The high abundance of these proteins in the *pptA* mutant, but not in *SC* (except PstC) nor in the *wt* strain of *SL*, indicates that the *pptA* mutant suffers from phosphate stress because of its inability to use its polyphosphate stores as source of free Pi and/or energy (Shikura et al., 2021). Cluster C includes PptA itself that was abundant in the *wt* strain of *SL* and in *SC*, to a lesser extent, in Pi limitation. This cluster also contains the glycerophosphoryl diester phosphodiesterase SCO1566/GlpQ1 and the trans-membrane esterase SCO0324 that were highly abundant in *SC*, but not in the *SL* strains, in Pi limitation. These proteins are involved in the degradation of PLs and may provide either Pi and/or acetylCoA for gluconeogenesis and/or TCA fueling in *SC.* Cluster D includes the glutamine synthases SCO2210 and SCO2198, the putative N acyl glutamate synthase SCO1293, the adenylate kinase SCO4723, and the low-affinity phosphate transporter PitH1 (SCO4138) that were clearly more abundant in Pi proficiency than in Pi limitation in the *SL* strains as well as in *SC*, to a lesser extent. The expression of these proteins is known to be under the negative control of PhoP (Sola-Landa et al., 2008, 2013), so their high abundance in Pi proficiency is consistent with the absence of PhoP in such condition.

This study revealed that the regulation of abundance of proteins of the Pho regulon by Pi availability was mostly conserved in the three strains. Our data are consistent with what could be expected from proteins whose expression is under the major (positive or negative) control of PhoR/PhoP. However, this study also confirmed a specific abundance pattern of proteins belonging to the Pho regulon in each of the three strains. Proteins under the positive control of PhoP were more abundant in Pi limitation than in Pi proficiency (group III) in the three strains but were more and less abundant in the *pptA* mutant of *SL* and in *SC*, respectively, than in the *wt* strain of *SL*. Proteins under the negative control of PhoP (cluster C) were more abundant in Pi proficiency than in Pi limitation (group II) in the three strains but were less abundant in *SC* than in the *SL* strains. Overall, the differential abundance of proteins of the Pho regulon in Pi limitation and proficiency was less contrasted in *SC* than in the *SL* strains, likely because the abundance of PhoR/PhoP was less contrasted in these two Pi situations in *SC* than in the *SL* strains. Conditions of Pi proficiency are correlated with high ATP levels (Esnault et al., 2017) and with the low expression of the TCS PhoR/PhoP (Ghorbel et al., 2006a). Consequently, the previously reported high ATP content of *SC* in both Pi conditions is predicted to downregulate PhoR/PhoP expression in this strain and thus to be responsible for the reduced amplitude of regulation of genes of the Pho regulon between the two Pi conditions in *SC*. This regulatory effect might be mediated by the Large ATP-binding regulators of the LuxR family (LAL regulators), that were shown to play a negative role in the regulation of PhoR/PhoP expression (Guerra et al., 2012). These regulators are thought to sense ATP level, and their repressive effect of *phoR/phoP* expression might be reinforced when the intracellular ATP concentration is high, as in *SC*. Since phosphate and nitrogen metabolism are usually coordinated, we next examined the abundance of proteins involved in nitrogen assimilation.

#### Nitrogen Assimilation

**FIGURE 7:**
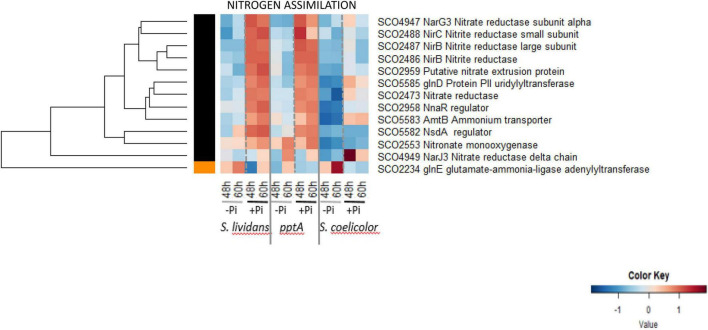
Heatmap representation of enzymes involved in nitrogen assimilation.

The heatmap of Figure 7 indicated that 12 of the 13 proteins listed as involved in nitrogen assimilation were clearly upregulated in Pi proficiency in the *SL* strains, but the amplitude of this upregulation was strongly reduced in *SC*. Such abundance pattern is reminiscent of that of proteins of the Pho regulon under the negative control of PhoP (Figure 6, cluster D) and suggests that *SC* assimilates nitrogen less efficiently than *SL*, at least in Pi proficiency. Our data indicated that phosphate and nitrogen assimilation were coregulated in *SL*, but this coregulation was partially impaired in *SC*. Interestingly the adenylyltransferase GlnE/SCO2234 that modulates the activity of glutamine synthetase I in response to the nitrogen availability (Reuther and Wohlleben, 2007) is the only protein upregulated in Pi limitation (group III) in *SL* and *SC*.

In summary, our results indicated that the abundance of proteins of metabolic pathways proposed to cause or remedy oxidative stress was different in *SC* and in the two *SL* strains. In *SC*, the high abundance of complex I of the respiratory chain and possible dysfunction of the respiratory chain due to the altered stoichiometry between its constitutive elements likely generates oxidative stress. Furthermore, a reduced carbon flux through the PPP linked to a weak glycolytic flux would generate insufficient amount of NADPH to fight the high oxidative stress of this strain. Such features of *SC* that are consistent with previous published studies (Sulheim et al., 2020) do not exist in the *pptA* mutant and thus cannot explain the induction of ACT biosynthesis in this strain, in a condition of Pi limitation. However, interestingly, a close inspection of our data revealed a major difference between the *wt* strain of *SL* and its *pptA* mutant concerning the abundance of enzymes involved in the metabolism of NADP(H).

#### NADP(H) Metabolism

**FIGURE 8:**
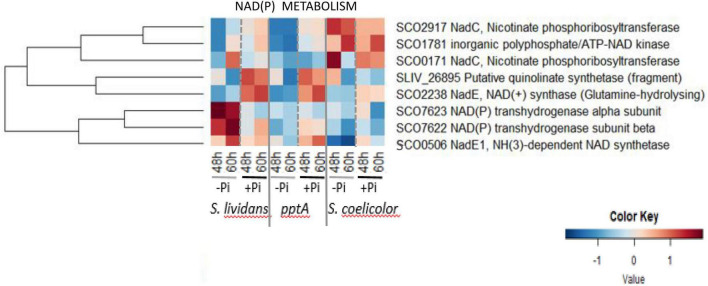
Heatmap representations of proteins involved in NAD metabolism.

The heatmap of Figure 8 revealed that in Pi limitation, SCO7622 and SCO7623, constituting the sub-units of a transhydrogenase, were far more abundant in the *wt* strain of *SL* than in its *pptA* mutant and in *SC*. *In vivo*, the transhydrogenase catalyzes the conversion of NADPH, mainly generated by the PPP, into NADH in order to regenerate NADP + and reduce excess of NADPH. Since the expression of enzymes is usually induced by their substrate, the low abundance of SCO7622–23 in the *pptA* mutant and in *SC* suggests a low abundance of NADPH in these two strains. As mentioned above, a low NADPH content linked to a low oxidative activity of the PPP was anticipated in *SC* but not in the *pptA* mutant. NADP is synthetized from NAD by NAD kinases using either ATP or polyphosphate (polyP) as phosphate and energy donors (Lindner et al., 2010). A single gene annotated as encoding an inorganic polyphosphate/ATP-dependent NAD kinase, *sco1781*/*ppnK*, was identified in the genome of *SC* and *SL*. Since PptA was proposed to be an accessory factor forcing polyP into a conformation suitable for their efficient use by various enzymes taking polyP as donor of phosphate and/or energy such as exo-polyphosphatases or general phosphatases or the polyphosphate kinase Ppk/SCO4145 (Chouayekh and Virolle, 2002; Ghorbel et al., 2006b; Shikura et al., 2021), PptA is also likely to be necessary for the efficient use of polyP, as donor of phosphate and energy, by the NAD kinase that phosphorylates NAD into NADP. We thus propose that in a condition of Pi and thus ATP limitation, the synthesis of a larger fraction of the cellular NADP by the NAD kinase would rely on polyP as donor of Pi and energy rather than on ATP, whereas it would be the opposite in Pi proficiency. In Pi limitation, NADP synthesis would be limited in the *pptA* mutant resulting in low NADPH levels and thus high oxidative stress responsible for the triggering of ACT biosynthesis. The amount of ACT produced by the *pptA* mutant being 2–3-fold lower than that of *SC* (Shikura et al., 2021), its deficit in NADPH and resulting oxidative stress might be less severe than that of *SC*. At last, it is noteworthy that NadC/SCO2917 enzyme involved in NAD biosynthesis and the NAD kinase/SCO1781 were far more abundant in *SC* than in the *SL* strains. The upregulation of these enzymes in *SC* might be linked to its deficit in NAD(P) due to the low activity of the PPP in this strain.

#### Specialized Metabolism

*SC* and *SL* are closely related strains possessing the same biosynthetic pathways potentially directing the biosynthesis of over 20 specialized metabolites including the products of the extensively studied cryptic polyKetide (CPK), calcium dependent antibiotic (CDA), undecylprodigiosin (RED), and actinorhodin (ACT) clusters. We previously demonstrated that ACT bears an “anti-oxidant” function (Millan-Oropeza et al., 2020), and this suggested that oxidative stress contributes to the triggering of ACT biosynthesis (Esnault et al., 2017; Virolle, 2020). The nature of the signals triggering the biosynthesis of the other specialized metabolites as well as of their function for the producing bacteria remains unknown. However, the abundance pattern of enzymes of these pathways might provide information on these matters.

The biosynthetic clusters of *SC* and *SL* can be classified into two major groups according to their response to Pi availability. Group A includes pathways only highly expressed in *SC* in both Pi conditions (group A1) or highly expressed in *SC* in both Pi conditions *and* in the *pptA* mutant in Pi limitation (group A2) or highly expressed in *SC* in both Pi conditions *and* in the two *SL* strains in Pi limitation (group A3). Group B includes pathways more expressed in Pi proficiency than in Pi limitation (group II) either in the three strains (coelibactin cluster, SCO7681–SCO7691) (Kallifidas et al., 2010) or mainly expressed in *SC* (CPK/coelimycin cluster, SCO6273–88) (Bednarz et al., 2021) or in the wild type strain of *SL* (dipeptide cluster, SCO6429–39).

**FIGURE 9:**
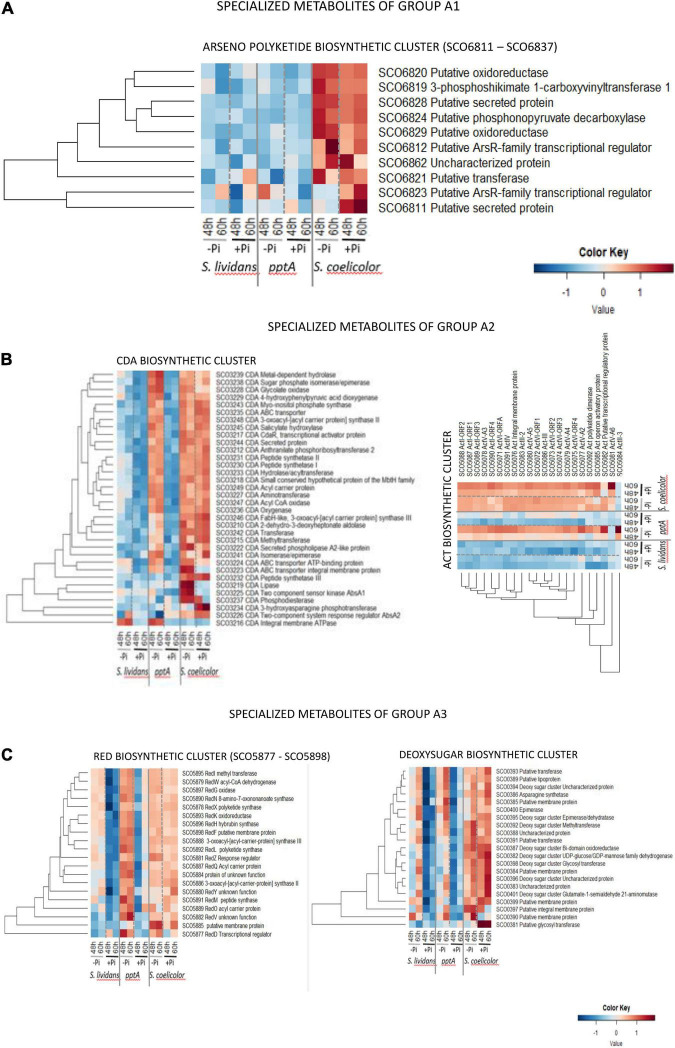
Heatmap representation of proteins of the arseno polyketide.biosynthetic cluster (group A1) **(A)**, of the polyketide cluster CDA and ACT.biosynthetic clusters (group A2) **(B)**, and of the RED and deoxysugar biosynthetic clusters (group A3) **(C)**.

Group A1 (Figure 9A) is only composed of proteins of the arseno polyketide biosynthetic pathway (SCO6811–SCO6837) that is mainly expressed in *SC* in both Pi conditions. The structure and function of the synthetized metabolite are not known, but the abundance pattern of this cluster being similar to that of enzymes of complex I of the respiratory chain in *SC* (Figure 5, cluster C), it might be involved in the regulation of the latter.

Group A2 (Figure 9B) is composed of the CDA and ACT clusters. Reports in the literature mentioned that the expression of the CDA and RED clusters starts at the transition phase, whereas the ACT cluster is expressed later at the stationary phase (Huang et al., 2001). In both cases, the biosynthesis of these molecules coincides with phases of growth slowdown/arrest. Among the 34 proteins of the CDA cluster detected (SCO3210–SCO3249, 39 proteins predicted), 26 were highly and similarly abundant in *SC* in both Pi condition. These proteins were also abundant in the *pptA* of *SL* but only in Pi limitation and poorly abundant in the *wt* strain of *SL* in both Pi conditions. Five of the eight remaining proteins of this cluster showed a regulation by Pi availability in *SC*. The TCS sensor kinase AbsA1/SCO3225, the ABC transporter integral membrane protein/SCO3223, the putative lipase SCO3219, and the phosphodiesterase/SCO3237 were upregulated in Pi limitation in *SC*, whereas the TCS response regulator AbsA2/SCO3226 as well as the 3-hydroxy asparagine phosphotransferase/SCO3234 were rather upregulated in Pi proficiency. In contrast, these six proteins as well as the peptide synthase III/SCO3232 were poorly abundant in the *pptA* mutant. This suggested that the expression of these proteins might be under the positive control of the TCS AbsA1/AbsA2 whose expression is weak in the *pptA* mutant strain. The weak abundance of the CDA peptide synthase III/SCO3232 in the *pptA* mutant raises the question of the amount and of the structure in the CDA molecule produced by the *pptA* mutant. At last, the putative cation transporting integral membrane ATPase/SCO3216 was poorly abundant in *SC* in both Pi conditions but abundant in the two *SL* strains in Pi limitation. The specific abundance pattern of SCO3216 suggested that it may not belong to the CDA cluster.

Group A3 (Figure 9C) is composed of the RED and deoxysugar clusters. The 20 proteins of the hybrid NRPS/PKS RED cluster detected (SCO5877–5801, 21 proteins predicted) were similarly abundant in *SC* in both Pi conditions at both time points. Interestingly, these proteins were more abundant in the *pptA* mutant than in *SC* but mainly in Pi limitation and were also present, but at a lower abundance, in the *wt* strain of *SL* in that condition. Most proteins of the deoxy sugar cluster (SCO0381–SCO0401, 21 proteins predicted) were more abundant in *SC* than in the *SL* strains in both Pi conditions, whereas these proteins were more abundant in Pi limitation than in Pi proficiency, especially at 60 h, in the two *SL* strains. The abundance of only two proteins of this cluster seems to vary with Pi availability in *SC*, the putative membrane protein/SCO0390, and the putative glycosyl transferase/SCO0381, downregulated and strongly upregulated, respectively, in Pi proficiency.

**FIGURE 10:**
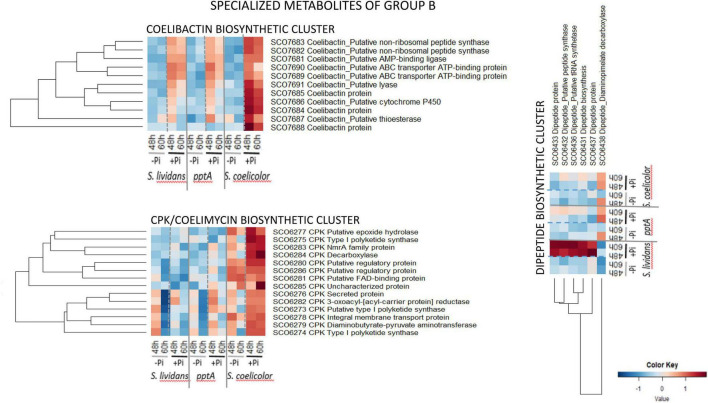
Heatmap representation of proteins of the coelibactin, CPK, and di-peptide biosynthetic clusters (group B).

Group B (Figure 10) is composed of pathways whose protein abundance is higher in Pi proficiency than in Pi limitation either in the three strains (coelibactin cluster, SCO7681–SCO7691) or mainly in *SC* (CPK/coelimycin cluster, SCO6273–88) or in the *wt* of *SL* (dipeptide cluster, SCO6429–39). The 11 proteins of the coelibactin cluster (SCO7681–SCO7691) (Kallifidas et al., 2010) were upregulated in Pi proficiency in the three strains but more strongly in *SC* than in the *SL* strains. The 14 proteins of the CPK cluster-detected (SCO6273–SCO6288, 16 proteins predicted) directing coelimycin synthesis (Bednarz et al., 2019, 2021) were more abundant in *SC* than in the *SL* strains and were upregulated in Pi proficiency in *SC*. At last the six proteins of the dipeptide biosynthetic cluster (SCO6429–38, 10 proteins predicted) were strongly upregulated in Pi proficiency but mainly in the *wt* strain of *SL*.

This study revealed that the various biosynthetic pathways directing the biosynthesis of specialized metabolites responded differently to Pi availability in *SC* and in the *SL* strains. The four clusters of groups A2 and A3 (CDA, ACT, RED, and deoxysugar biosynthetic clusters) were upregulated in Pi limitation in the *pptA* mutant of *SL* or in both *SL* strains but highly and constitutively expressed in both Pi conditions in *SC*. The biosynthesis of these molecules coincides with phases of growth slowdown/arrest, suggesting that their biosynthesis may be triggered by similar signals and that these molecules fulfill similar functions. The ionophore CDA is thought to create pores in the membrane that might lead to the dissipation of H + gradient and thus to the reduction of ATP synthesis. Similarly, several reports in the literature evoke also an energy-spilling function for molecules of the prodigiosin family (RED) in *Serratia marscesens* (Haddix et al., 2008). The function of these specialized metabolites, mainly synthetized in Pi limitation, might be to reduce ATP synthesis to adjust it to low Pi availability. Doing so, they would contribute to growth arrest as well as to programmed cell death and lysis (Tenconi et al., 2018).

In contrast two specialized metabolites of group B, CPK and coelibactin were rather upregulated in Pi proficiency in *SC* as well as in the *SL* strains, to a lesser extent, whereas the dipeptide biosynthetic cluster was strongly upregulated in Pi proficiency but only in the *wt* strain of *SL*. The biosynthesis of these metabolites is likely to respond to other types of signals than those of group A, and such signals remain to be determined as well as the function of these metabolites.

## Discussion

The benzochromane quinone ACT was previously shown to have anti-oxidant properties (Millan-Oropeza et al., 2020) suggesting that its biosynthesis might be triggered by oxidative stress. The aim of our proteomic study was thus to define the common features potentially responsible for high oxidative stress in the two ACT-producing strains, *SC* and the *pptA* mutant of *SL*. Interestingly, reports in the literature mention that other antibiotics have anti-oxidant properties and that their biosynthesis is induced in a condition of oxidative stress. In *Streptomyces* species these include tacrolimus produced by *Streptomyces tsukubaensis* (Pires et al., 2020), pimaricin produced by *Streptomyces natalensis* (Beites et al., 2011, 2015), and chromomycin produced by *Streptomyces flaviscleroticus* (Prajapati et al., 2018). Similar statements were made also for antibiotics produced by fungal species such as lovastatin produced by *Aspergillus terreus* (Miranda et al., 2014), penicillin produced by *Penicillium chrysogenum*, and cephalosporin produced by *Acremonium chrysogenum* (Bibian et al., 2020).

We inferred from our proteomic data that high oxidative stress and thus ACT biosynthesis resulted from totally different processes in the *pptA* mutant and in *SC*. PptA, which belongs to the Pho regulon, is thought to be an accessory factor forcing polyP into a conformation allowing their efficient use by various enzymes taking polyP as donor of phosphate and/or energy such as general phosphatases or exopolyphosphate phosphatases involved in the degradation of polyP into Pi (Shikura et al., 2021), the polyphosphate kinase Ppk/SCO4145 involved in the regeneration of ADP into ATP (Chouayekh and Virolle, 2002; Ghorbel et al., 2006b), and likely the NAD kinase SCO1781 able to phosphorylate NAD into NADP using either ATP or polyP as donor of Pi and energy. In a condition of phosphate and thus ATP limitation, the phosphorylation of NAD into NADP would mainly rely on polyphosphate. This phosphorylation would be inefficient in the *pptA* mutant resulting in an insufficient NADP(H) synthesis to fight oxidative stress.

In *SC*, high oxidative stress would be due to the noticeable high abundance of sub-units of complex I of the respiratory chain and by the altered stoichiometry between this enzyme and other enzymes of the respiratory chain (Figure 5, cluster C) and menaquinone (Supplementary Figure 14A), as well as of the ATP synthase (Figure 5, cluster A). In this context, the higher ACT production reported in Pi proficiency compared to Pi limitation in *SC* (Esnault et al., 2017) might be due to the worsening of this stoichiometric unbalance between the abundance of sub-units of complex I of the respiratory chain that remains high in both Pi conditions and that of the ATP synthase that is much lower in Pi proficiency than in Pi limitation (Figure 5, cluster A). In any case, our study indicated that the inactivation of genes encoding proteins playing a role in the resistance to oxidative stress should enhance the production of some specific antibiotics.

Interestingly, the abundance of proteins upregulated in a condition of Pi limitation in the *SL* strains and likely to be under the positive control of PhoP (group III) is only slightly reduced in *SC.* These include proteins belonging to phosphate metabolism (Figure 6, cluster A), sub-units of the ATP synthase (Figure 5, cluster A), some proteins contributing to cell wall biosynthesis and degradation (Supplementary Figure 15, cluster B), nucleotide biosynthesis (Supplementary Figure 7A, cluster B) and degradation (Supplementary Figure 7B, cluster C), vitamin B6 biosynthesis (Supplementary Figure 14B), and some transport systems (Supplementary Figure 15, cluster A). This indicated that PhoP is functional in *SC* and that the slightly lower abundance of the proteins under its positive control is due to the lower abundance of PhoR/PhoP in this strain (Millan-Oropeza et al., 2020). Furthermore this study revealed the upregulation of sub-units of the ATP synthase in a condition of Pi limitation (Figure 5, cluster A) confirming the previously proposed activation of the oxidative metabolism in that condition in order to re-establish the energetic balance of the cell (Esnault et al., 2017; Virolle, 2020). Such activation would generate NADH, impairing Rex binding and relieving the negative effect it exerts on the expression of its target genes including those encoding sub-units of the ATP synthase (Brekasis and Paget, 2003). In Pi proficiency, the phospholipids (PL) content of SC was shown to be lower than that of the SL strains (Lejeune et al., 2021). This is consistent with the noticeable lower abundance of the positive regulator of fatty acid biosynthesis, FasR/SCO2366 (Arabolaza et al., 2010), in this strain than in the *SL* strains (Supplementary Figure 2A, cluster B). This reduced fatty acids biosynthesis might result into an higher acetylCoA availability supporting the activation of the oxidative metabolism and thus the generation of oxidative stress that was proposed be an important trigger of the biosynthesis of antibiotics with anti-oxidant/anti-respiratory function.

In contrast, the amplitude of upregulation of numerous proteins in a condition of Pi proficiency in the *SL* strains is strongly reduced in *SC*. These include proteins belonging to glycolysis (Figure 2, cluster A), the PPP (Figure 3, cluster C), respiration (Figure 5, cluster C and Supplementary Figure 14A, menaquinone biosynthesis), nitrogen metabolism (Figure 6, cluster D and Figure 7, cluster B), amino acid biosynthesis (Supplementary Figure 3B, cluster C) and degradation (Figure 4A, cluster A), protein secretion (Supplementary Figure 6, cluster A), fatty acid/lipid biosynthesis (Supplementary Figure 2A, cluster B) and degradation (Supplementary Figure 2B, cluster A), nucleotide biosynthesis (Supplementary Figure 7A, cluster A), DNA replication and repair (Supplementary Figure 8, cluster A), iron/metal acquisition (Supplementary Figure 12, cluster B), colabalmine biosynthesis (Supplementary Figure 14B) and some transport systems (Supplementary Figure 16B). This indicated that besides the possible relieving of the repression by PhoP of the expression of the genes encoding these proteins in Pi proficiency, an activation of their expression takes place in the *SL* strains but not in *SC.* A putative transcriptional regulator might activate directly or indirectly the expression of these genes in a condition of Pi proficiency in the *SL* strains, but this regulator would be absent or non-functional in *SC.*

The cause of the low and high abundance of glycolytic and gluconeogenic enzymes, respectively, in *SC* is not known. A transcriptional regulator controlling negatively and positively the expression of glycolytic and gluconeogenic enzymes, respectively, was demonstrated in *Thermococcus kodakaraensis* (Kanai et al., 2007). A regulator with similar function might be present and functional in the *SL* strains but absent/non-functional in *SC.* Similarly, the cause of the extremely high abundance of sub-units of complex I of the respiratory chain in *SC* is not known, but it might result from very high level of NADH generated by the specific metabolism of *SC* that would impair Rex binding in the promoter region of the genes encoding these sub-units. This might indicate that Rex has higher affinity for its target sites located in the promoter region of the genes encoding sub-units of complex I than in those of the ATP synthase and thus requires higher level of NADH to be displaced from them. The specific metabolism of *SC* abundantly generates ATP, oxidative stress, and specific metabolites that remain to be identified. ATP has multiple regulatory roles (allosteric effector of enzymes and regulators, triggering agent of phosphorylation cascades etc.), its high abundance in *SC* is predicted to have drastic regulatory consequences on the proteome and metabolism of *SC* and might contribute to the lower expression of glycolytic enzymes and of the TCS PhoR/PhoP noticed in *SC*. Specific metabolites and high oxidative stress also play important regulatory roles that are difficult to predict. Altogether, the regulatory impact of these molecules will shape the utterly different proteins abundance patterns observed in the *SL* strains and in *SC*.

## Data Availability Statement

The datasets presented in this study can be found in online repositories. The names of the repository/repositories and accession number(s) can be found in the article/Supplementary Material.

## Author Contributions

CL performed all microbial cultivations and performed data analysis in R and prepared all the heatmaps. LS performed digestions, sample preparation for MS analysis, MS analysis, and MS data analysis. DC performed statistical analysis of label-free MS quantification data. VR supervised MS sample preparation and MS data analysis. M-JV conceptualized the project, wrote the manuscript, and obtained grants funding. All authors have read and agreed to the submitted version of the manuscript.

## Conflict of Interest

The authors declare that the research was conducted in the absence of any commercial or financial relationships that could be construed as a potential conflict of interest.

## Publisher’s Note

All claims expressed in this article are solely those of the authors and do not necessarily represent those of their affiliated organizations, or those of the publisher, the editors and the reviewers. Any product that may be evaluated in this article, or claim that may be made by its manufacturer, is not guaranteed or endorsed by the publisher.

## Footnotes

1
https://regprecise.lbl.gov/regulon.jsp?regulon_id=63393

## Supplementary Material

Supplementary Figure 1 Heatmap representation of proteins of pyruvate/acetylCoA and glycerol metabolism.

Supplementary Figure 2 Heatmap representation of enzymes involved in fatty acids biosynthesis **(A)** and degradation **(B)**.

Supplementary Figure 3 **(A,B)** Heatmap representation of proteins involved into amino acids biosynthesis.

Supplementary Figure 4 Heatmap representation of proteins involved into amino acids **(A)** and ureide **(B)** degradation.

Supplementary Figure 5 **(A,B)** Heatmap representation of enzymes involved into protein degradation.

Supplementary Figure 6 Heatmap representation of proteins involved into protein secretion.

Supplementary Figure 7 Heatmap representation of proteins involved into nucleotide biosynthesis **(A)** and degradation **(B)**.

Supplementary Figure 8 Heatmap representation of proteins involved into DNA replication and repair.

Supplementary Figure 9 Heatmap representation of proteins involved into chromosome segregation and cell division.

Supplementary Figure 10 Heatmap representation of proteins involved into cell wall biosynthesis and degradation.

Supplementary Figure 11 Heatmap representation of proteins involved into stress resistance **(A,B)** or belonging to the OdsR regulon **(C)**.

Supplementary Figure 12 Heatmap representation of proteins involved into iron/metal acquisition.

Supplementary Figure 13 Heatmap representation of proteins involved into terpenes biosynthesis and belonging to hopene and hydroxyectoine biosynthetic clusters.

Supplementary Figure 14 Heatmap representation of proteins involved in the biosynthesis of menaquinone and riboflavin **(A)**, of cobalamine and vitamine B6 **(B)**, of folate and heme **(C)**, of thiamine and co-enzyme A **(D)** and of biotine and molysdopterin **(E)**.

Supplementary Figures 15 Heatmap representation of transport systems induced in Pi limitation in the three strains **(A,B)** or only in the *S. lividans* strains **(C)** or only in *S. coelicolor* **(D)**.

Supplementary Figures 16 Heatmap representation of transport systems induced in Pi proficiency in the three strains **(A)** or mainly in the *S. lividans* strains **(B)**.

Supplementary Figures 17 Heatmap representation of transport systems highly abundant mainly in the *S. lividans* strains in both Pi conditions **(A)** or highly abundant mainly in *S. coelicolor* in both Pi conditions **(B)**.

Supplementary Figure 18 Heatmap representation of transport systems poorly abundant in the *pptA* mutant of *S. lividan*s in condition of Pi limitation **(A,B).**

Supplementary Figure 19 Heatmap representation of transport systems poorly abundant in *S. coelicolor* in condition of Pi proficiency.

Supplementary Table 1 Dataset of proteins quantified by label-free-based quantification methods and used to generate the heatmaps presented in Figures and Supplementary Figures.
