# Supplementary material for: A Proteomic Analysis Indicates That Oxidative Stress Is the Common Feature Triggering Antibiotic Production in Streptomyces coelicolor and in the pptA Mutant of Streptomyces lividans
Source: Front Microbiol. 2022 Mar 22;12:813993. doi: 10.3389/fmicb.2021.813993 (PMC8981147; doi:10.3389/fmicb.2021.813993)

Figure S1

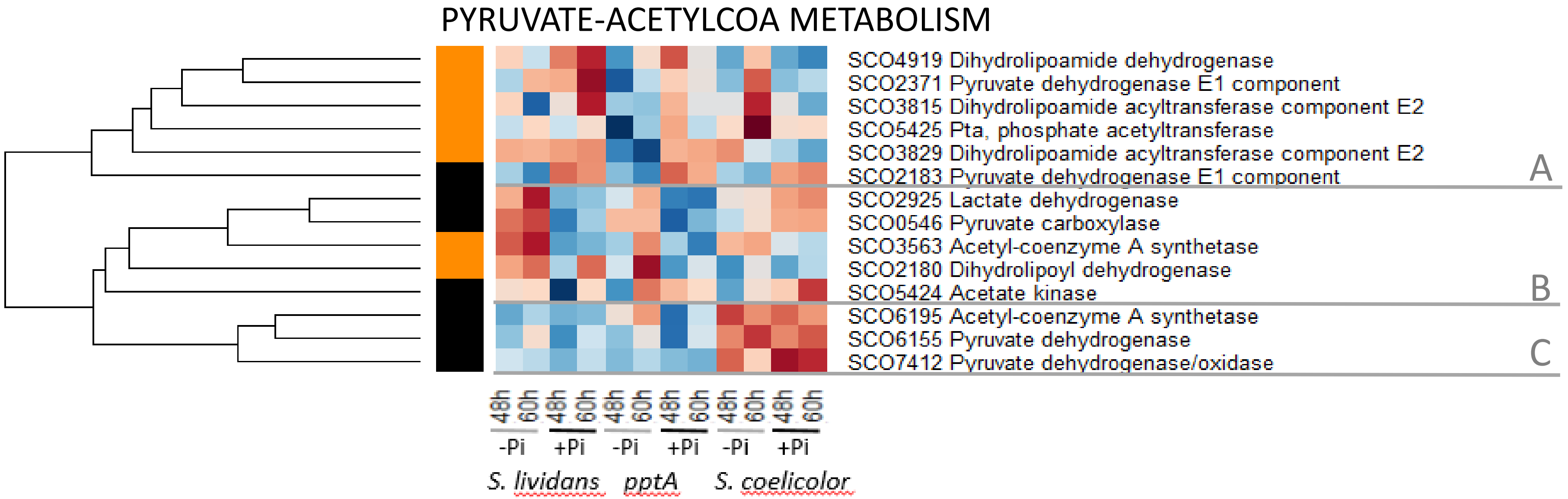

PYRUVATE-ACETYLCOA METABOLISM
SCO4919 Dihydrolipoamide dehydrogenase
SCO2371 Pyruvate dehydrogenase E1 component
SCO3815 Dihydrolipoamide acyltransferase component E2
SCO5425 Pta, phosphate acetyltransferase
SCO3829 Dihydrolipoamide acyltransferase component E2
SCO2183 Pyruvate dehydrogenase E1 component
A
SCO2925 Lactate dehydrogenase
SCO0546 Pyruvate carboxylase
SCO3563 Acetyl-coenzyme A synthetase
SCO2180 Dihydrolipoyl dehydrogenase
SCO5424 Acetate kinase
B
SCO6195 Acetyl-coenzyme A synthetase
SCO6155 Pyruvate dehydrogenase
SCO7412 Pyruvate dehydrogenase/oxidase
C
48h 60h 48h 60h 48h 60h 48h 60h 48h 60h 48h 60h
-Pi +Pi -Pi +Pi -Pi +Pi
S. lividans pptA S. coelicolor

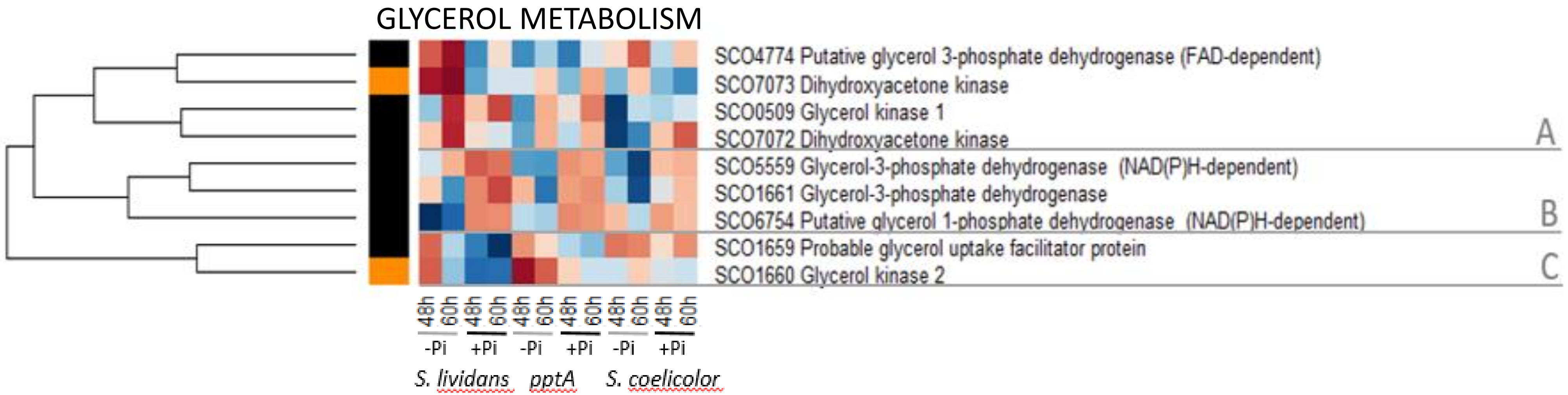

GLYCEROL METABOLISM
SCO4774 Putative glycerol 3-phosphate dehydrogenase (FAD-dependent)
SCO7073 Dihydroxyacetone kinase
SCO0509 Glycerol kinase 1
SCO7072 Dihydroxyacetone kinase
A
SCO5559 Glycerol-3-phosphate dehydrogenase (NAD(P)H-dependent)
SCO1661 Glycerol-3-phosphate dehydrogenase
SCO6754 Putative glycerol 1-phosphate dehydrogenase (NAD(P)H-dependent)
B
SCO1659 Probable glycerol uptake facilitator protein
SCO1660 Glycerol kinase 2
C
48h 60h 48h 60h 48h 60h 48h 60h 48h 60h 48h 60h
-Pi +Pi -Pi +Pi -Pi +Pi
S. lividans pptA S. coelicolor

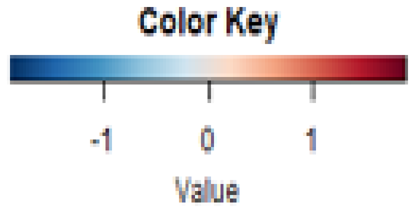

Color Key
-1 0 1
Value

Figure S2A

FATTY ACID / LIPID BIOSYNTHESIS

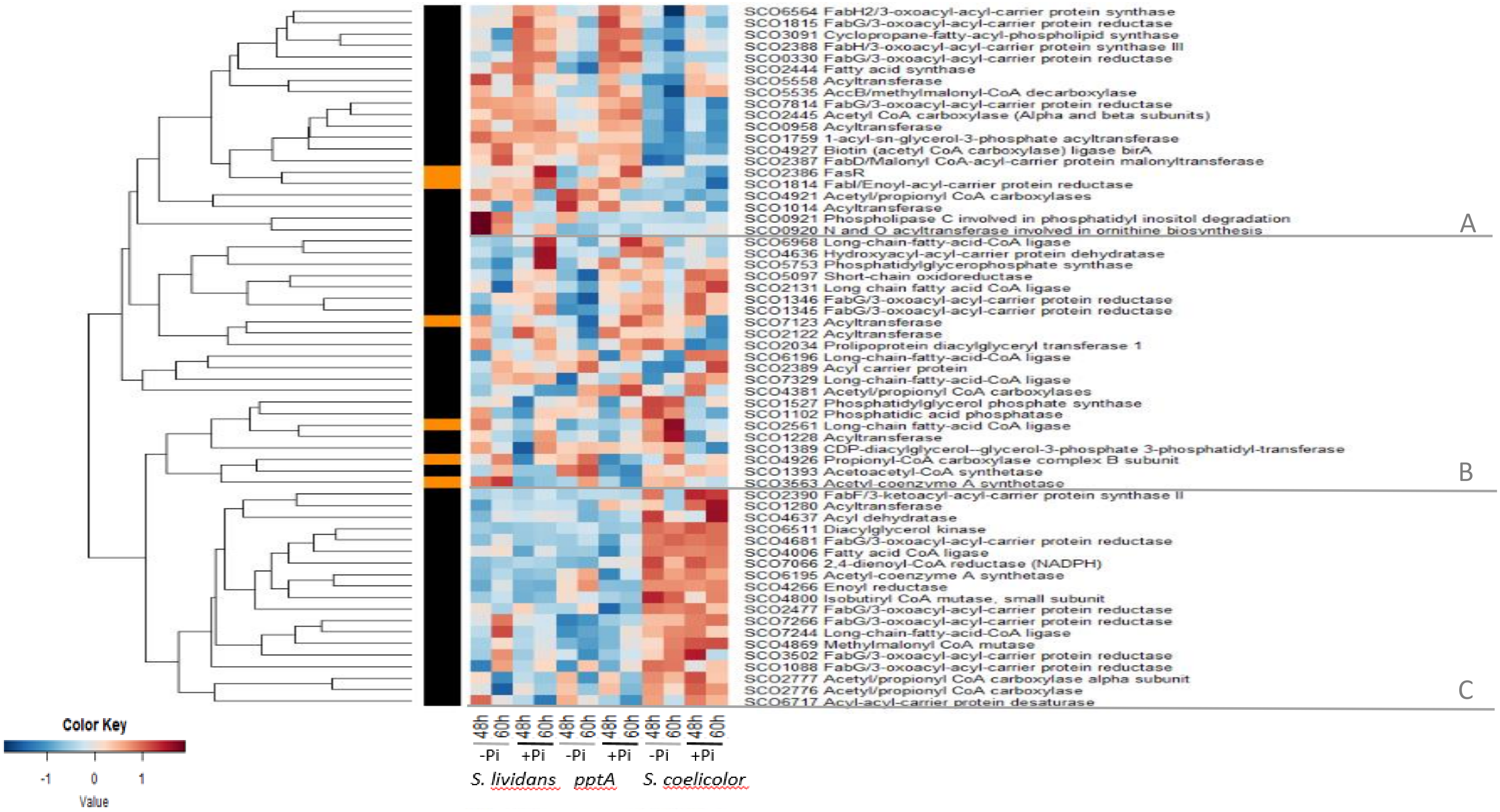

Figure S2B

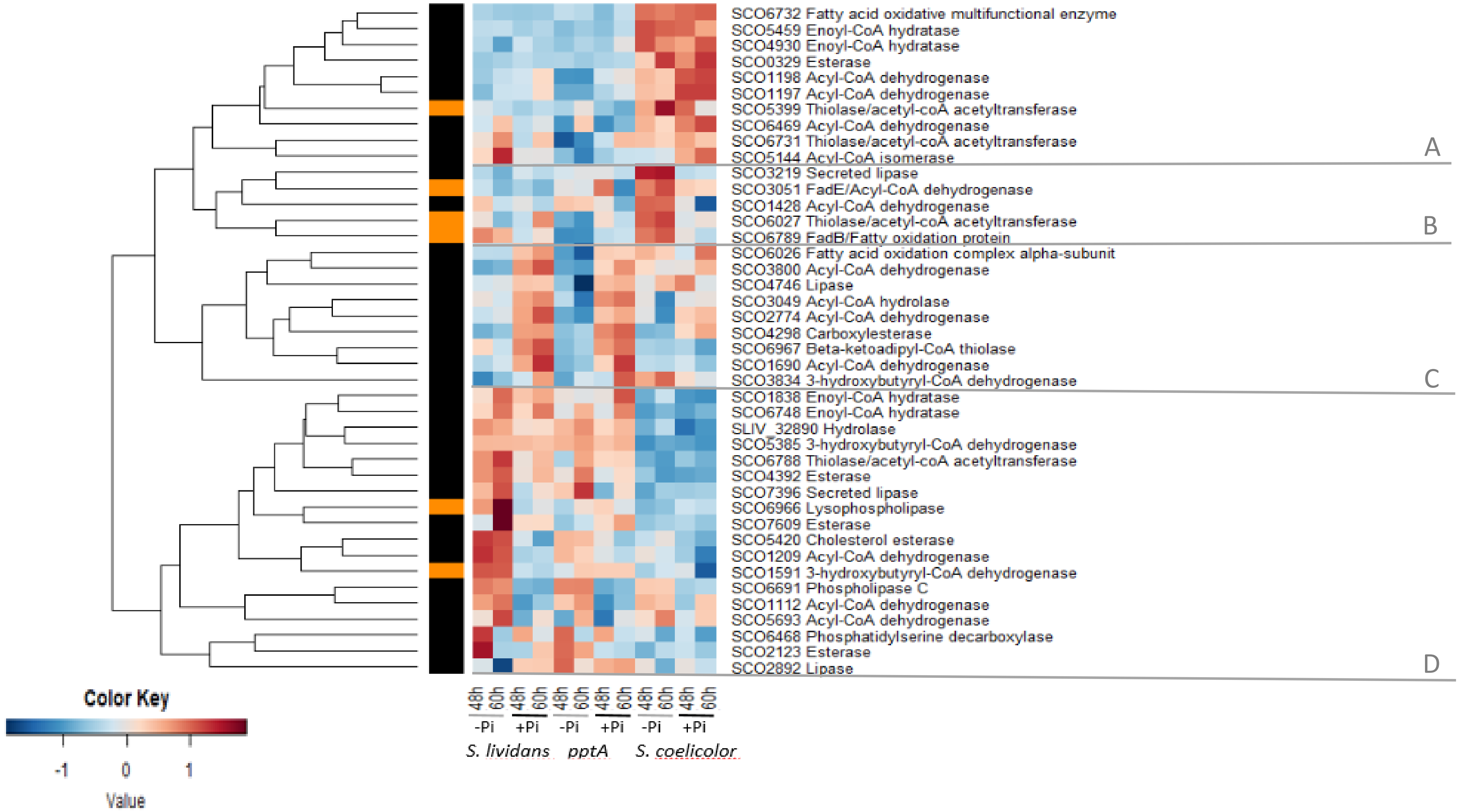
FATTY ACID / LIPID DEGRADATION
SCO6732 Fatty acid oxidative multifunctional enzyme
SCO5459 Enoyl-CoA hydratase
SCO4930 Enoyl-CoA hydratase
SCO0329 Esterase
SCO1198 Acyl-CoA dehydrogenase
SCO1197 Acyl-CoA dehydrogenase
SCO5399 Thiolase/acetyl-coA acetyltransferase
SCO6469 Acyl-CoA dehydrogenase
SCO6731 Thiolase/acetyl-coA acetyltransferase
SCO5144 Acyl-CoA isomerase
A
SCO3219 Secreted lipase
SCO3051 FadE/Acyl-CoA dehydrogenase
SCO1428 Acyl-CoA dehydrogenase
SCO6027 Thiolase/acetyl-coA acetyltransferase
SCO6789 FadB/Fatty oxidation protein
B
SCO6026 Fatty acid oxidation complex alpha-subunit
SCO3800 Acyl-CoA dehydrogenase
SCO4746 Lipase
SCO3049 Acyl-CoA hydrolase
SCO2774 Acyl-CoA dehydrogenase
SCO4298 Carboxylesterase
SCO6967 Beta-ketoadipyl-CoA thiolase
SCO1690 Acyl-CoA dehydrogenase
SCO3834 3-hydroxybutyryl-CoA dehydrogenase
C
SCO1838 Enoyl-CoA hydratase
SCO6748 Enoyl-CoA hydratase
SLIV_32890 Hydrolase
SCO5385 3-hydroxybutyryl-CoA dehydrogenase
SCO6788 Thiolase/acetyl-coA acetyltransferase
SCO4392 Esterase
SCO7396 Secreted lipase
SCO6966 Lysophospholipase
SCO7609 Esterase
SCO5420 Cholesterol esterase
SCO1209 Acyl-CoA dehydrogenase
SCO1591 3-hydroxybutyryl-CoA dehydrogenase
SCO6691 Phospholipase C
SCO1112 Acyl-CoA dehydrogenase
SCO5693 Acyl-CoA dehydrogenase
SCO6468 Phosphatidylserine decarboxylase
SCO2123 Esterase
SCO2892 Lipase
D
48h 60h 48h 60h 48h 60h 48h 60h 48h 60h 48h 60h
-Pi +Pi -Pi +Pi -Pi +Pi
S. lividans pptA S. coelicolor
Color Key
-1 0 1
Value

Figure S3A

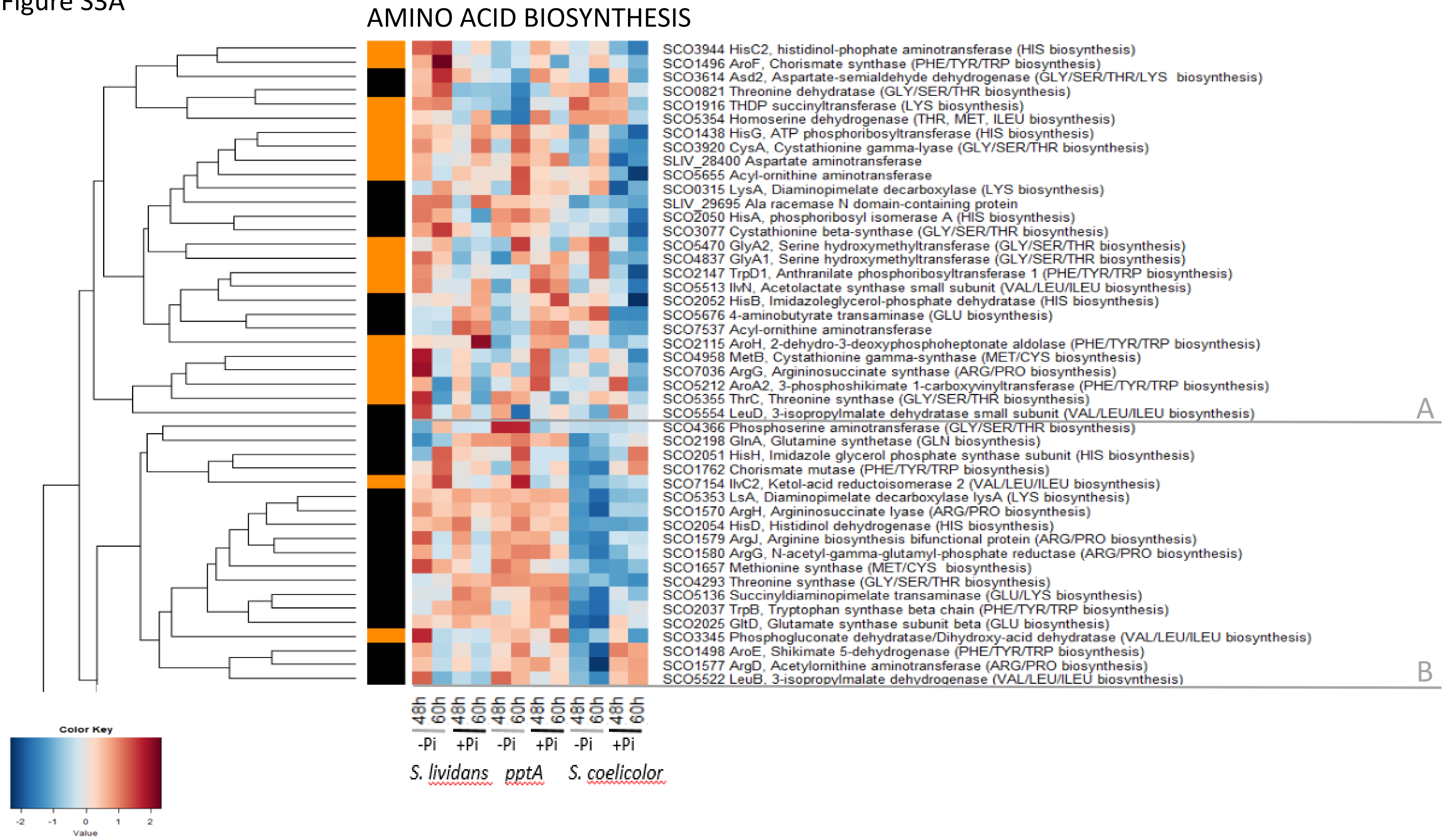
AMINO ACID BIOSYNTHESIS
SCO3944 HisC2, histidinol-phophate aminotransferase (HIS biosynthesis)
SCO1496 AroF, Chorismate synthase (PHE/TYR/TRP biosynthesis)
SCO3614 Asd2, Aspartate-semialdehyde dehydrogenase (GLY/SER/THR/LYS biosynthesis)
SCO0821 Threonine dehydratase (GLY/SER/THR biosynthesis)
SCO1916 THDP succinyltransferase (LYS biosynthesis)
SCO5354 Homoserine dehydrogenase (THR, MET, ILEU biosynthesis)
SCO1438 HisG, ATP phosphoribosyltransferase (HIS biosynthesis)
SCO3920 CysA, Cystathionine gamma-lyase (GLY/SER/THR biosynthesis)
SLIV_28400 Aspartate aminotransferase
SCO5655 Acyl-ornithine aminotransferase
SCO0315 LysA, Diaminopimelate decarboxylase (LYS biosynthesis)
SLIV_29695 Ala racemase N domain-containing protein
SCO2050 HisA, phosphoribosyl isomerase A (HIS biosynthesis)
SCO3077 Cystathionine beta-synthase (GLY/SER/THR biosynthesis)
SCO5470 GlyA2, Serine hydroxymethyltransferase (GLY/SER/THR biosynthesis)
SCO4837 GlyA1, Serine hydroxymethyltransferase (GLY/SER/THR biosynthesis)
SCO2147 TrpD1, Anthranilate phosphoribosyltransferase 1 (PHE/TYR/TRP biosynthesis)
SCO5513 IlvN, Acetolactate synthase small subunit (VAL/LEU/ILEU biosynthesis)
SCO2052 HisB, Imidazoleglycerol-phosphate dehydratase (HIS biosynthesis)
SCO5676 4-aminobutyrate transaminase (GLU biosynthesis)
SCO7537 Acyl-ornithine aminotransferase
SCO2115 AroH, 2-dehydro-3-deoxyphosphoheptonate aldolase (PHE/TYR/TRP biosynthesis)
SCO4958 MetB, Cystathionine gamma-synthase (MET/CYS biosynthesis)
SCO7036 ArgG, Argininosuccinate synthase (ARG/PRO biosynthesis)
SCO5212 AroA2, 3-phosphoshikimate 1-carboxyvinyltransferase (PHE/TYR/TRP biosynthesis)
SCO5355 ThrC, Threonine synthase (GLY/SER/THR biosynthesis)
SCO5554 LeuD, 3-isopropylmalate dehydratase small subunit (VAL/LEU/ILEU biosynthesis)
A
SCO4366 Phosphoserine aminotransferase (GLY/SER/THR biosynthesis)
SCO2198 GlnA, Glutamine synthetase (GLN biosynthesis)
SCO2051 HisH, Imidazole glycerol phosphate synthase subunit (HIS biosynthesis)
SCO1762 Chorismate mutase (PHE/TYR/TRP biosynthesis)
SCO7154 IlvC2, Ketol-acid reductoisomerase 2 (VAL/LEU/ILEU biosynthesis)
SCO5353 LsA, Diaminopimelate decarboxylase lysA (LYS biosynthesis)
SCO1570 ArgH, Argininosuccinate lyase (ARG/PRO biosynthesis)
SCO2054 HisD, Histidinol dehydrogenase (HIS biosynthesis)
SCO1579 ArgJ, Arginine biosynthesis bifunctional protein (ARG/PRO biosynthesis)
SCO1580 ArgG, N-acetyl-gamma-glutamyl-phosphate reductase (ARG/PRO biosynthesis)
SCO1657 Methionine synthase (MET/CYS biosynthesis)
SCO4293 Threonine synthase (GLY/SER/THR biosynthesis)
SCO5136 Succinyldiaminopimelate transaminase (GLU/LYS biosynthesis)
SCO2037 TrpB, Tryptophan synthase beta chain (PHE/TYR/TRP biosynthesis)
SCO2025 GltD, Glutamate synthase subunit beta (GLU biosynthesis)
SCO3345 Phosphogluconate dehydratase/Dihydroxy-acid dehydratase (VAL/LEU/ILEU biosynthesis)
SCO1498 AroE, Shikimate 5-dehydrogenase (PHE/TYR/TRP biosynthesis)
SCO1577 ArgD, Acetylornithine aminotransferase (ARG/PRO biosynthesis)
SCO5522 LeuB, 3-isopropylmalate dehydrogenase (VAL/LEU/ILEU biosynthesis)
B
48h 60h 48h 60h 48h 60h 48h 60h 48h 60h 48h 60h
-Pi +Pi -Pi +Pi -Pi +Pi
S. lividans pptA S. coelicolor
Color Key
-2 -1 0 1 2
Value

Figure S3B

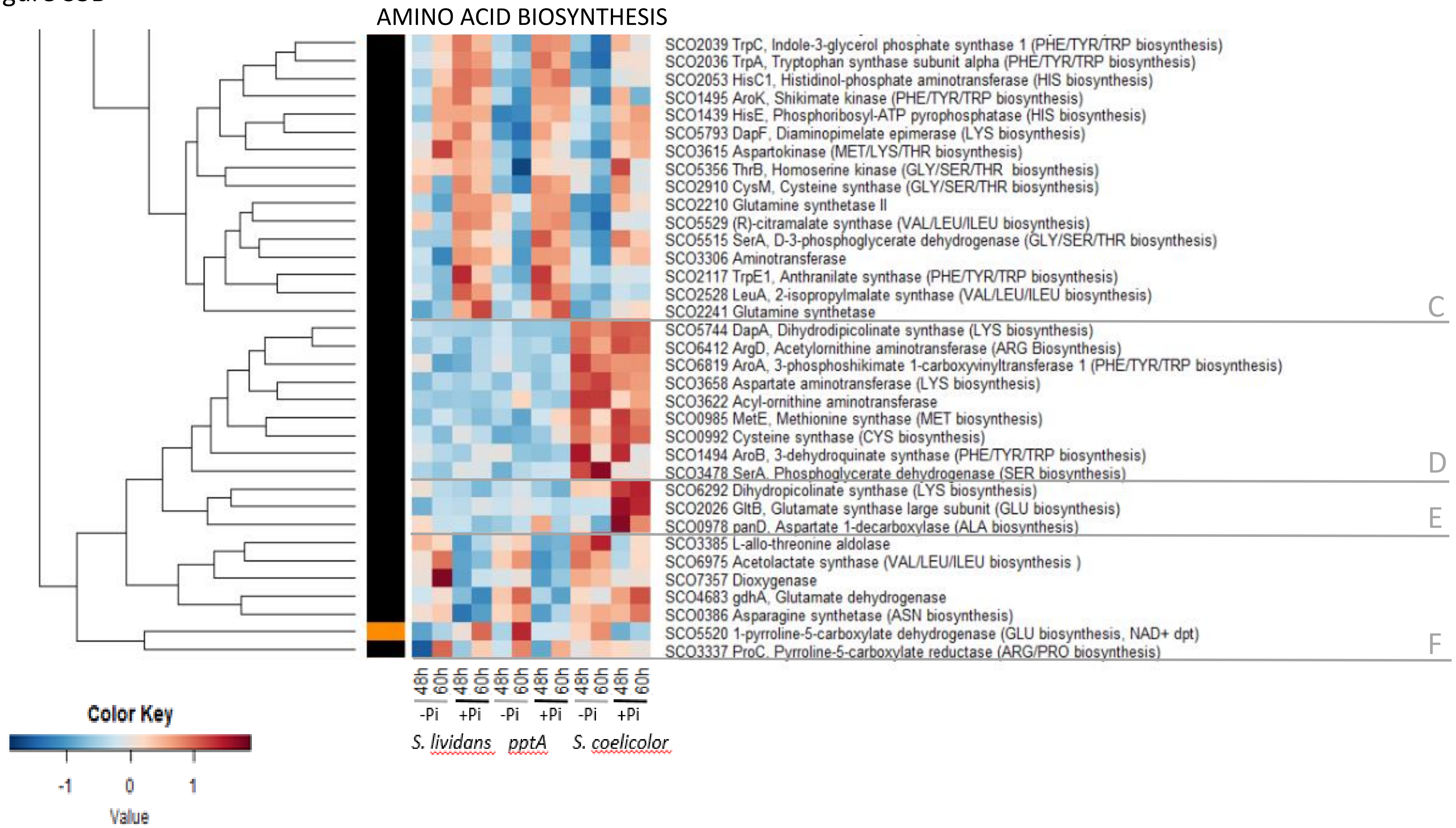
AMINO ACID BIOSYNTHESIS
SCO2039 TrpC, Indole-3-glycerol phosphate synthase 1 (PHE/TYR/TRP biosynthesis)
SCO2036 TrpA, Tryptophan synthase subunit alpha (PHE/TYR/TRP biosynthesis)
SCO2053 HisC1, Histidinol-phosphate aminotransferase (HIS biosynthesis)
SCO1495 AroK, Shikimate kinase (PHE/TYR/TRP biosynthesis)
SCO1439 HisE, Phosphoribosyl-ATP pyrophosphatase (HIS biosynthesis)
SCO5793 DapF, Diaminopimelate epimerase (LYS biosynthesis)
SCO3615 Aspartokinase (MET/LYS/THR biosynthesis)
SCO5356 ThrB, Homoserine kinase (GLY/SER/THR biosynthesis)
SCO2910 CysM, Cysteine synthase (GLY/SER/THR biosynthesis)
SCO2210 Glutamine synthetase II
SCO5529 (R)-citramalate synthase (VAL/LEU/ILEU biosynthesis)
SCO5515 SerA, D-3-phosphoglycerate dehydrogenase (GLY/SER/THR biosynthesis)
SCO3306 Aminotransferase
SCO2117 TrpE1, Anthranilate synthase (PHE/TYR/TRP biosynthesis)
SCO2528 LeuA, 2-isopropylmalate synthase (VAL/LEU/ILEU biosynthesis)
SCO2241 Glutamine synthetase
C
SCO5744 DapA, Dihydrodipicolinate synthase (LYS biosynthesis)
SCO6412 ArgD, Acetylornithine aminotransferase (ARG Biosynthesis)
SCO6819 AroA, 3-phosphoshikimate 1-carboxyvinyltransferase 1 (PHE/TYR/TRP biosynthesis)
SCO3658 Aspartate aminotransferase (LYS biosynthesis)
SCO3622 Acyl-ornithine aminotransferase
SCO0985 MetE, Methionine synthase (MET biosynthesis)
SCO0992 Cysteine synthase (CYS biosynthesis)
SCO1494 AroB, 3-dehydroquinate synthase (PHE/TYR/TRP biosynthesis)
SCO3478 SerA, Phosphoglycerate dehydrogenase (SER biosynthesis)
D
SCO6292 Dihydropicolinate synthase (LYS biosynthesis)
SCO2026 GltB, Glutamate synthase large subunit (GLU biosynthesis)
SCO0978 panD, Aspartate 1-decarboxylase (ALA biosynthesis)
E
SCO3385 L-allo-threonine aldolase
SCO6975 Acetolactate synthase (VAL/LEU/ILEU biosynthesis)
SCO7357 Dioxygenase
SCO4683 gdhA, Glutamate dehydrogenase
SCO0386 Asparagine synthetase (ASN biosynthesis)
SCO5520 1-pyrroline-5-carboxylate dehydrogenase (GLU biosynthesis, NAD+ dpt)
SCO3337 ProC, Pyrroline-5-carboxylate reductase (ARG/PRO biosynthesis)
F
48h 60h 48h 60h 48h 60h 48h 60h 48h 60h 48h 60h
-Pi +Pi -Pi +Pi -Pi +Pi
S. lividans pptA S. coelicolor
Color Key
-1 0 1
Value

Figure S4A

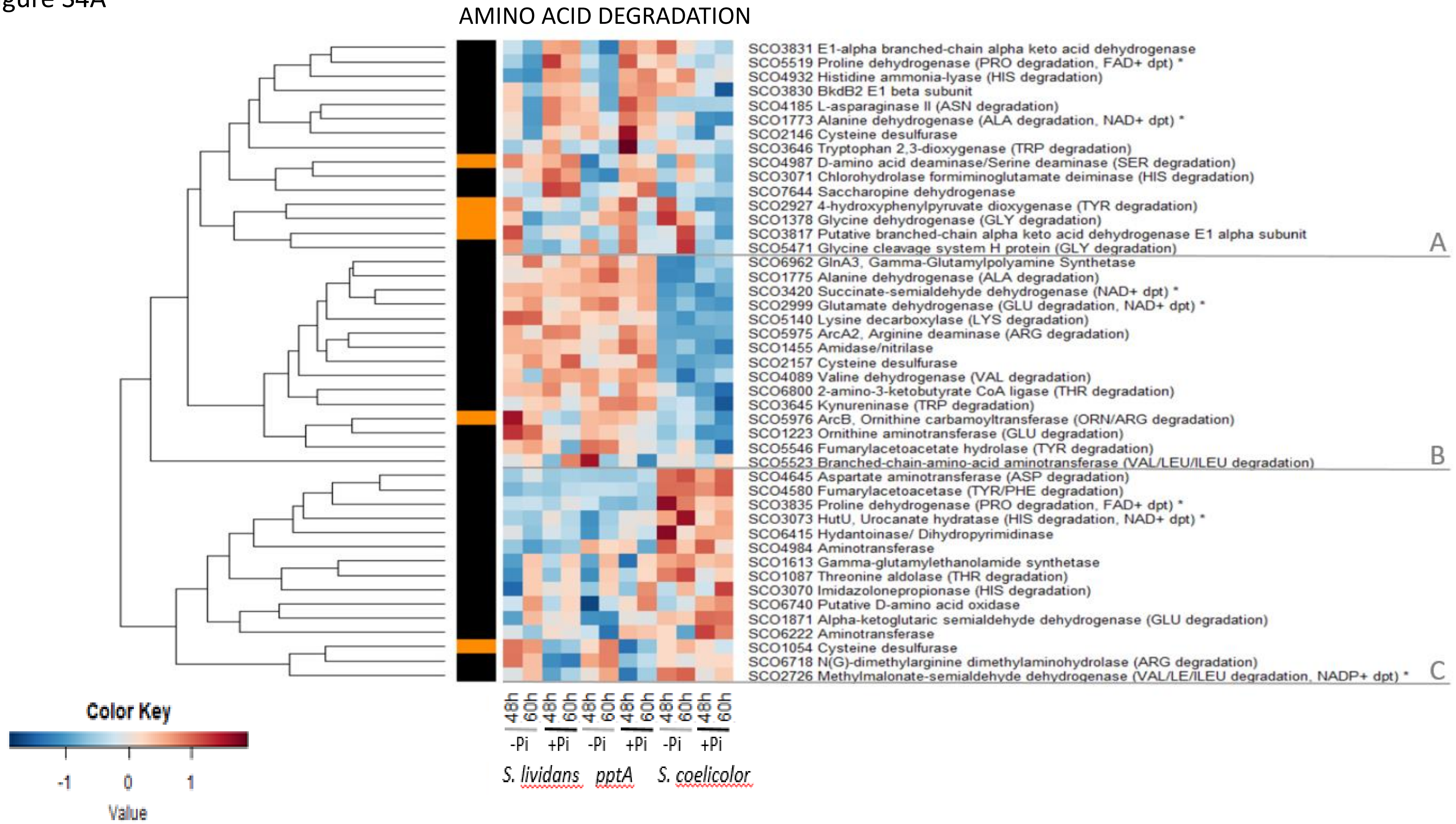
AMINO ACID DEGRADATION
SCO3831 E1-alpha branched-chain alpha keto acid dehydrogenase
SCO5519 Proline dehydrogenase (PRO degradation, FAD+ dpt) *
SCO4932 Histidine ammonia-lyase (HIS degradation)
SCO3830 BkdB2 E1 beta subunit
SCO4185 L-asparaginase II (ASN degradation)
SCO1773 Alanine dehydrogenase (ALA degradation, NAD+ dpt) *
SCO2146 Cysteine desulfurase
SCO3646 Tryptophan 2,3-dioxygenase (TRP degradation)
SCO4987 D-amino acid deaminase/Serine deaminase (SER degradation)
SCO3071 Chlorohydrolase formiminoglutamate deiminase (HIS degradation)
SCO7644 Saccharopine dehydrogenase
SCO2927 4-hydroxyphenylpyruvate dioxygenase (TYR degradation)
SCO1378 Glycine dehydrogenase (GLY degradation)
SCO3817 Putative branched-chain alpha keto acid dehydrogenase E1 alpha subunit
SCO5471 Glycine cleavage system H protein (GLY degradation)
A
SCO6962 GlnA3, Gamma-Glutamylpolyamine Synthetase
SCO1775 Alanine dehydrogenase (ALA degradation)
SCO3420 Succinate-semialdehyde dehydrogenase (NAD+ dpt) *
SCO2999 Glutamate dehydrogenase (GLU degradation, NAD+ dpt) *
SCO5140 Lysine decarboxylase (LYS degradation)
SCO5975 ArcA2, Arginine deaminase (ARG degradation)
SCO1455 Amidase/nitrilase
SCO2157 Cysteine desulfurase
SCO4089 Valine dehydrogenase (VAL degradation)
SCO6800 2-amino-3-ketobutyrate CoA ligase (THR degradation)
SCO3645 Kynureninase (TRP degradation)
SCO5976 ArcB, Ornithine carbamoyltransferase (ORN/ARG degradation)
SCO1223 Ornithine aminotransferase (GLU degradation)
SCO5546 Fumarylacetoacetate hydrolase (TYR degradation)
SCO5523 Branched-chain-amino-acid aminotransferase (VAL/LEU/ILEU degradation)
B
SCO4645 Aspartate aminotransferase (ASP degradation)
SCO4580 Fumarylacetoacetase (TYR/PHE degradation)
SCO3835 Proline dehydrogenase (PRO degradation, FAD+ dpt) *
SCO3073 HutU, Urocanate hydratase (HIS degradation, NAD+ dpt) *
SCO6415 Hydantoinase/ Dihydropyrimidinase
SCO4984 Aminotransferase
SCO1613 Gamma-glutamylethanolamide synthetase
SCO1087 Threonine aldolase (THR degradation)
SCO3070 Imidazolonepropionase (HIS degradation)
SCO6740 Putative D-amino acid oxidase
SCO1871 Alpha-ketoglutaric semialdehyde dehydrogenase (GLU degradation)
SCO6222 Aminotransferase
SCO1054 Cysteine desulfurase
SCO6718 N(G)-dimethylarginine dimethylaminohydrolase (ARG degradation)
SCO2726 Methylmalonate-semialdehyde dehydrogenase (VAL/LE/ILEU degradation, NADP+ dpt) *
C
48h 60h 48h 60h 48h 60h 48h 60h 48h 60h 48h 60h
-Pi +Pi -Pi +Pi -Pi +Pi
S. lividans pptA S. coelicolor
Color Key
-1 0 1
Value

Figure S4B

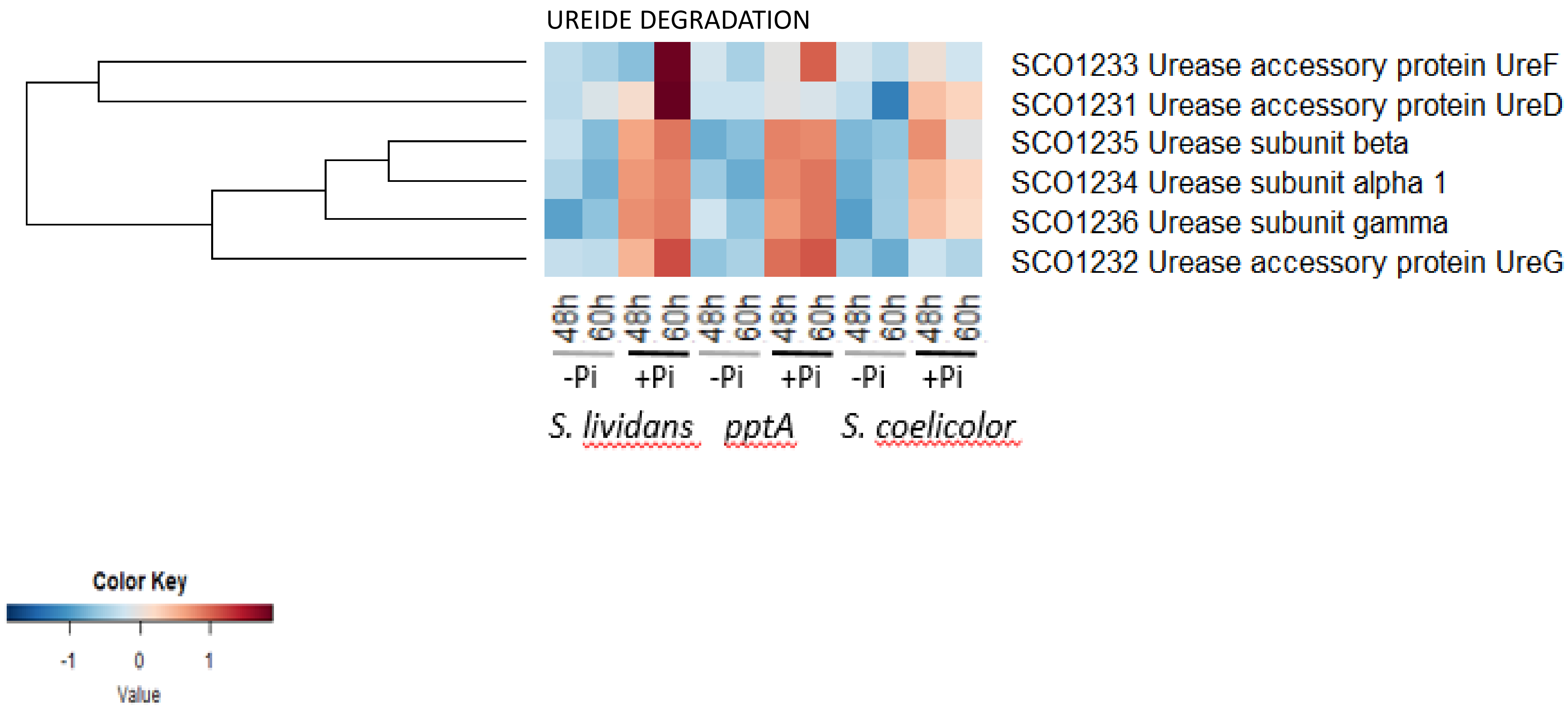
UREIDE DEGRADATION
SCO1233 Urease accessory protein UreF
SCO1231 Urease accessory protein UreD
SCO1235 Urease subunit beta
SCO1234 Urease subunit alpha 1
SCO1236 Urease subunit gamma
SCO1232 Urease accessory protein UreG
48h
60h
-Pi
+Pi
S. lividans
pptA
S. coelicolor
Color Key
-1
0
1
Value

Figure S5A

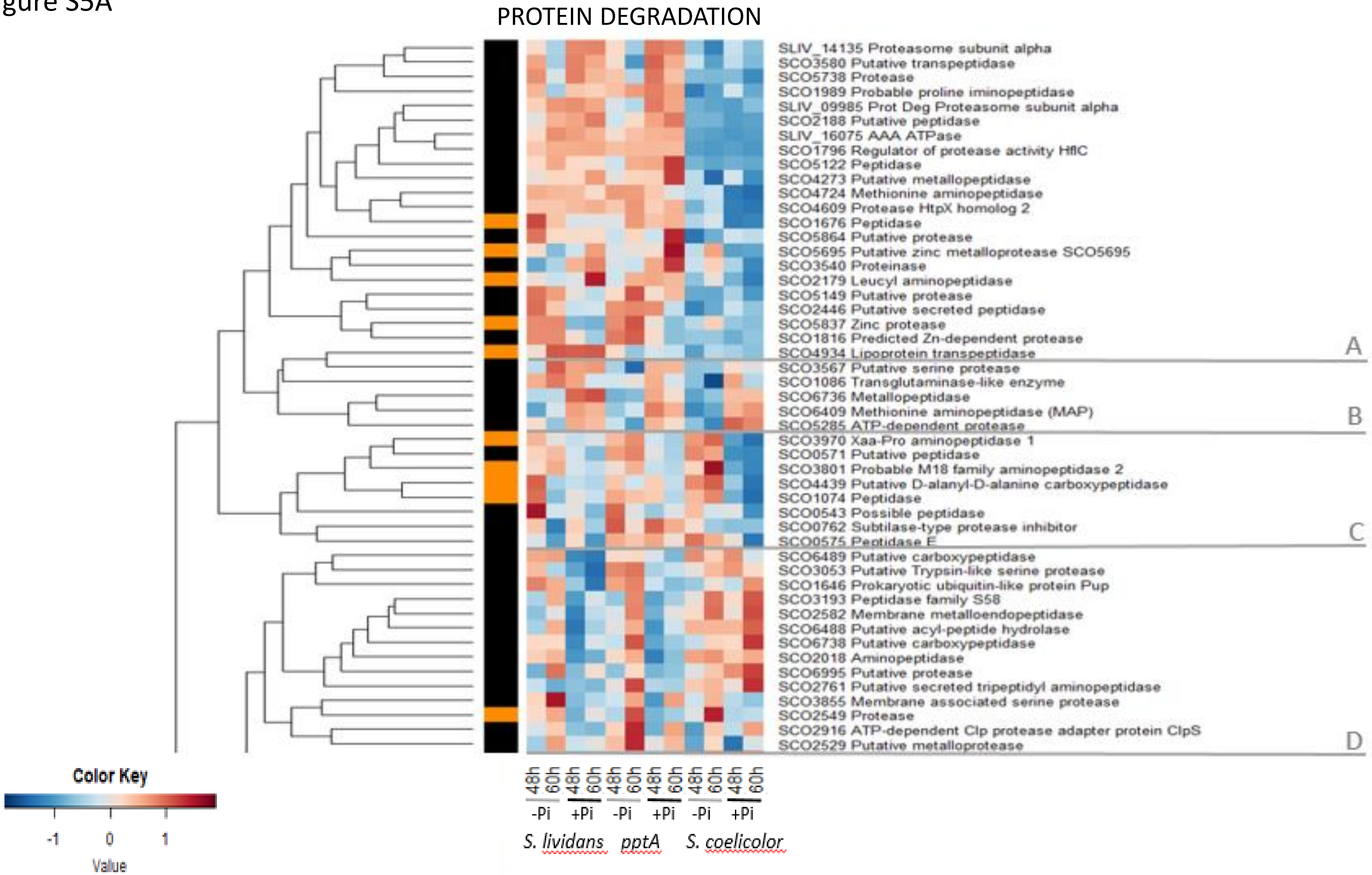
PROTEIN DEGRADATION
SLIV_14135 Proteasome subunit alpha
SCO3580 Putative transpeptidase
SCO5738 Protease
SCO1989 Probable proline iminopeptidase
SLIV_09985 Prot Deg Proteasome subunit alpha
SCO2188 Putative peptidase
SLIV_16075 AAA ATPase
SCO1796 Regulator of protease activity HflC
SCO5122 Peptidase
SCO4273 Putative metallopeptidase
SCO4724 Methionine aminopeptidase
SCO4609 Protease HtpX homolog 2
SCO1676 Peptidase
SCO5864 Putative protease
SCO5695 Putative zinc metalloprotease SCO5695
SCO3540 Proteinase
SCO2179 Leucyl aminopeptidase
SCO5149 Putative protease
SCO2446 Putative secreted peptidase
SCO5837 Zinc protease
SCO1816 Predicted Zn-dependent protease
SCO4934 Lipoprotein transpeptidase
A
SCO3567 Putative serine protease
SCO1086 Transglutaminase-like enzyme
SCO6736 Metallopeptidase
SCO6409 Methionine aminopeptidase (MAP)
SCO5285 ATP-dependent protease
B
SCO3970 Xaa-Pro aminopeptidase 1
SCO0571 Putative peptidase
SCO3801 Probable M18 family aminopeptidase 2
SCO4439 Putative D-alanyl-D-alanine carboxypeptidase
SCO1074 Peptidase
SCO0543 Possible peptidase
SCO0762 Subtilase-type protease inhibitor
SCO0575 Peptidase E
C
SCO6489 Putative carboxypeptidase
SCO3053 Putative Trypsin-like serine protease
SCO1646 Prokaryotic ubiquitin-like protein Pup
SCO3193 Peptidase family S58
SCO2582 Membrane metalloendopeptidase
SCO6488 Putative acyl-peptide hydrolase
SCO6738 Putative carboxypeptidase
SCO2018 Aminopeptidase
SCO6995 Putative protease
SCO2761 Putative secreted tripeptidyl aminopeptidase
SCO3855 Membrane associated serine protease
SCO2549 Protease
SCO2916 ATP-dependent Clp protease adapter protein ClpS
SCO2529 Putative metalloprotease
D
48h 60h 48h 60h 48h 60h 48h 60h 48h 60h 48h 60h
-Pi +Pi -Pi +Pi -Pi +Pi
S. lividans pptA S. coelicolor
Color Key
-1 0 1
Value

Figure S5B

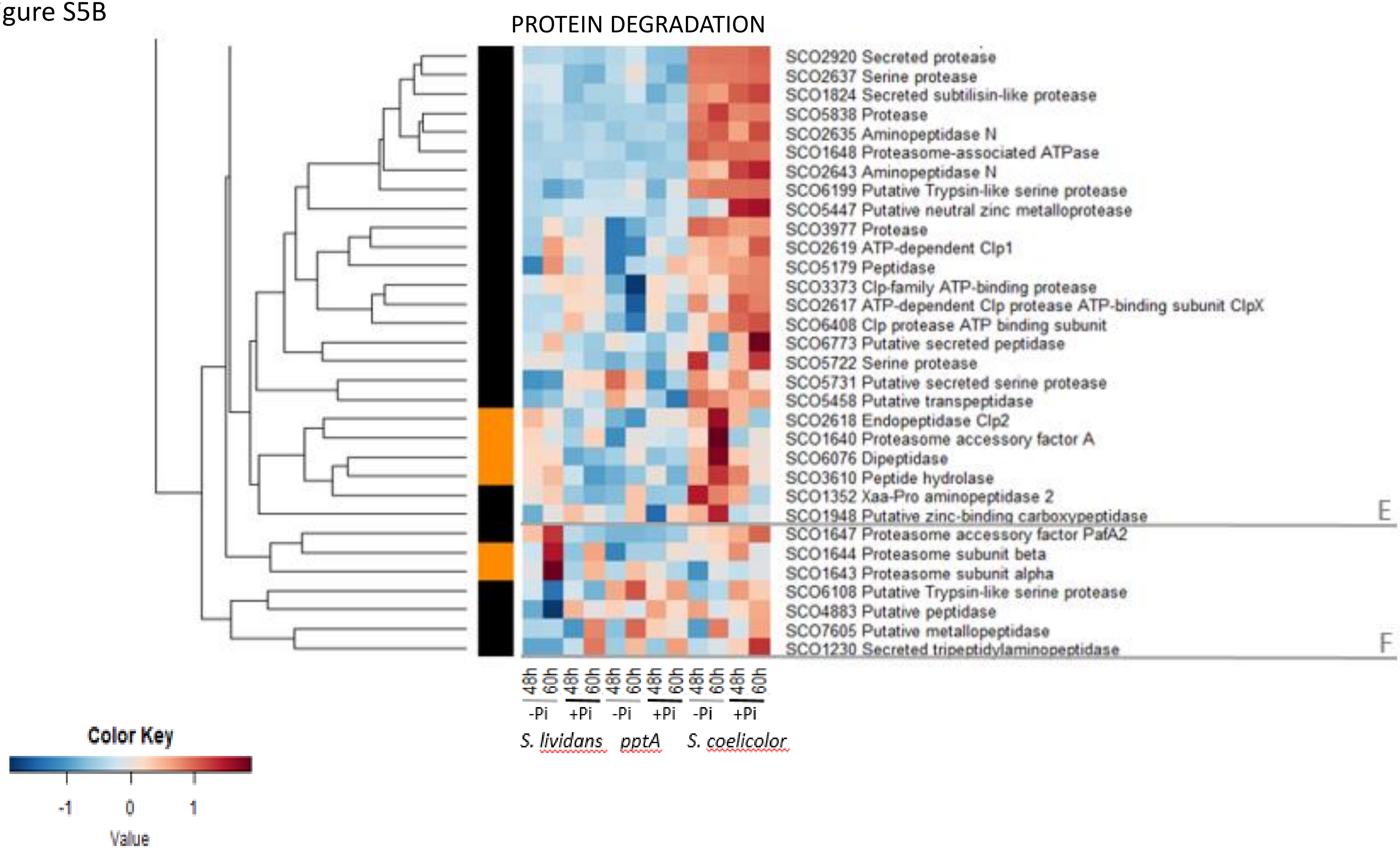
PROTEIN DEGRADATION
SCO2920 Secreted protease
SCO2637 Serine protease
SCO1824 Secreted subtilisin-like protease
SCO5838 Protease
SCO2635 Aminopeptidase N
SCO1648 Proteasome-associated ATPase
SCO2643 Aminopeptidase N
SCO6199 Putative Trypsin-like serine protease
SCO5447 Putative neutral zinc metalloprotease
SCO3977 Protease
SCO2619 ATP-dependent Clp1
SCO5179 Peptidase
SCO3373 Clp-family ATP-binding protease
SCO2617 ATP-dependent Clp protease ATP-binding subunit ClpX
SCO6408 Clp protease ATP binding subunit
SCO6773 Putative secreted peptidase
SCO5722 Serine protease
SCO5731 Putative secreted serine protease
SCO5458 Putative transpeptidase
SCO2618 Endopeptidase Clp2
SCO1640 Proteasome accessory factor A
SCO6076 Dipeptidase
SCO3610 Peptide hydrolase
SCO1352 Xaa-Pro aminopeptidase 2
SCO1948 Putative zinc-binding carboxypeptidase
E
SCO1647 Proteasome accessory factor PafA2
SCO1644 Proteasome subunit beta
SCO1643 Proteasome subunit alpha
SCO6108 Putative Trypsin-like serine protease
SCO4883 Putative peptidase
SCO7605 Putative metallopeptidase
SCO1230 Secreted tripeptidylaminopeptidase
F
48h 60h 48h 60h 48h 60h 48h 60h 48h 60h 48h 60h
-Pi +Pi -Pi +Pi -Pi +Pi
S. lividans pptA S. coelicolor
Color Key
-1 0 1
Value

Figure S6

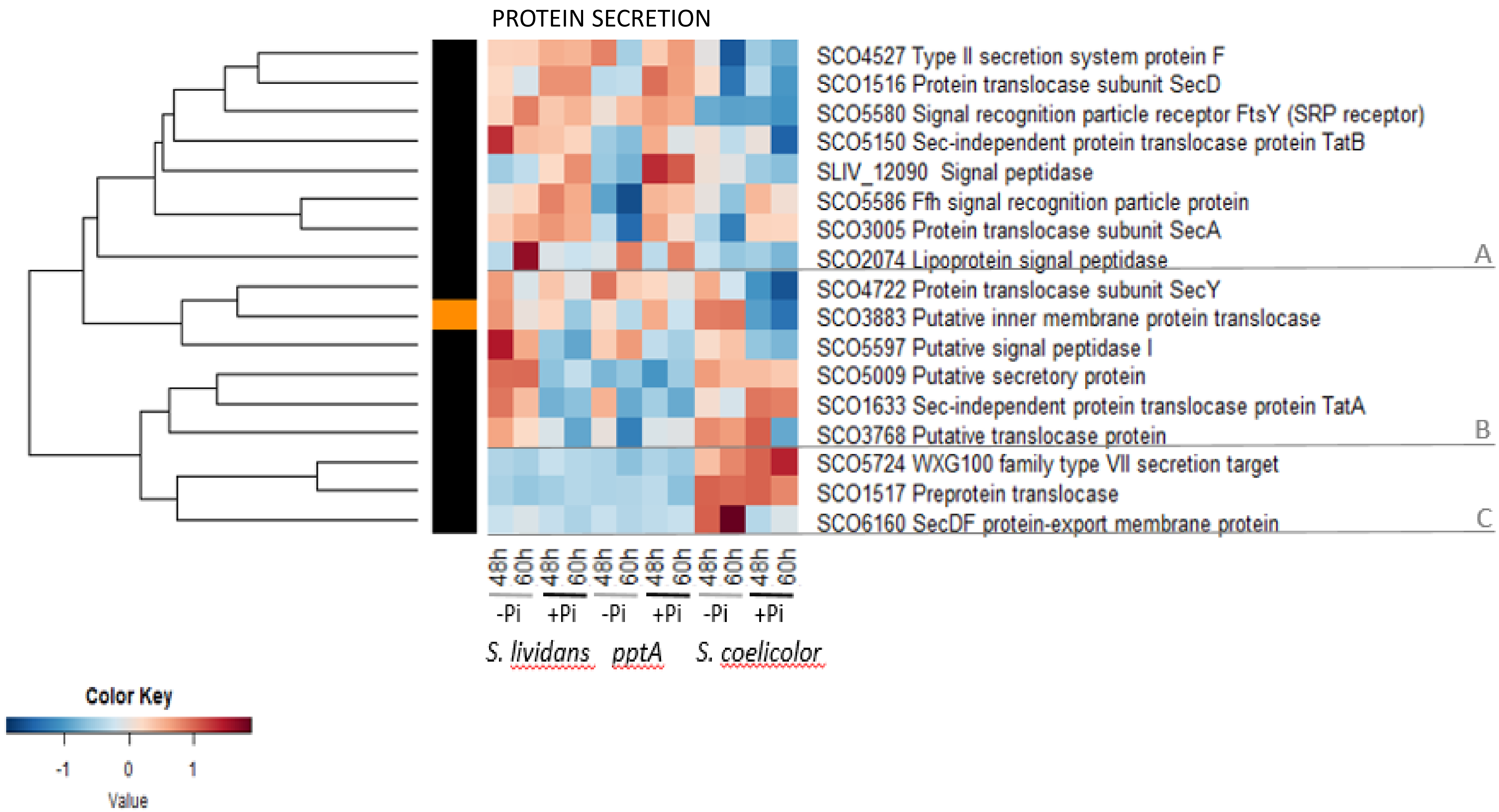
PROTEIN SECRETION
SCO4527 Type II secretion system protein F
SCO1516 Protein translocase subunit SecD
SCO5580 Signal recognition particle receptor FtsY (SRP receptor)
SCO5150 Sec-independent protein translocase protein TatB
SLIV_12090 Signal peptidase
SCO5586 Ffh signal recognition particle protein
SCO3005 Protein translocase subunit SecA
SCO2074 Lipoprotein signal peptidase
A
SCO4722 Protein translocase subunit SecY
SCO3883 Putative inner membrane protein translocase
SCO5597 Putative signal peptidase I
SCO5009 Putative secretory protein
SCO1633 Sec-independent protein translocase protein TatA
SCO3768 Putative translocase protein
B
SCO5724 WXG100 family type VII secretion target
SCO1517 Preprotein translocase
SCO6160 SecDF protein-export membrane protein
C
48h 60h 48h 60h 48h 60h 48h 60h 48h 60h 48h 60h
-Pi +Pi -Pi +Pi -Pi +Pi
S. lividans pptA S. coelicolor
Color Key
-1 0 1
Value

Figure S7A

NUCLEOTIDE BIOSYNTHESIS

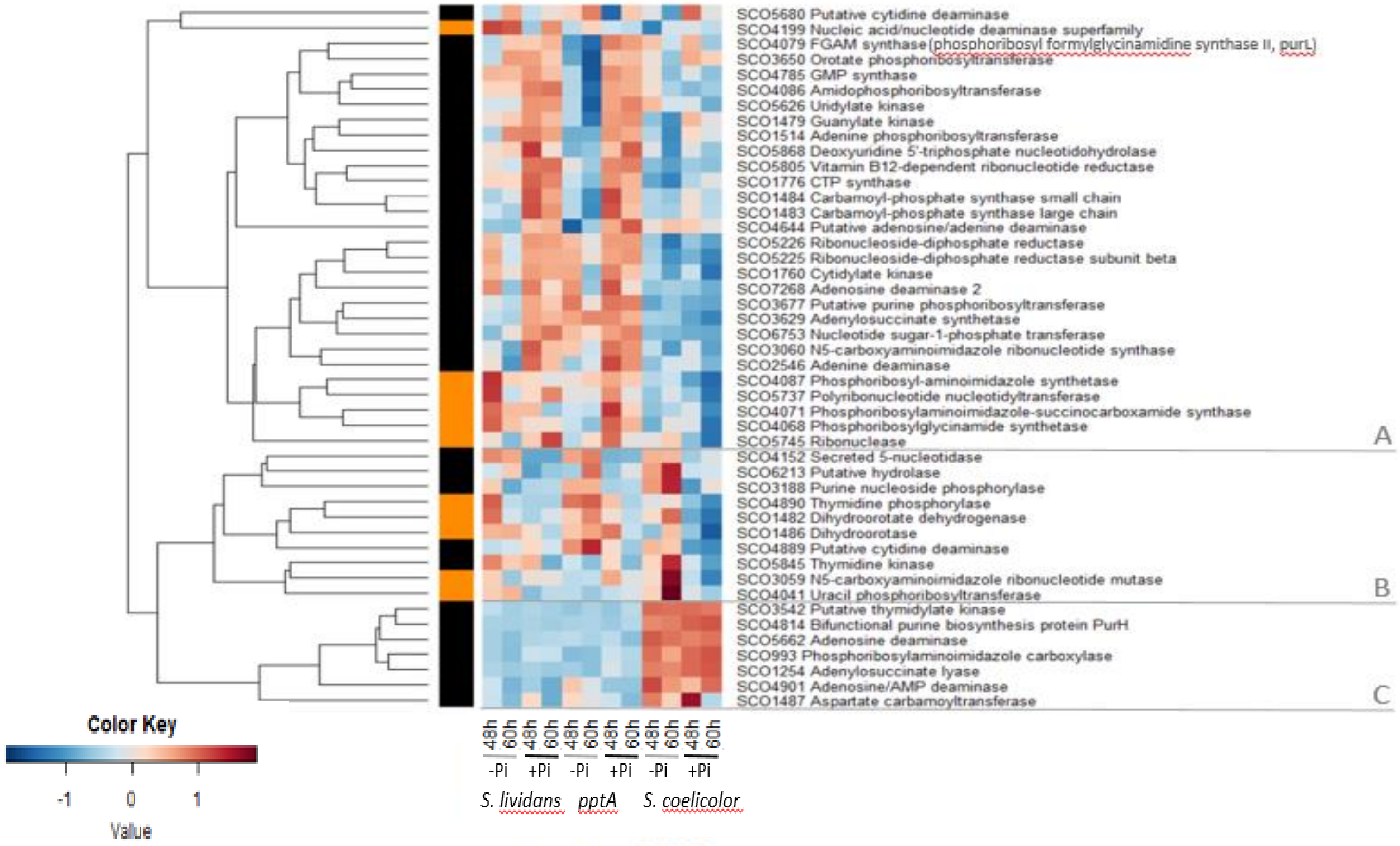

Figure S7B

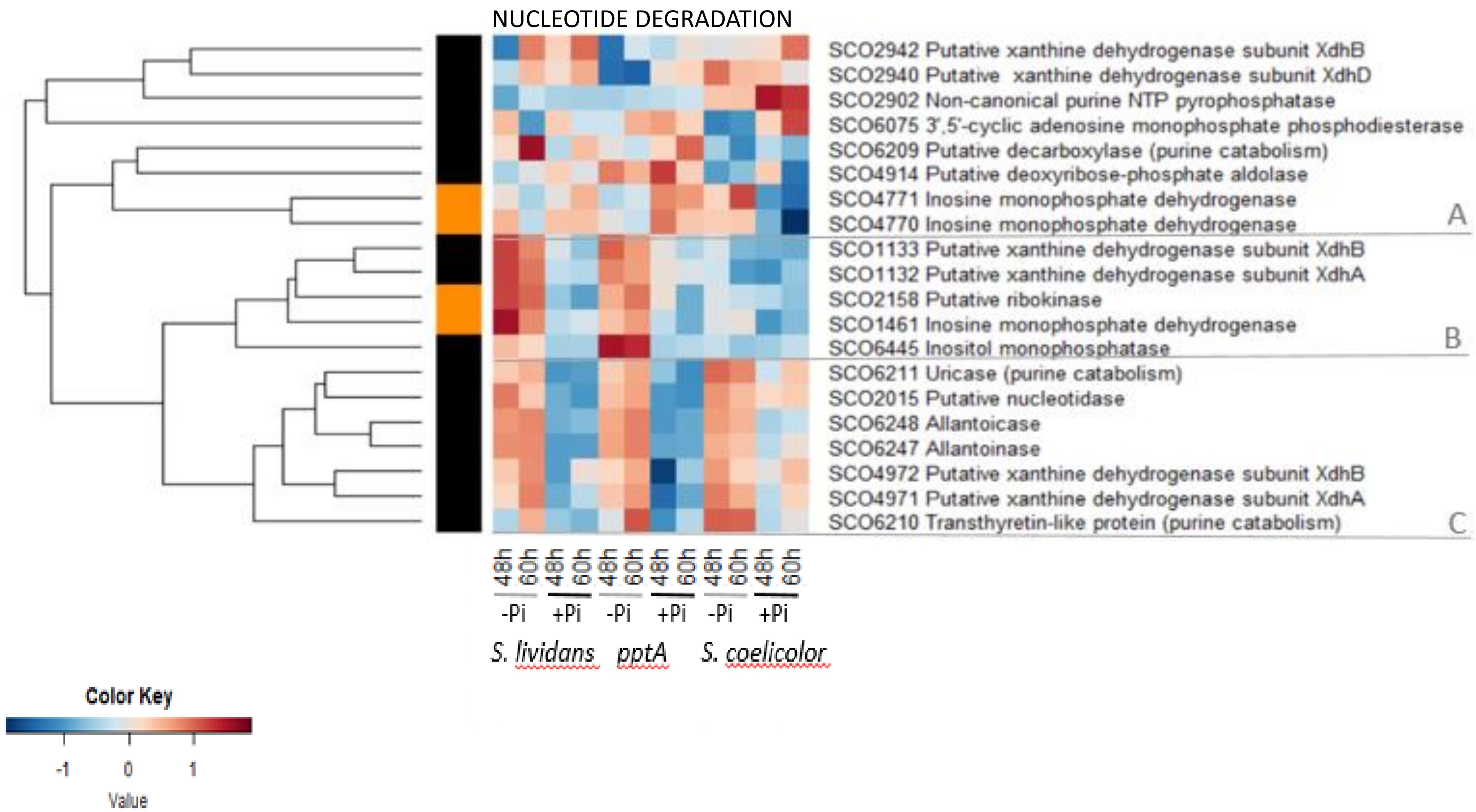

NUCLEOTIDE DEGRADATION
SCO2942 Putative xanthine dehydrogenase subunit XdhB
SCO2940 Putative xanthine dehydrogenase subunit XdhD
SCO2902 Non-canonical purine NTP pyrophosphatase
SCO6075 3',5'-cyclic adenosine monophosphate phosphodiesterase
SCO6209 Putative decarboxylase (purine catabolism)
SCO4914 Putative deoxyribose-phosphate aldolase
SCO4771 Inosine monophosphate dehydrogenase
SCO4770 Inosine monophosphate dehydrogenase
A
SCO1133 Putative xanthine dehydrogenase subunit XdhB
SCO1132 Putative xanthine dehydrogenase subunit XdhA
SCO2158 Putative ribokinase
SCO1461 Inosine monophosphate dehydrogenase
SCO6445 Inositol monophosphatase
B
SCO6211 Uricase (purine catabolism)
SCO2015 Putative nucleotidase
SCO6248 Allantoicase
SCO6247 Allantoinase
SCO4972 Putative xanthine dehydrogenase subunit XdhB
SCO4971 Putative xanthine dehydrogenase subunit XdhA
SCO6210 Transthyretin-like protein (purine catabolism)
C
48h 60h 48h 60h 48h 60h 48h 60h 48h 60h 48h 60h
-Pi +Pi -Pi +Pi -Pi +Pi
S. lividans pptA S. coelicolor
Color Key
-1 0 1
Value

Figure S8

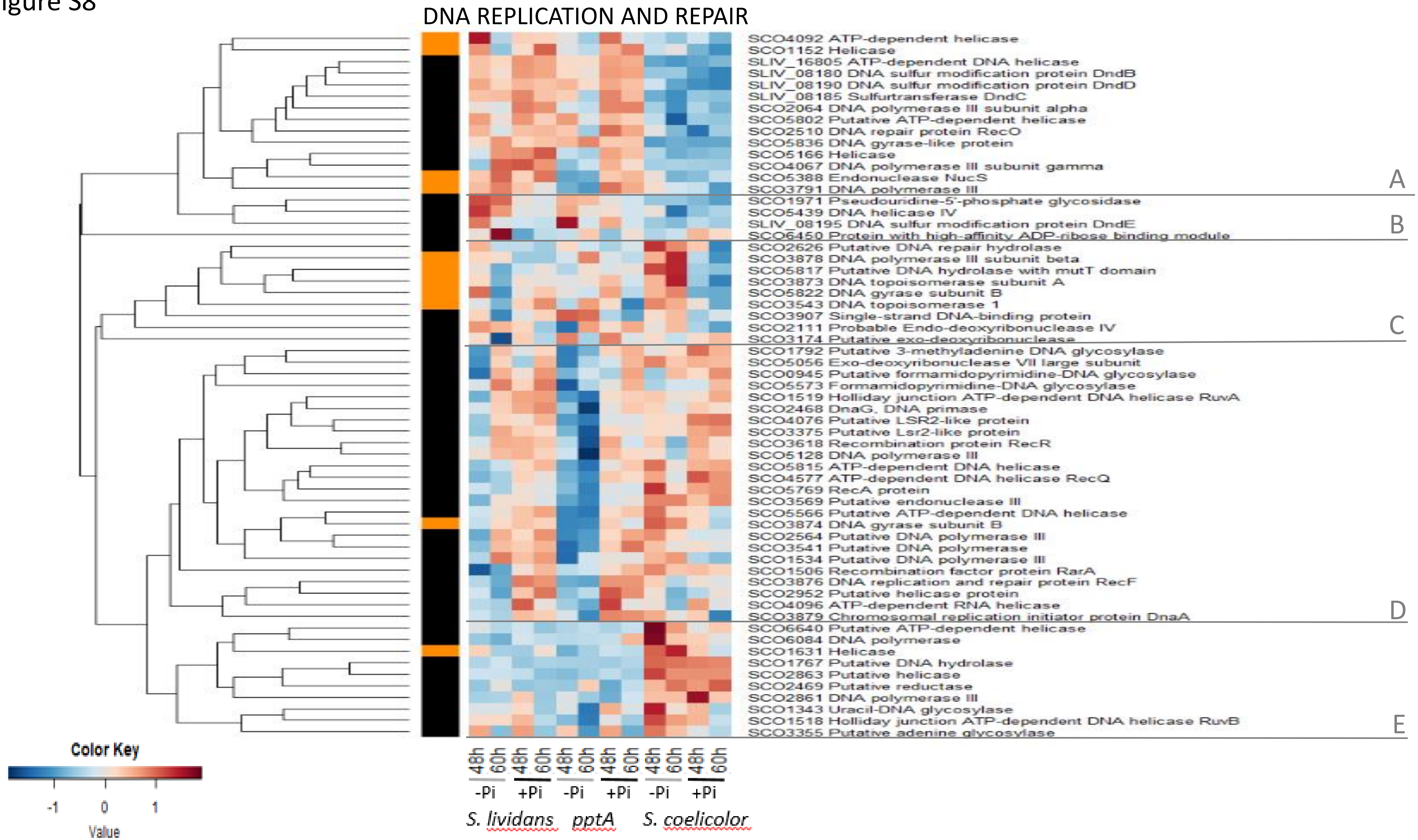
DNA REPLICATION AND REPAIR
SCO4092 ATP-dependent helicase
SCO1152 Helicase
SLIV_16805 ATP-dependent DNA helicase
SLIV_08180 DNA sulfur modification protein DndB
SLIV_08190 DNA sulfur modification protein DndD
SLIV_08185 Sulfurtransferase DndC
SCO2064 DNA polymerase III subunit alpha
SCO5802 Putative ATP-dependent helicase
SCO2510 DNA repair protein RecO
SCO5836 DNA gyrase-like protein
SCO5166 Helicase
SCO4067 DNA polymerase III subunit gamma
SCO5388 Endonuclease NucS
SCO3791 DNA polymerase III
A
SCO1971 Pseudouridine-5'-phosphate glycosidase
SCO5439 DNA helicase IV
SLIV_08195 DNA sulfur modification protein DndE
SCO6450 Protein with high-affinity ADP-ribose binding module
B
SCO2626 Putative DNA repair hydrolase
SCO3878 DNA polymerase III subunit beta
SCO5817 Putative DNA hydrolase with mutT domain
SCO3873 DNA topoisomerase subunit A
SCO5822 DNA gyrase subunit B
SCO3543 DNA topoisomerase 1
SCO3907 Single-strand DNA-binding protein
SCO2111 Probable Endo-deoxyribonuclease IV
SCO3174 Putative exo-deoxyribonuclease
C
SCO1792 Putative 3-methyladenine DNA glycosylase
SCO5056 Exo-deoxyribonuclease VII large subunit
SCO0945 Putative formamidopyrimidine-DNA glycosylase
SCO5573 Formamidopyrimidine-DNA glycosylase
SCO1519 Holliday junction ATP-dependent DNA helicase RuvA
SCO2468 DnaG, DNA primase
SCO4076 Putative LSR2-like protein
SCO3375 Putative Lsr2-like protein
SCO3618 Recombination protein RecR
SCO5128 DNA polymerase III
SCO5815 ATP-dependent DNA helicase
SCO4577 ATP-dependent DNA helicase RecQ
SCO5769 RecA protein
SCO3569 Putative endonuclease III
SCO5566 Putative ATP-dependent DNA helicase
SCO3874 DNA gyrase subunit B
SCO2564 Putative DNA polymerase III
SCO3541 Putative DNA polymerase
SCO1534 Putative DNA polymerase III
SCO1506 Recombination factor protein RarA
SCO3876 DNA replication and repair protein RecF
SCO2952 Putative helicase protein
SCO4096 ATP-dependent RNA helicase
SCO3879 Chromosomal replication initiator protein DnaA
D
SCO6640 Putative ATP-dependent helicase
SCO6084 DNA polymerase
SCO1631 Helicase
SCO1767 Putative DNA hydrolase
SCO2863 Putative helicase
SCO2469 Putative reductase
SCO2861 DNA polymerase III
SCO1343 Uracil-DNA glycosylase
SCO1518 Holliday junction ATP-dependent DNA helicase RuvB
SCO3355 Putative adenine glycosylase
E
Color Key
-1
0
1
Value
48h 60h 48h 60h 48h 60h 48h 60h 48h 60h 48h 60h
-Pi +Pi -Pi +Pi -Pi +Pi
S. lividans pptA S. coelicolor

Figure S9

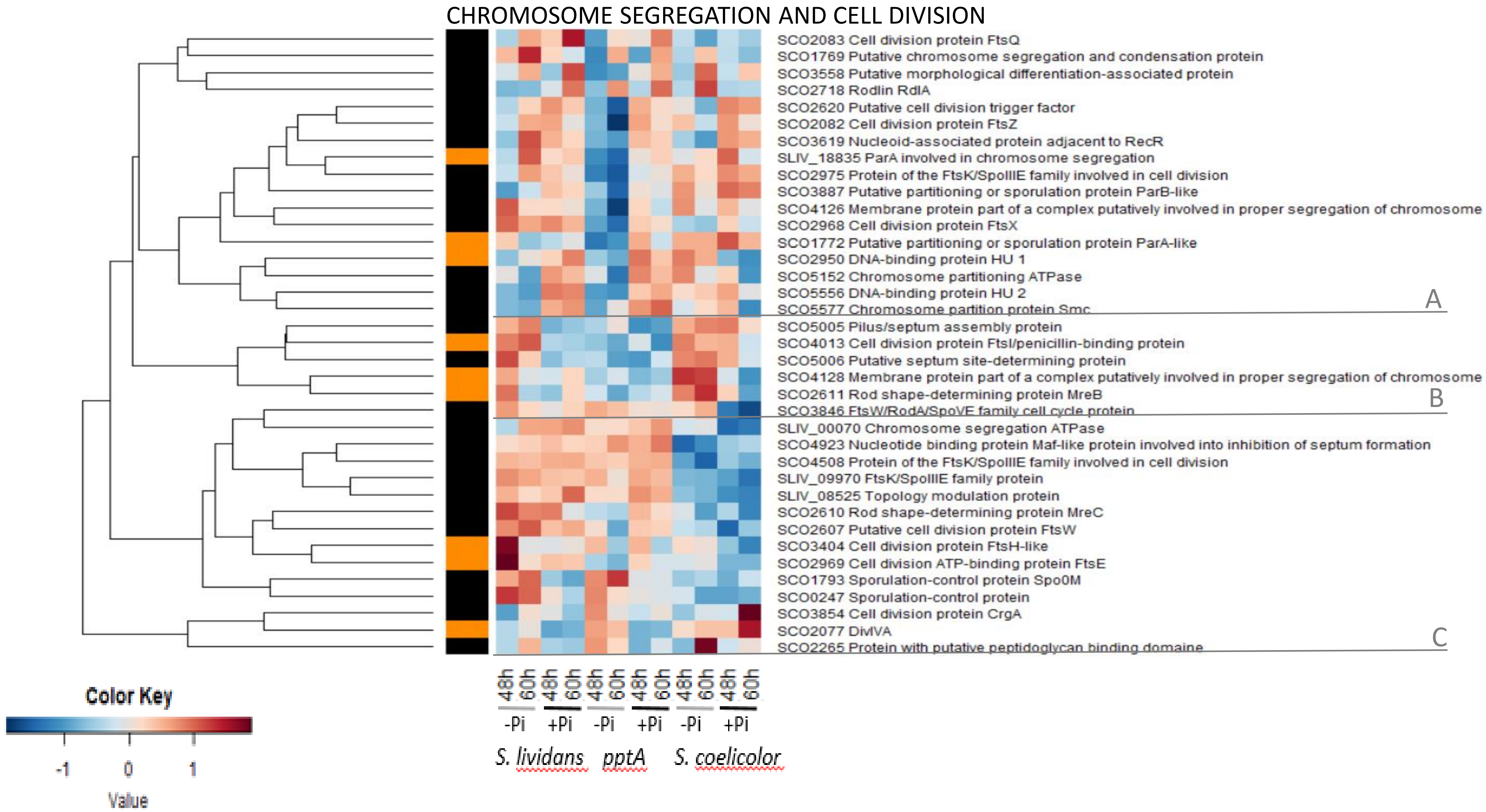
CHROMOSOME SEGREGATION AND CELL DIVISION
SCO2083 Cell division protein FtsQ
SCO1769 Putative chromosome segregation and condensation protein
SCO3558 Putative morphological differentiation-associated protein
SCO2718 Rodlin RdlA
SCO2620 Putative cell division trigger factor
SCO2082 Cell division protein FtsZ
SCO3619 Nucleoid-associated protein adjacent to RecR
SLIV_18835 ParA involved in chromosome segregation
SCO2975 Protein of the FtsK/SpoIIIE family involved in cell division
SCO3887 Putative partitioning or sporulation protein ParB-like
SCO4126 Membrane protein part of a complex putatively involved in proper segregation of chromosome
SCO2968 Cell division protein FtsX
SCO1772 Putative partitioning or sporulation protein ParA-like
SCO2950 DNA-binding protein HU 1
SCO5152 Chromosome partitioning ATPase
SCO5556 DNA-binding protein HU 2
SCO5577 Chromosome partition protein Smc
A
SCO5005 Pilus/septum assembly protein
SCO4013 Cell division protein FtsI/penicillin-binding protein
SCO5006 Putative septum site-determining protein
SCO4128 Membrane protein part of a complex putatively involved in proper segregation of chromosome
SCO2611 Rod shape-determining protein MreB
SCO3846 FtsW/RodA/SpoVE family cell cycle protein
B
SLIV_00070 Chromosome segregation ATPase
SCO4923 Nucleotide binding protein Maf-like protein involved into inhibition of septum formation
SCO4508 Protein of the FtsK/SpoIIIE family involved in cell division
SLIV_09970 FtsK/SpoIIIE family protein
SLIV_08525 Topology modulation protein
SCO2610 Rod shape-determining protein MreC
SCO2607 Putative cell division protein FtsW
SCO3404 Cell division protein FtsH-like
SCO2969 Cell division ATP-binding protein FtsE
SCO1793 Sporulation-control protein Spo0M
SCO0247 Sporulation-control protein
SCO3854 Cell division protein CrgA
SCO2077 DivIVA
SCO2265 Protein with putative peptidoglycan binding domaine
C
48h 60h 48h 60h 48h 60h 48h 60h 48h 60h 48h 60h
-Pi +Pi -Pi +Pi -Pi +Pi
S. lividans pptA S. coelicolor
Color Key
-1 0 1
Value

Figure S10

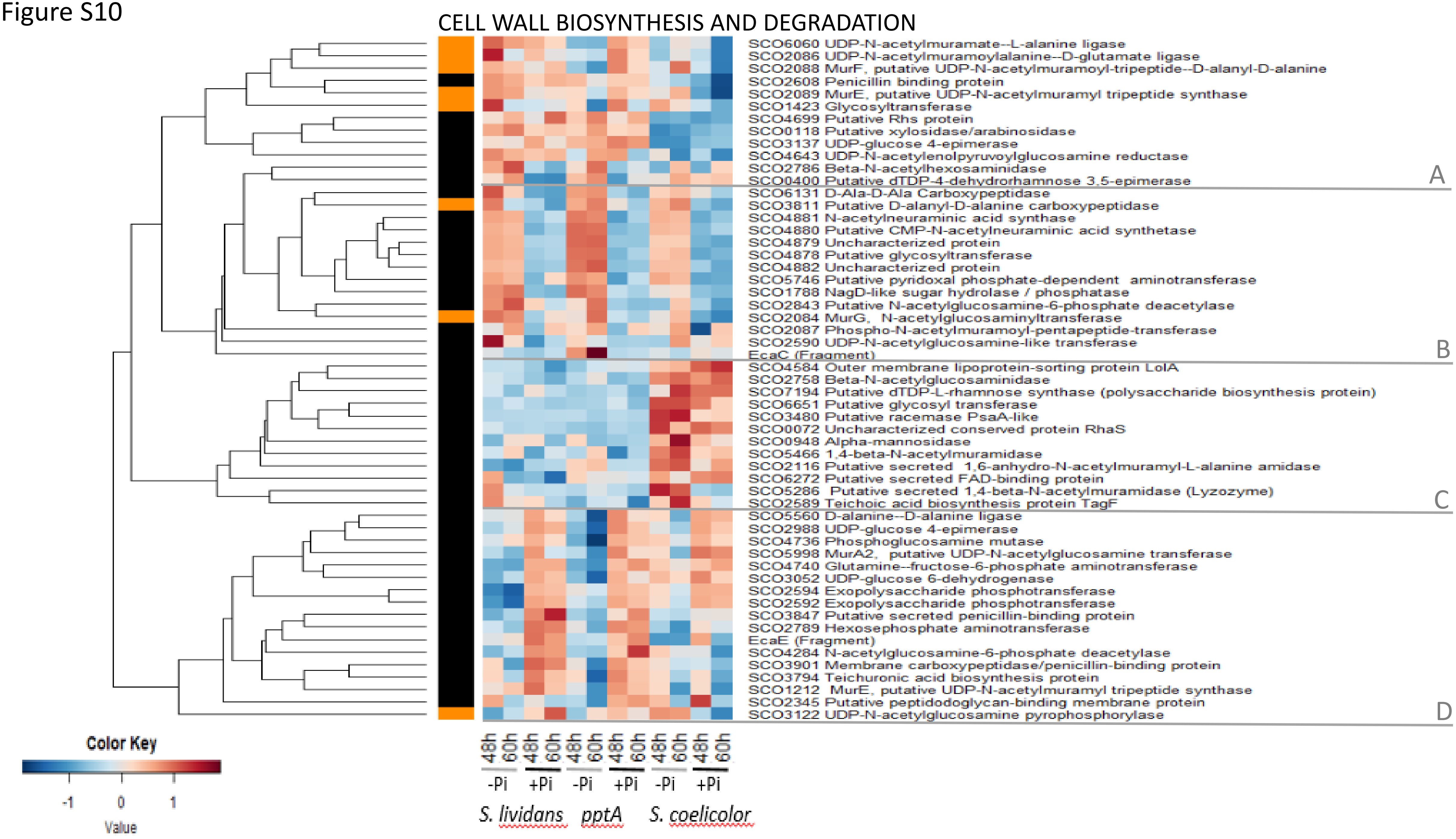
CELL WALL BIOSYNTHESIS AND DEGRADATION
SCO6060 UDP-N-acetylmuramate--L-alanine ligase
SCO2086 UDP-N-acetylmuramoylalanine--D-glutamate ligase
SCO2088 MurF, putative UDP-N-acetylmuramoyl-tripeptide--D-alanyl-D-alanine
SCO2608 Penicillin binding protein
SCO2089 MurE, putative UDP-N-acetylmuramyl tripeptide synthase
SCO1423 Glycosyltransferase
SCO4699 Putative Rhs protein
SCO0118 Putative xylosidase/arabinosidase
SCO3137 UDP-glucose 4-epimerase
SCO4643 UDP-N-acetylenolpyruvoylglucosamine reductase
SCO2786 Beta-N-acetylhexosaminidase
SCO0400 Putative dTDP-4-dehydrorhamnose 3,5-epimerase
A
SCO6131 D-Ala-D-Ala Carboxypeptidase
SCO3811 Putative D-alanyl-D-alanine carboxypeptidase
SCO4881 N-acetylneuraminic acid synthase
SCO4880 Putative CMP-N-acetylneuraminic acid synthetase
SCO4879 Uncharacterized protein
SCO4878 Putative glycosyltransferase
SCO4882 Uncharacterized protein
SCO5746 Putative pyridoxal phosphate-dependent aminotransferase
SCO1788 NagD-like sugar hydrolase / phosphatase
SCO2843 Putative N-acetylglucosamine-6-phosphate deacetylase
SCO2084 MurG, N-acetylglucosaminyltransferase
SCO2087 Phospho-N-acetylmuramoyl-pentapeptide-transferase
SCO2590 UDP-N-acetylglucosamine-like transferase
EcaC (Fragment)
B
SCO4584 Outer membrane lipoprotein-sorting protein LolA
SCO2758 Beta-N-acetylglucosaminidase
SCO7194 Putative dTDP-L-rhamnose synthase (polysaccharide biosynthesis protein)
SCO6651 Putative glycosyl transferase
SCO3480 Putative racemase PsaA-like
SCO0072 Uncharacterized conserved protein RhaS
SCO0948 Alpha-mannosidase
SCO5466 1,4-beta-N-acetylmuramidase
SCO2116 Putative secreted 1,6-anhydro-N-acetylmuramyl-L-alanine amidase
SCO6272 Putative secreted FAD-binding protein
SCO5286 Putative secreted 1,4-beta-N-acetylmuramidase (Lyzozyme)
SCO2589 Teichoic acid biosynthesis protein TagF
C
SCO5560 D-alanine--D-alanine ligase
SCO2988 UDP-glucose 4-epimerase
SCO4736 Phosphoglucosamine mutase
SCO5998 MurA2, putative UDP-N-acetylglucosamine transferase
SCO4740 Glutamine--fructose-6-phosphate aminotransferase
SCO3052 UDP-glucose 6-dehydrogenase
SCO2594 Exopolysaccharide phosphotransferase
SCO2592 Exopolysaccharide phosphotransferase
SCO3847 Putative secreted penicillin-binding protein
SCO2789 Hexosephosphate aminotransferase
EcaE (Fragment)
SCO4284 N-acetylglucosamine-6-phosphate deacetylase
SCO3901 Membrane carboxypeptidase/penicillin-binding protein
SCO3794 Teichuronic acid biosynthesis protein
SCO1212 MurE, putative UDP-N-acetylmuramyl tripeptide synthase
SCO2345 Putative peptidodoglycan-binding membrane protein
SCO3122 UDP-N-acetylglucosamine pyrophosphorylase
D
48h 60h 48h 60h 48h 60h 48h 60h 48h 60h 48h 60h
-Pi +Pi -Pi +Pi -Pi +Pi
S. lividans pptA S. coelicolor
Color Key
-1 0 1
Value

Figure S11A

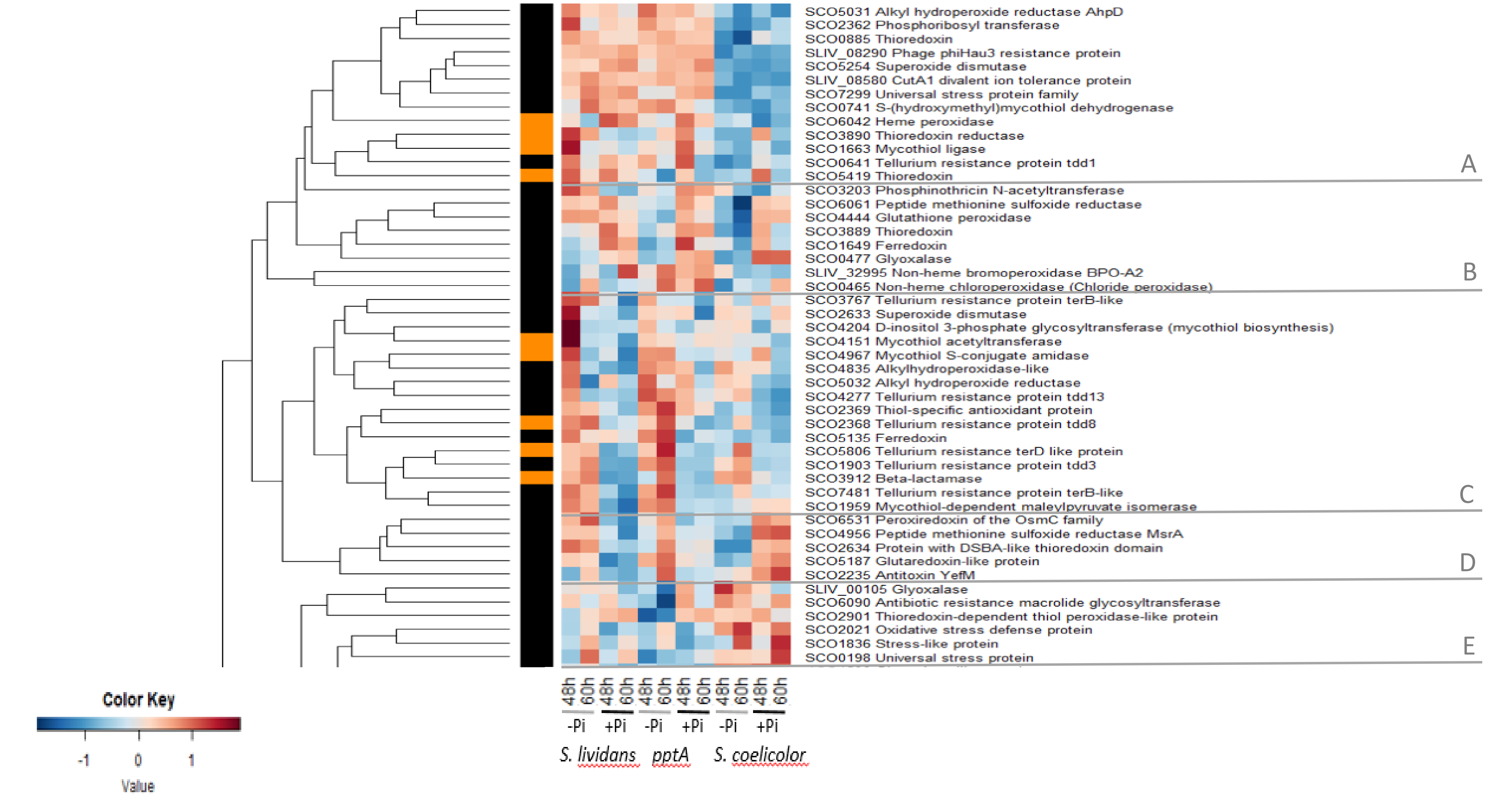

STRESS RESISTANCE
SCO5031 Alkyl hydroperoxide reductase AhpD
SCO2362 Phosphoribosyl transferase
SCO0885 Thioredoxin
SLIV_08290 Phage phiHau3 resistance protein
SCO5254 Superoxide dismutase
SLIV_08580 CutA1 divalent ion tolerance protein
SCO7299 Universal stress protein family
SCO0741 S-(hydroxymethyl)mycothiol dehydrogenase
SCO6042 Heme peroxidase
SCO3890 Thioredoxin reductase
SCO1663 Mycothiol ligase
SCO0641 Tellurium resistance protein tdd1
SCO5419 Thioredoxin
A
SCO3203 Phosphinothricin N-acetyltransferase
SCO6061 Peptide methionine sulfoxide reductase
SCO4444 Glutathione peroxidase
SCO3889 Thioredoxin
SCO1649 Ferredoxin
SCO0477 Glyoxalase
SLIV_32995 Non-heme bromoperoxidase BPO-A2
SCO0465 Non-heme chloroperoxidase (Chloride peroxidase)
B
SCO3767 Tellurium resistance protein terB-like
SCO2633 Superoxide dismutase
SCO4204 D-inositol 3-phosphate glycosyltransferase (mycothiol biosynthesis)
SCO4151 Mycothiol acetyltransferase
SCO4967 Mycothiol S-conjugate amidase
SCO4835 Alkylhydroperoxidase-like
SCO5032 Alkyl hydroperoxide reductase
SCO4277 Tellurium resistance protein tdd13
SCO2369 Thiol-specific antioxidant protein
SCO2368 Tellurium resistance protein tdd8
SCO5135 Ferredoxin
SCO5806 Tellurium resistance terD like protein
SCO1903 Tellurium resistance protein tdd3
SCO3912 Beta-lactamase
SCO7481 Tellurium resistance protein terB-like
SCO1959 Mycothiol-dependent maleylpyruvate isomerase
C
SCO6531 Peroxiredoxin of the OsmC family
SCO4956 Peptide methionine sulfoxide reductase MsrA
SCO2634 Protein with DSBA-like thioredoxin domain
SCO5187 Glutaredoxin-like protein
SCO2235 Antitoxin YefM
D
SLIV_00105 Glyoxalase
SCO6090 Antibiotic resistance macrolide glycosyltransferase
SCO2901 Thioredoxin-dependent thiol peroxidase-like protein
SCO2021 Oxidative stress defense protein
SCO1836 Stress-like protein
SCO0198 Universal stress protein
E
48h 60h 48h 60h 48h 60h 48h 60h 48h 60h 48h 60h
-Pi +Pi -Pi +Pi -Pi +Pi
S. lividans pptA S. coelicolor
Color Key
-1 0 1
Value

Figure S11B

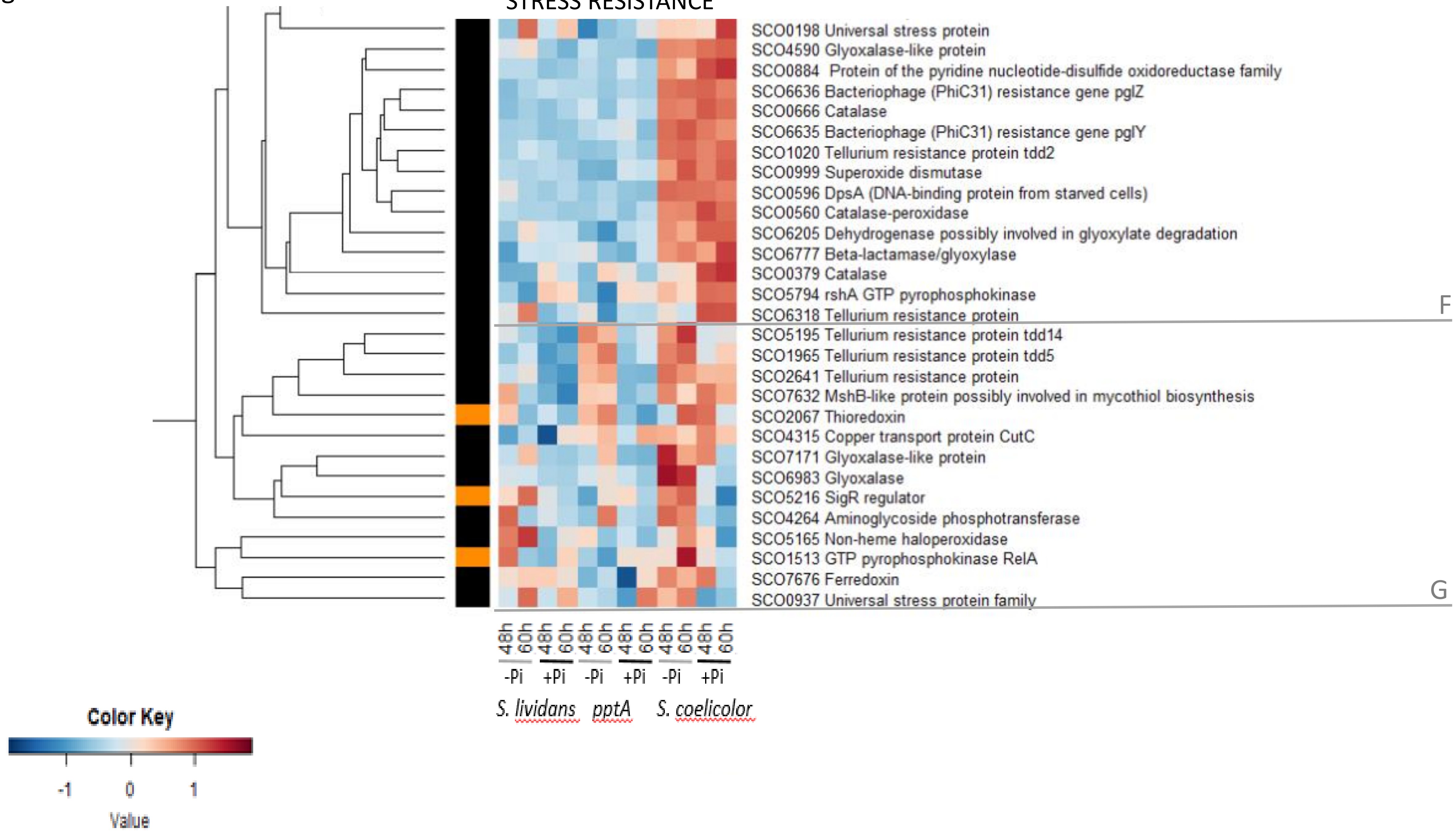
STRESS RESISTANCE
SCO0198 Universal stress protein
SCO4590 Glyoxalase-like protein
SCO0884 Protein of the pyridine nucleotide-disulfide oxidoreductase family
SCO6636 Bacteriophage (PhiC31) resistance gene pglZ
SCO0666 Catalase
SCO6635 Bacteriophage (PhiC31) resistance gene pglY
SCO1020 Tellurium resistance protein tdd2
SCO0999 Superoxide dismutase
SCO0596 DpsA (DNA-binding protein from starved cells)
SCO0560 Catalase-peroxidase
SCO6205 Dehydrogenase possibly involved in glyoxylate degradation
SCO6777 Beta-lactamase/glyoxylase
SCO0379 Catalase
SCO5794 rshA GTP pyrophosphokinase
SCO6318 Tellurium resistance protein
F
SCO5195 Tellurium resistance protein tdd14
SCO1965 Tellurium resistance protein tdd5
SCO2641 Tellurium resistance protein
SCO7632 MshB-like protein possibly involved in mycothiol biosynthesis
SCO2067 Thioredoxin
SCO4315 Copper transport protein CutC
SCO7171 Glyoxalase-like protein
SCO6983 Glyoxalase
SCO5216 SigR regulator
SCO4264 Aminoglycoside phosphotransferase
SCO5165 Non-heme haloperoxidase
SCO1513 GTP pyrophosphokinase RelA
SCO7676 Ferredoxin
SCO0937 Universal stress protein family
G
48h 60h 48h 60h 48h 60h 48h 60h 48h 60h 48h 60h
-Pi +Pi -Pi +Pi -Pi +Pi
S. lividans pptA S. coelicolor
Color Key
-1 0 1
Value

Figure S11C

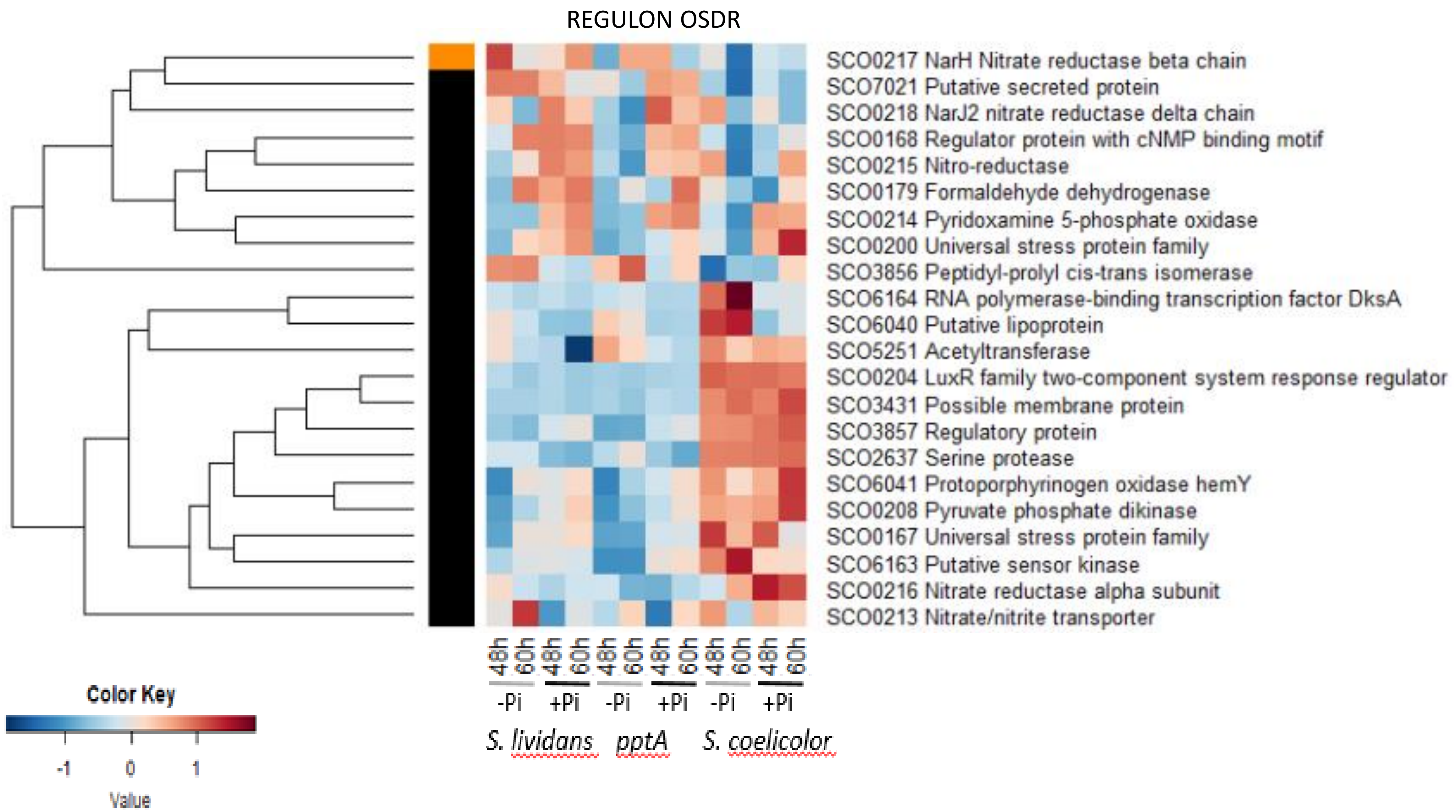
REGULON OSDR
SCO0217 NarH Nitrate reductase beta chain
SCO7021 Putative secreted protein
SCO0218 NarJ2 nitrate reductase delta chain
SCO0168 Regulator protein with cNMP binding motif
SCO0215 Nitro-reductase
SCO0179 Formaldehyde dehydrogenase
SCO0214 Pyridoxamine 5-phosphate oxidase
SCO0200 Universal stress protein family
SCO3856 Peptidyl-prolyl cis-trans isomerase
SCO6164 RNA polymerase-binding transcription factor DksA
SCO6040 Putative lipoprotein
SCO5251 Acetyltransferase
SCO0204 LuxR family two-component system response regulator
SCO3431 Possible membrane protein
SCO3857 Regulatory protein
SCO2637 Serine protease
SCO6041 Protoporphyrinogen oxidase hemY
SCO0208 Pyruvate phosphate dikinase
SCO0167 Universal stress protein family
SCO6163 Putative sensor kinase
SCO0216 Nitrate reductase alpha subunit
SCO0213 Nitrate/nitrite transporter
48h 60h 48h 60h 48h 60h 48h 60h 48h 60h 48h 60h
-Pi +Pi -Pi +Pi -Pi +Pi
S. lividans pptA S. coelicolor
Color Key
-1 0 1
Value

Figure S12

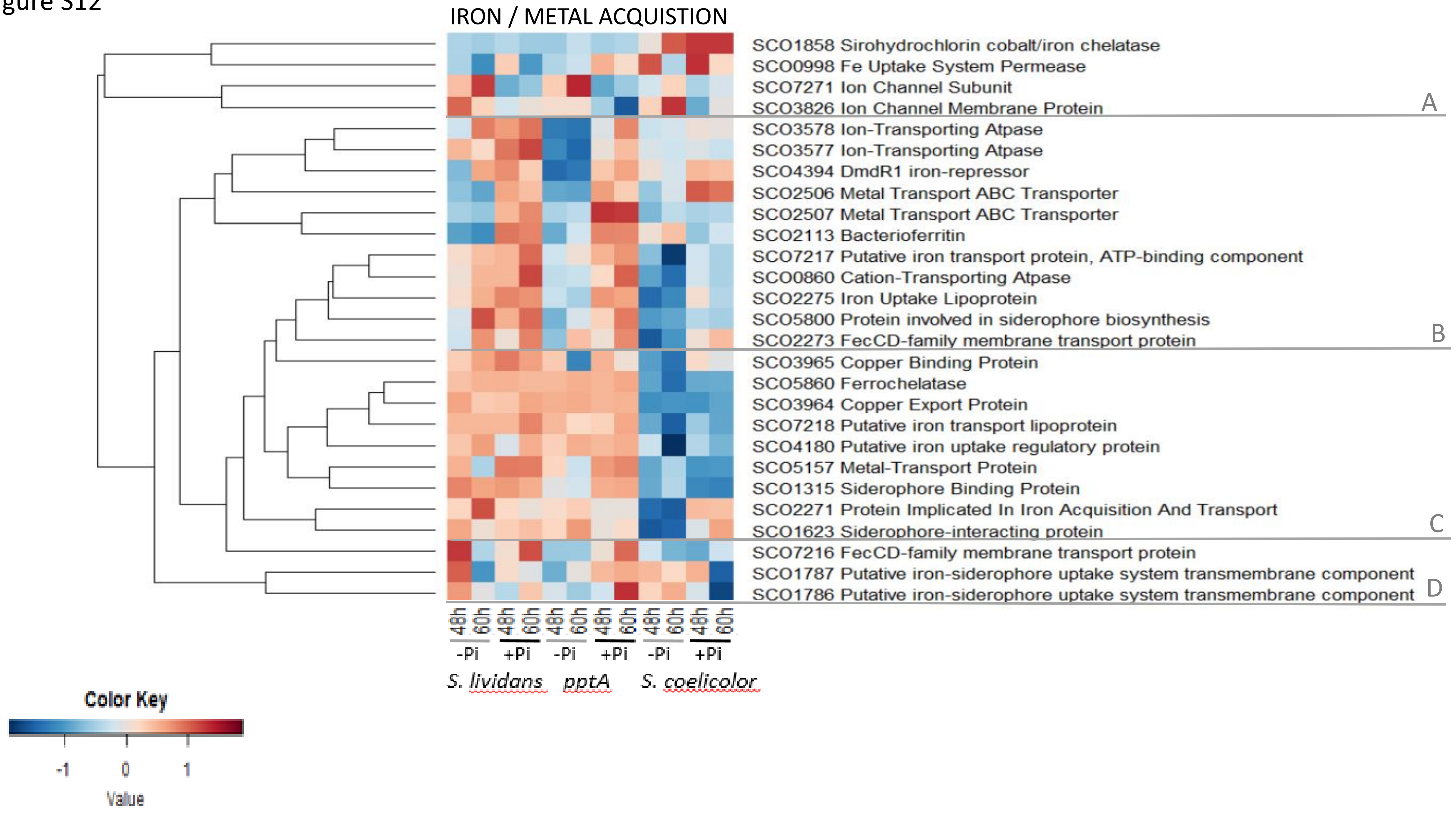
IRON / METAL ACQUISTION
SCO1858 Sirohydrochlorin cobalt/iron chelatase
SCO0998 Fe Uptake System Permease
SCO7271 Ion Channel Subunit
SCO3826 Ion Channel Membrane Protein
A
SCO3578 Ion-Transporting Atpase
SCO3577 Ion-Transporting Atpase
SCO4394 DmdR1 iron-repressor
SCO2506 Metal Transport ABC Transporter
SCO2507 Metal Transport ABC Transporter
SCO2113 Bacterioferritin
SCO7217 Putative iron transport protein, ATP-binding component
SCO0860 Cation-Transporting Atpase
SCO2275 Iron Uptake Lipoprotein
SCO5800 Protein involved in siderophore biosynthesis
SCO2273 FecCD-family membrane transport protein
B
SCO3965 Copper Binding Protein
SCO5860 Ferrochelatase
SCO3964 Copper Export Protein
SCO7218 Putative iron transport lipoprotein
SCO4180 Putative iron uptake regulatory protein
SCO5157 Metal-Transport Protein
SCO1315 Siderophore Binding Protein
SCO2271 Protein Implicated In Iron Acquisition And Transport
SCO1623 Siderophore-interacting protein
C
SCO7216 FecCD-family membrane transport protein
SCO1787 Putative iron-siderophore uptake system transmembrane component
SCO1786 Putative iron-siderophore uptake system transmembrane component
D
48h 60h 48h 60h 48h 60h 48h 60h 48h 60h 48h 60h
-Pi +Pi -Pi +Pi -Pi +Pi
S. lividans pptA S. coelicolor
Color Key
-1 0 1
Value

Figure S13

OTHER SPECIALIZED METABOLITES

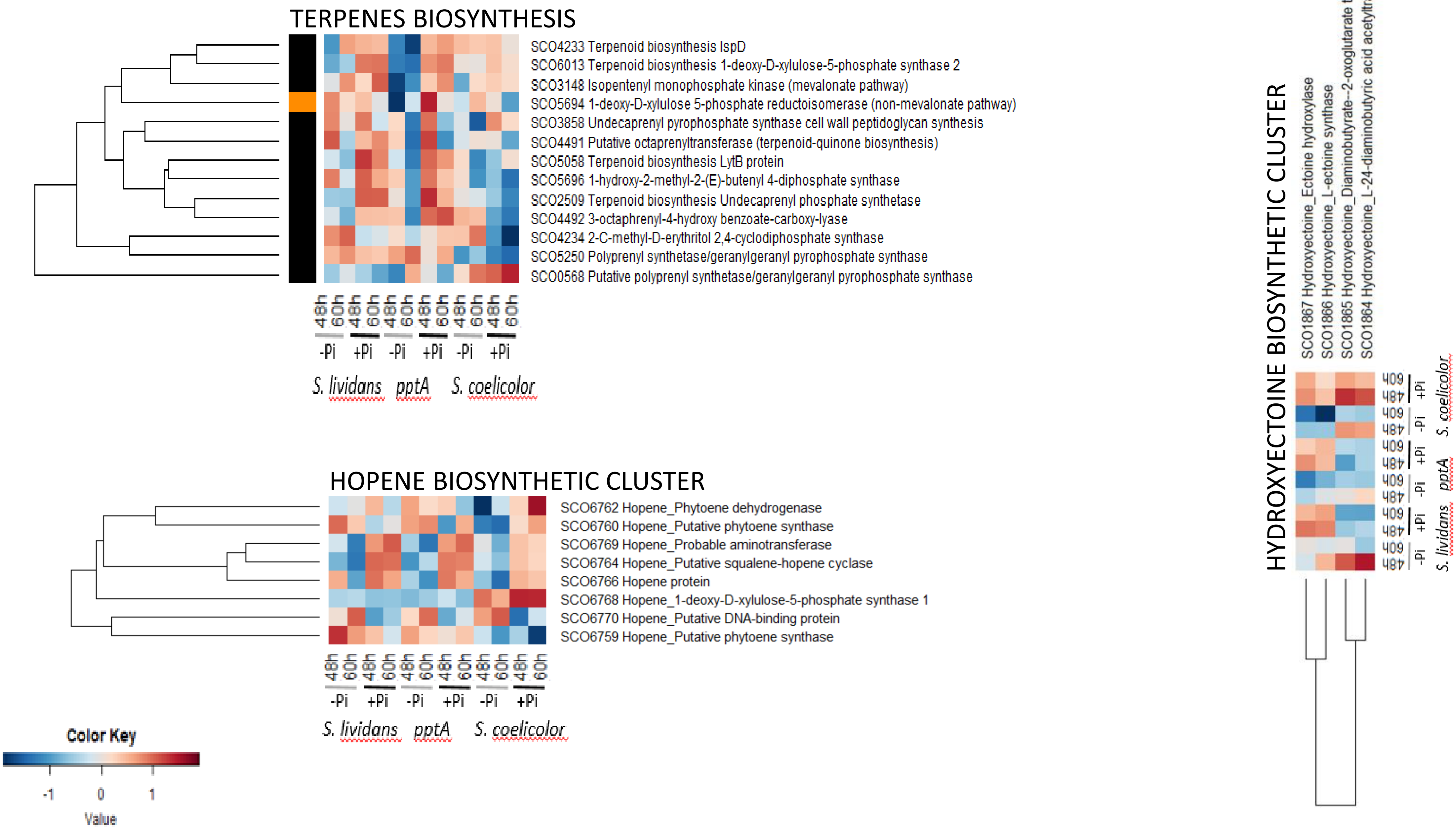

VITAMINS BIOSYNTHESIS

Figure S14A

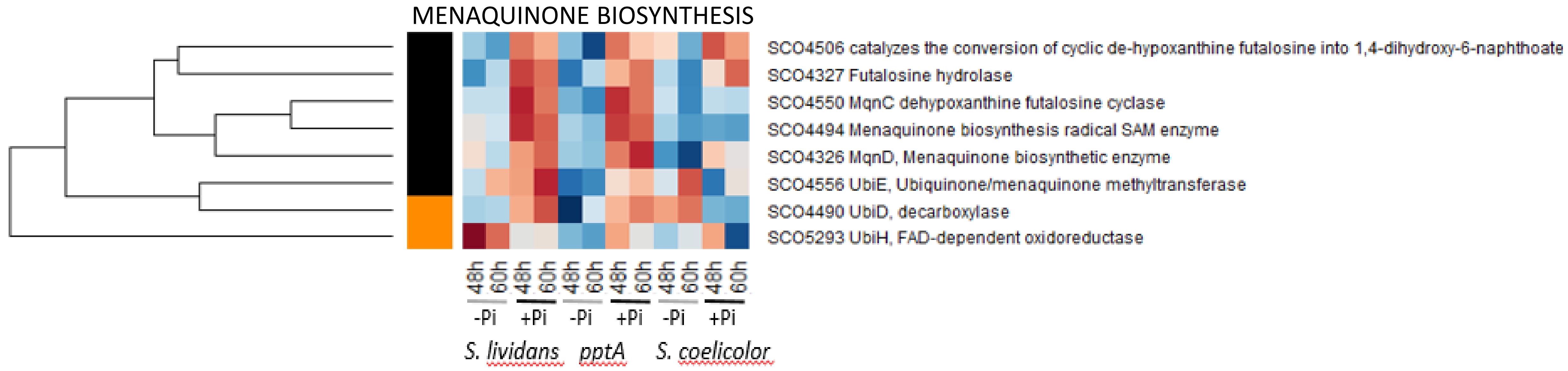
MENAQUINONE BIOSYNTHESIS
SCO4506 catalyzes the conversion of cyclic de-hypoxanthine futalosine into 1,4-dihydroxy-6-naphthoate
SCO4327 Futalosine hydrolase
SCO4550 MqnC dehypoxanthine futalosine cyclase
SCO4494 Menaquinone biosynthesis radical SAM enzyme
SCO4326 MqnD, Menaquinone biosynthetic enzyme
SCO4556 UbiE, Ubiquinone/menaquinone methyltransferase
SCO4490 UbiD, decarboxylase
SCO5293 UbiH, FAD-dependent oxidoreductase
48h 60h 48h 60h 48h 60h 48h 60h 48h 60h 48h 60h
-Pi +Pi -Pi +Pi -Pi +Pi
S. lividans pptA S. coelicolor

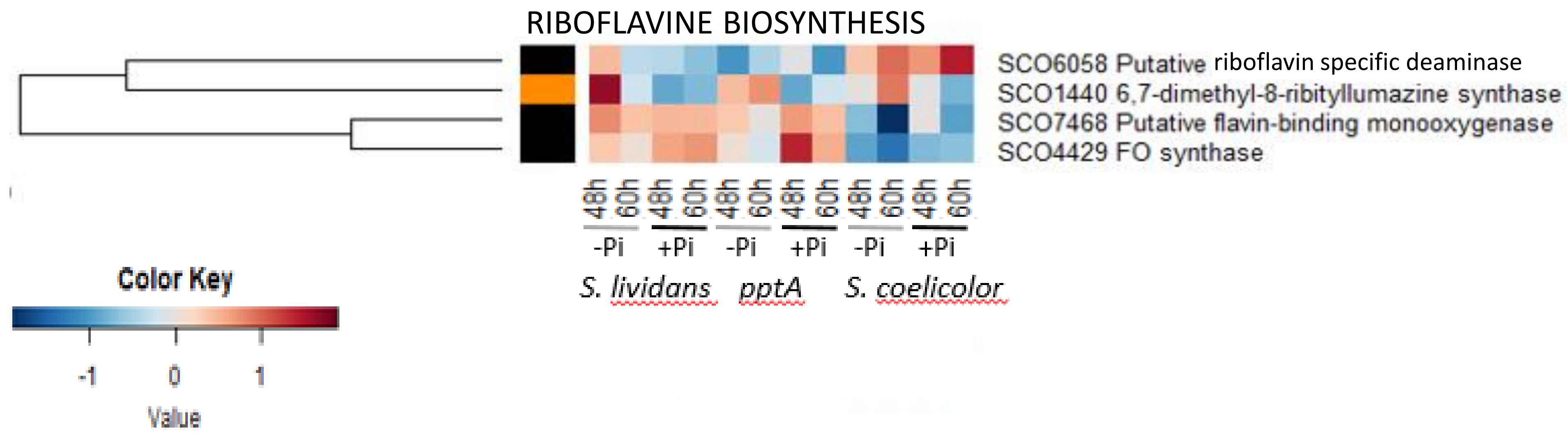
RIBOFLAVINE BIOSYNTHESIS
SCO6058 Putative riboflavin specific deaminase
SCO1440 6,7-dimethyl-8-ribityllumazine synthase
SCO7468 Putative flavin-binding monooxygenase
SCO4429 FO synthase
48h 60h 48h 60h 48h 60h 48h 60h 48h 60h 48h 60h
-Pi +Pi -Pi +Pi -Pi +Pi
S. lividans pptA S. coelicolor
Color Key
-1 0 1
Value

Figure S14B

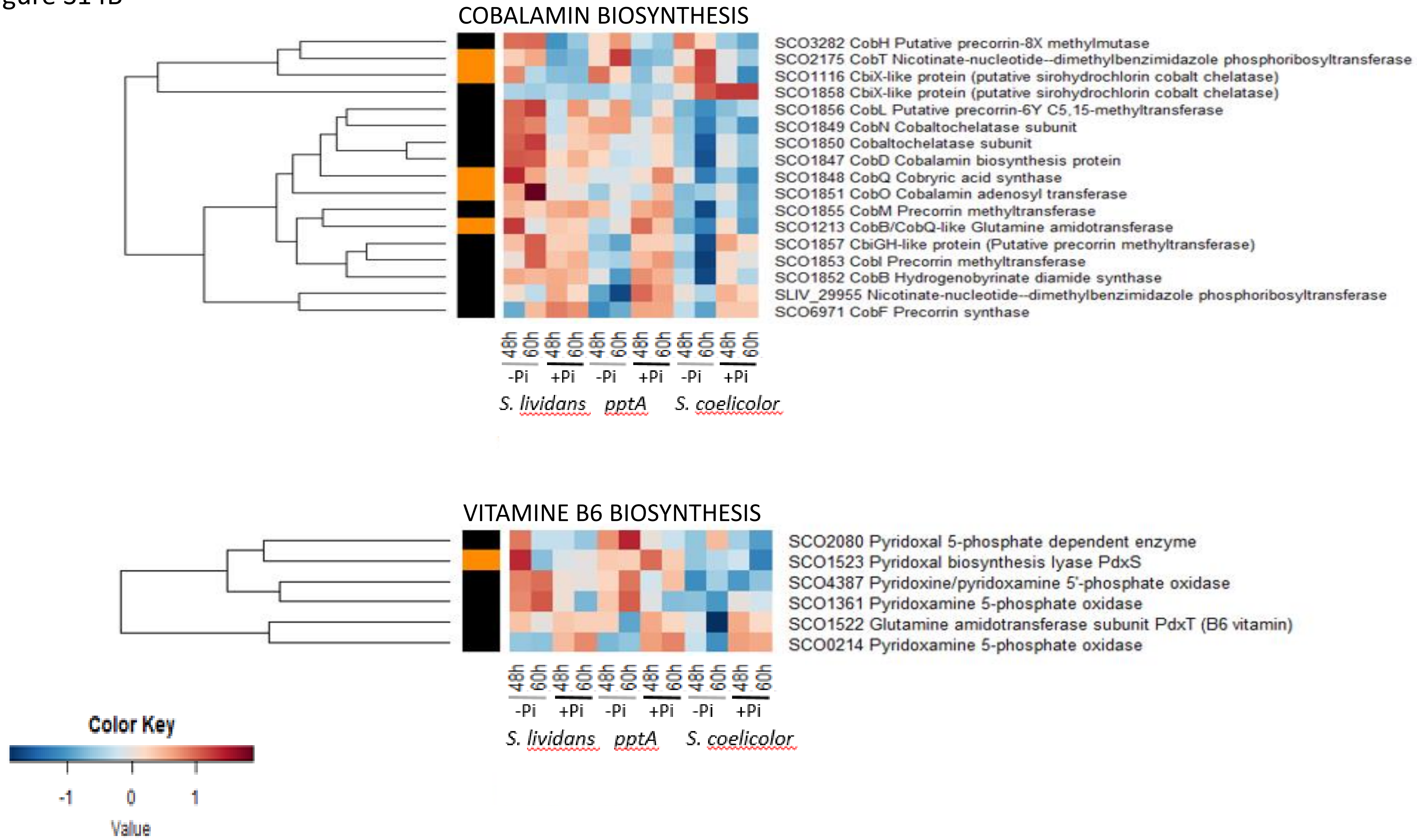
COBALAMIN BIOSYNTHESIS
SCO3282 CobH Putative precorrin-8X methylmutase
SCO2175 CobT Nicotinate-nucleotide--dimethylbenzimidazole phosphoribosyltransferase
SCO1116 CbiX-like protein (putative sirohydrochlorin cobalt chelatase)
SCO1858 CbiX-like protein (putative sirohydrochlorin cobalt chelatase)
SCO1856 CobL Putative precorrin-6Y C5,15-methyltransferase
SCO1849 CobN Cobaltochelatase subunit
SCO1850 Cobaltochelatase subunit
SCO1847 CobD Cobalamin biosynthesis protein
SCO1848 CobQ Cobryric acid synthase
SCO1851 CobO Cobalamin adenosyl transferase
SCO1855 CobM Precorrin methyltransferase
SCO1213 CobB/CobQ-like Glutamine amidotransferase
SCO1857 CbiGH-like protein (Putative precorrin methyltransferase)
SCO1853 CobI Precorrin methyltransferase
SCO1852 CobB Hydrogenobyrinate diamide synthase
SLIV_29955 Nicotinate-nucleotide--dimethylbenzimidazole phosphoribosyltransferase
SCO6971 CobF Precorrin synthase
48h 60h 48h 60h 48h 60h 48h 60h 48h 60h 48h 60h
-Pi +Pi -Pi +Pi -Pi +Pi
S. lividans pptA S. coelicolor
VITAMINE B6 BIOSYNTHESIS
SCO2080 Pyridoxal 5-phosphate dependent enzyme
SCO1523 Pyridoxal biosynthesis lyase PdxS
SCO4387 Pyridoxine/pyridoxamine 5'-phosphate oxidase
SCO1361 Pyridoxamine 5-phosphate oxidase
SCO1522 Glutamine amidotransferase subunit PdxT (B6 vitamin)
SCO0214 Pyridoxamine 5-phosphate oxidase
48h 60h 48h 60h 48h 60h 48h 60h 48h 60h 48h 60h
-Pi +Pi -Pi +Pi -Pi +Pi
S. lividans pptA S. coelicolor
Color Key
-1 0 1
Value

Figure S14C

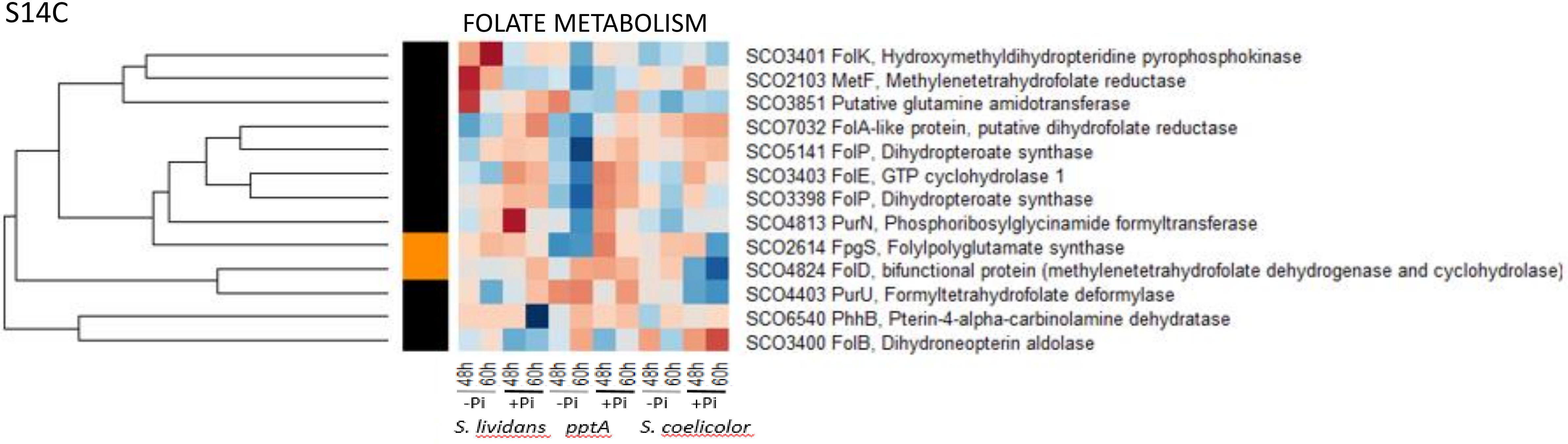
FOLATE METABOLISM
SCO3401 FolK, Hydroxymethyldihydropteridine pyrophosphokinase
SCO2103 MetF, Methylenetetrahydrofolate reductase
SCO3851 Putative glutamine amidotransferase
SCO7032 FolA-like protein, putative dihydrofolate reductase
SCO5141 FolP, Dihydropteroate synthase
SCO3403 FolE, GTP cyclohydrolase 1
SCO3398 FolP, Dihydropteroate synthase
SCO4813 PurN, Phosphoribosylglycinamide formyltransferase
SCO2614 FpgS, Folylpolyglutamate synthase
SCO4824 FolD, bifunctional protein (methylenetetrahydrofolate dehydrogenase and cyclohydrolase)
SCO4403 PurU, Formyltetrahydrofolate deformylase
SCO6540 PhhB, Pterin-4-alpha-carbinolamine dehydratase
SCO3400 FolB, Dihydroneopterin aldolase
48h 60h 48h 60h 48h 60h 48h 60h 48h 60h 48h 60h
-Pi +Pi -Pi +Pi -Pi +Pi
S. lividans pptA S. coelicolor

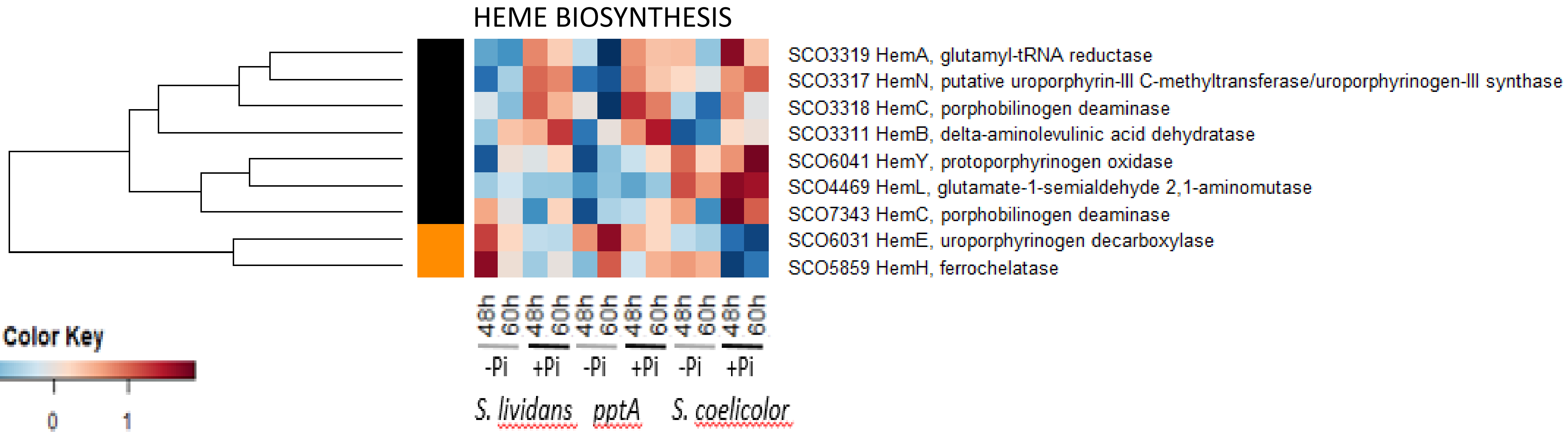
HEME BIOSYNTHESIS
SCO3319 HemA, glutamyl-tRNA reductase
SCO3317 HemN, putative uroporphyrin-III C-methyltransferase/uroporphyrinogen-III synthase
SCO3318 HemC, porphobilinogen deaminase
SCO3311 HemB, delta-aminolevulinic acid dehydratase
SCO6041 HemY, protoporphyrinogen oxidase
SCO4469 HemL, glutamate-1-semialdehyde 2,1-aminomutase
SCO7343 HemC, porphobilinogen deaminase
SCO6031 HemE, uroporphyrinogen decarboxylase
SCO5859 HemH, ferrochelatase
48h 60h 48h 60h 48h 60h 48h 60h 48h 60h 48h 60h
-Pi +Pi -Pi +Pi -Pi +Pi
S. lividans pptA S. coelicolor

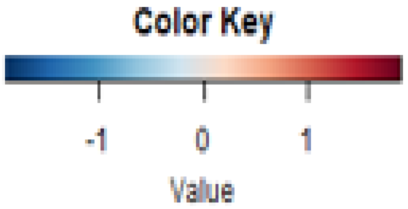
Color Key
-1 0 1
Value

Figure S14D

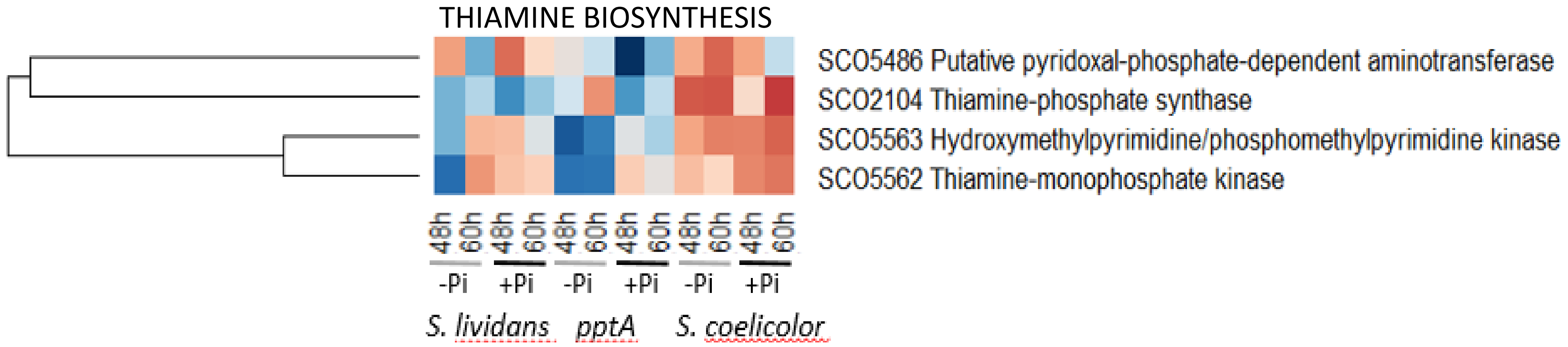
THIAMINE BIOSYNTHESIS
SCO5486 Putative pyridoxal-phosphate-dependent aminotransferase
SCO2104 Thiamine-phosphate synthase
SCO5563 Hydroxymethylpyrimidine/phosphomethylpyrimidine kinase
SCO5562 Thiamine-monophosphate kinase
48h 60h 48h 60h 48h 60h 48h 60h 48h 60h 48h 60h
-Pi +Pi -Pi +Pi -Pi +Pi
S. lividans pptA S. coelicolor

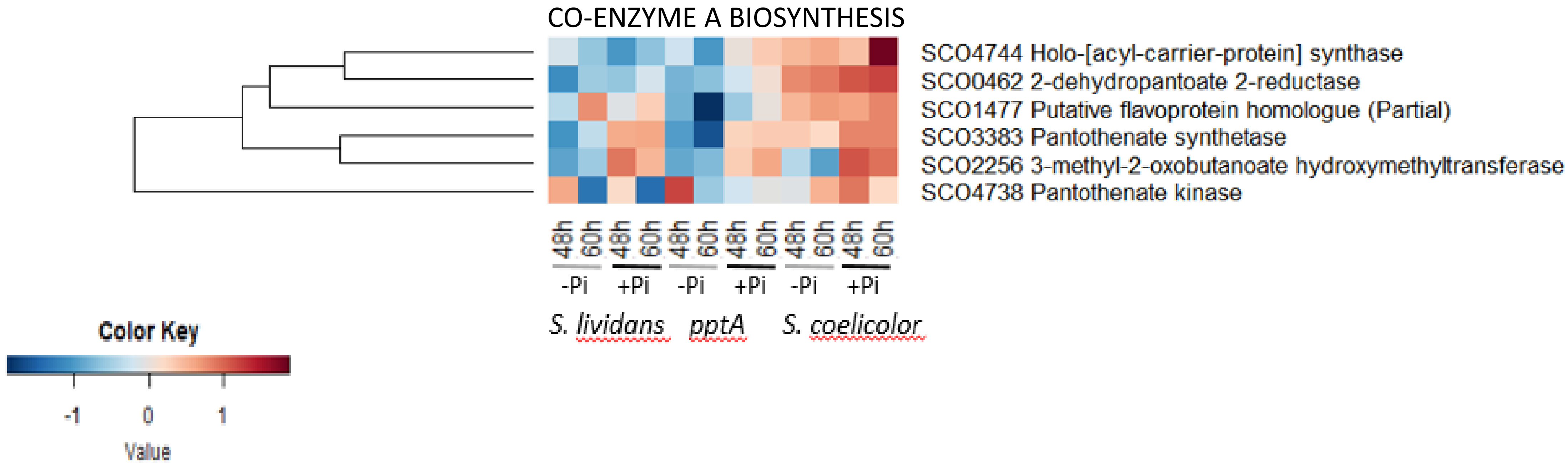
CO-ENZYME A BIOSYNTHESIS
SCO4744 Holo-[acyl-carrier-protein] synthase
SCO0462 2-dehydropantoate 2-reductase
SCO1477 Putative flavoprotein homologue (Partial)
SCO3383 Pantothenate synthetase
SCO2256 3-methyl-2-oxobutanoate hydroxymethyltransferase
SCO4738 Pantothenate kinase
48h 60h 48h 60h 48h 60h 48h 60h 48h 60h 48h 60h
-Pi +Pi -Pi +Pi -Pi +Pi
S. lividans pptA S. coelicolor
Color Key
-1 0 1
Value

Figure S14E

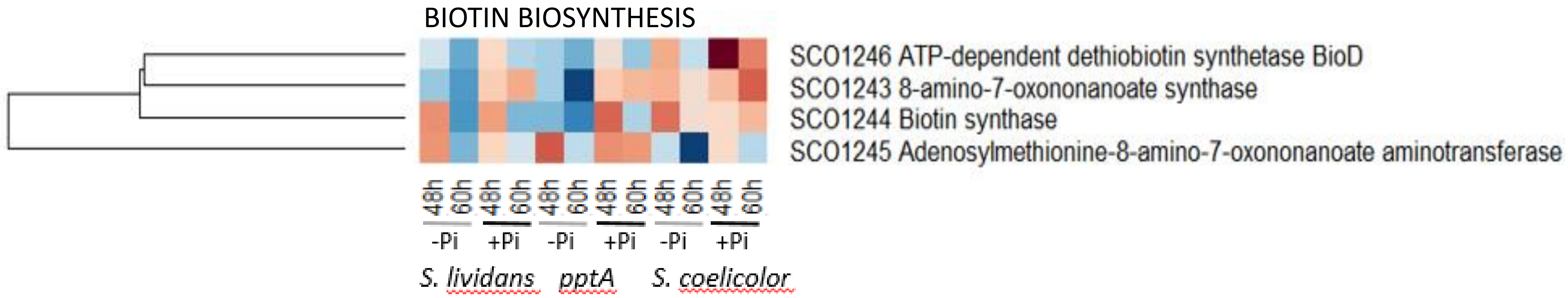
BIOTIN BIOSYNTHESIS
SCO1246 ATP-dependent dethiobiotin synthetase BioD
SCO1243 8-amino-7-oxononanoate synthase
SCO1244 Biotin synthase
SCO1245 Adenosylmethionine-8-amino-7-oxononanoate aminotransferase
48h 60h 48h 60h 48h 60h 48h 60h 48h 60h 48h 60h
-Pi +Pi -Pi +Pi -Pi +Pi
S. lividans pptA S. coelicolor

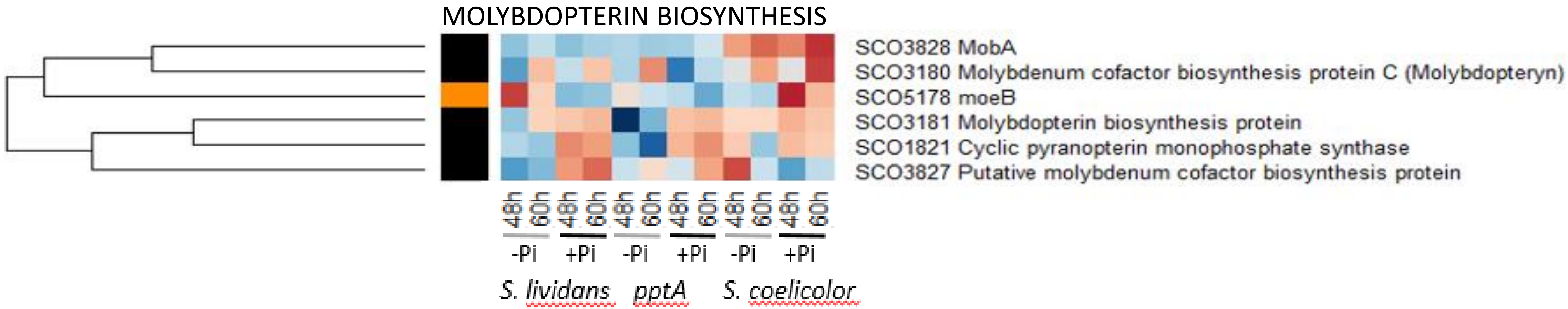
MOLYBDOPTERIN BIOSYNTHESIS
SCO3828 MobA
SCO3180 Molybdenum cofactor biosynthesis protein C (Molybdopteryn)
SCO5178 moeB
SCO3181 Molybdopterin biosynthesis protein
SCO1821 Cyclic pyranopterin monophosphate synthase
SCO3827 Putative molybdenum cofactor biosynthesis protein
48h 60h 48h 60h 48h 60h 48h 60h 48h 60h 48h 60h
-Pi +Pi -Pi +Pi -Pi +Pi
S. lividans pptA S. coelicolor

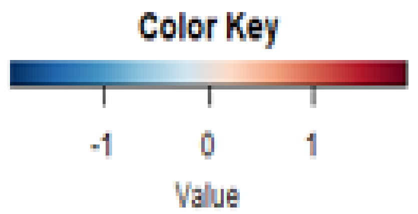
Color Key
-1 0 1
Value

TRANSPORT SYSTEMS

Figure S15A

TRANSPORT SYSTEMS INDUCED IN PHOSPHATE LIMITATION IN THE THREE STRAINS

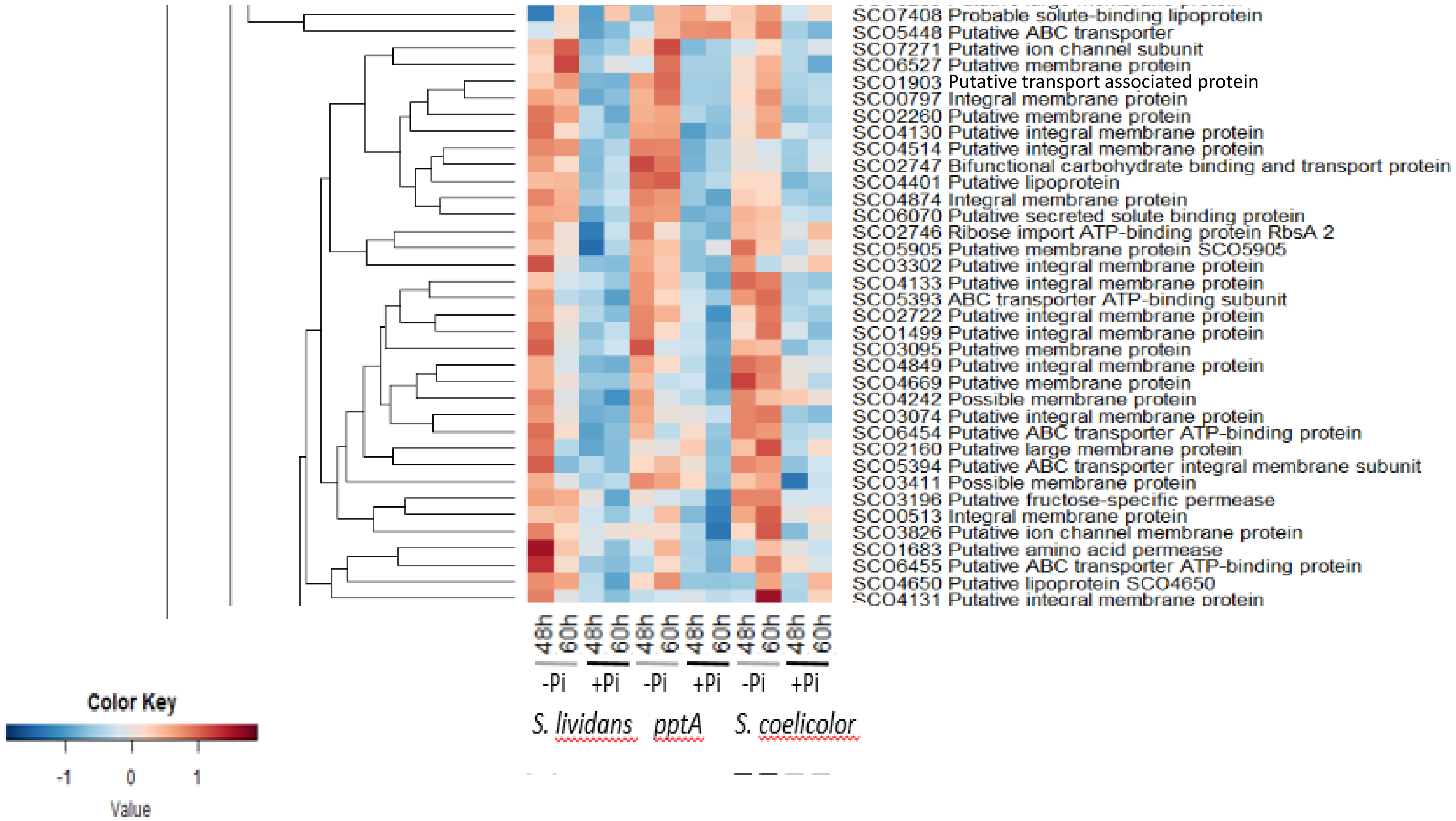

Figure S15B

TRANSPORT SYSTEMS INDUCED IN PHOSPHATE LIMITATION IN THE THREE STRAINS

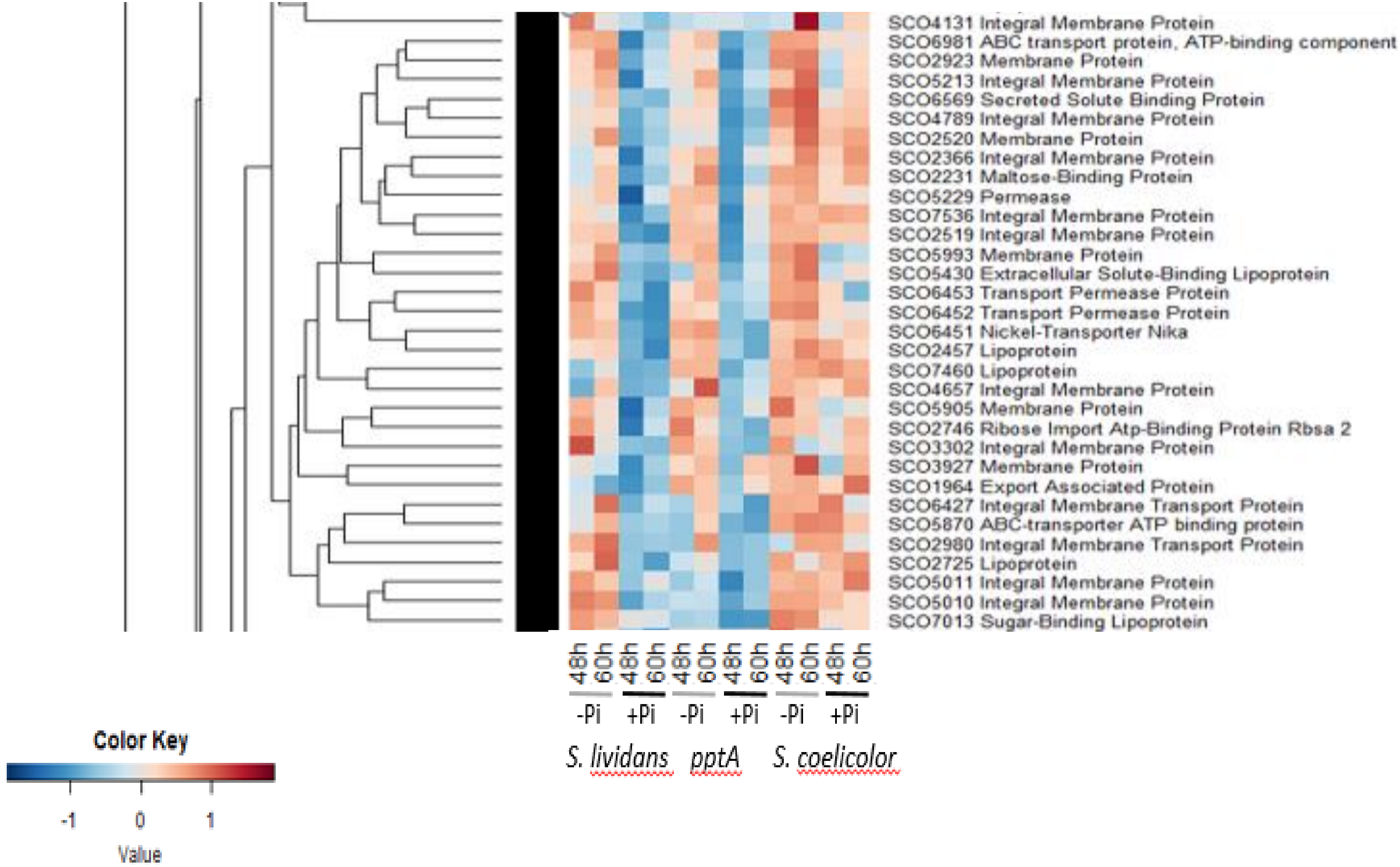

Figure S15C

TRANSPORT SYSTEMS INDUCED IN PHOSPHATE LIMITATION ONLY IN THE *S. LIVIDANS* STRAINS

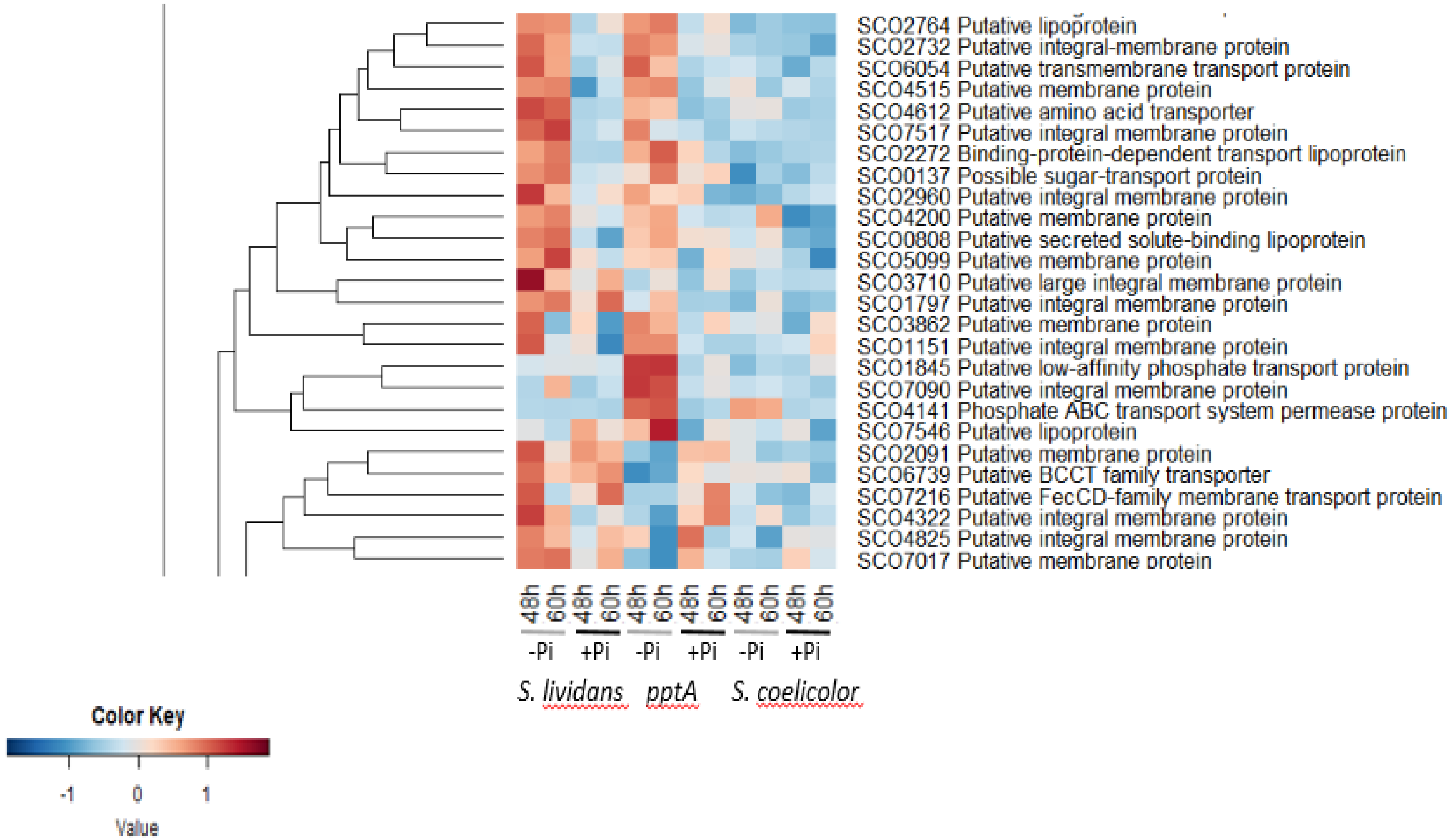

Figure S15D

TRANSPORT SYSTEMS INDUCED IN PHOSPHATE LIMITATION ONLY IN *S.COELICOLOR*

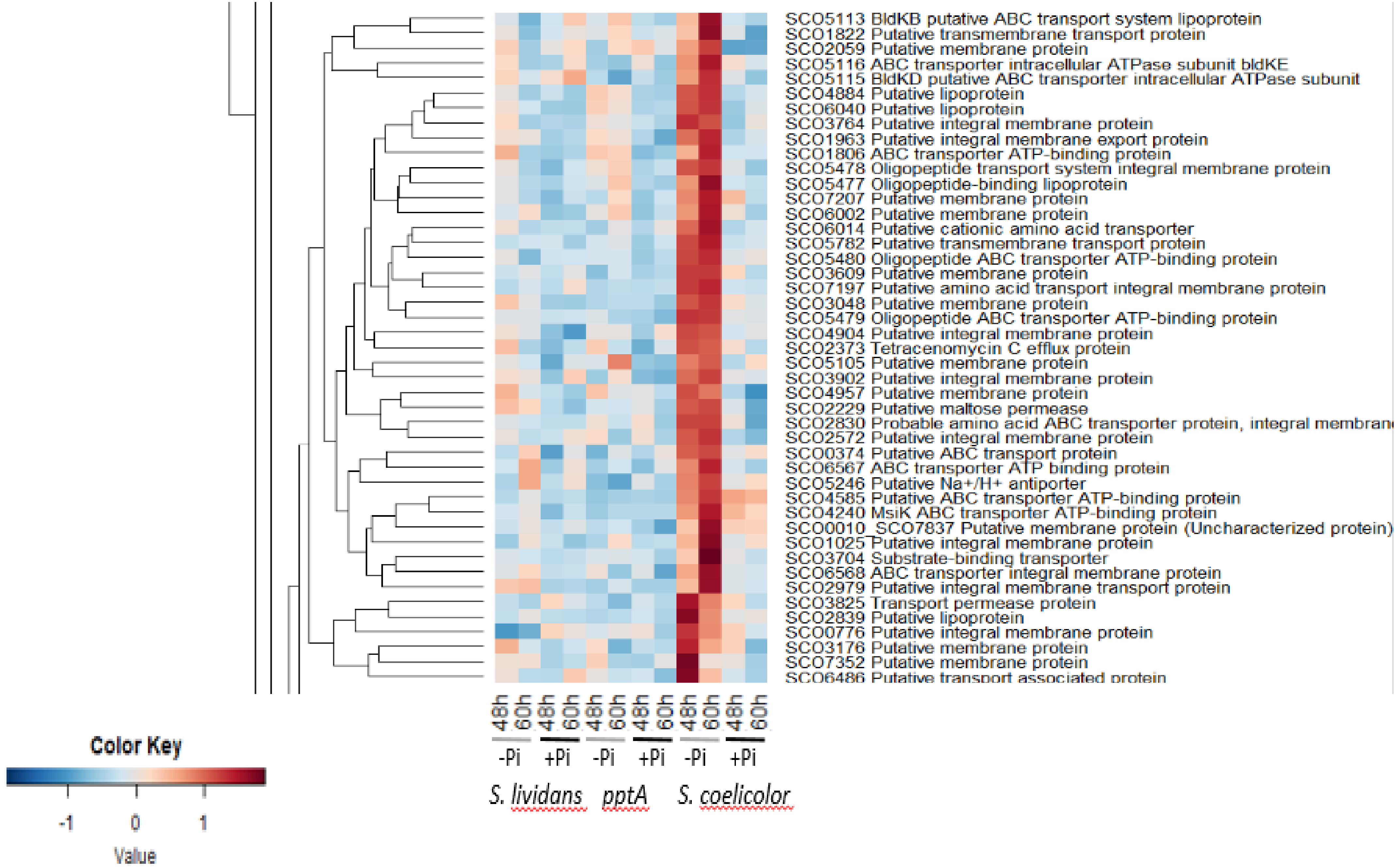

Figure S16A

TRANSPORT SYSTEMS INDUCED IN PHOSPHATE PROFICIENCY IN THE THREE STRAINS

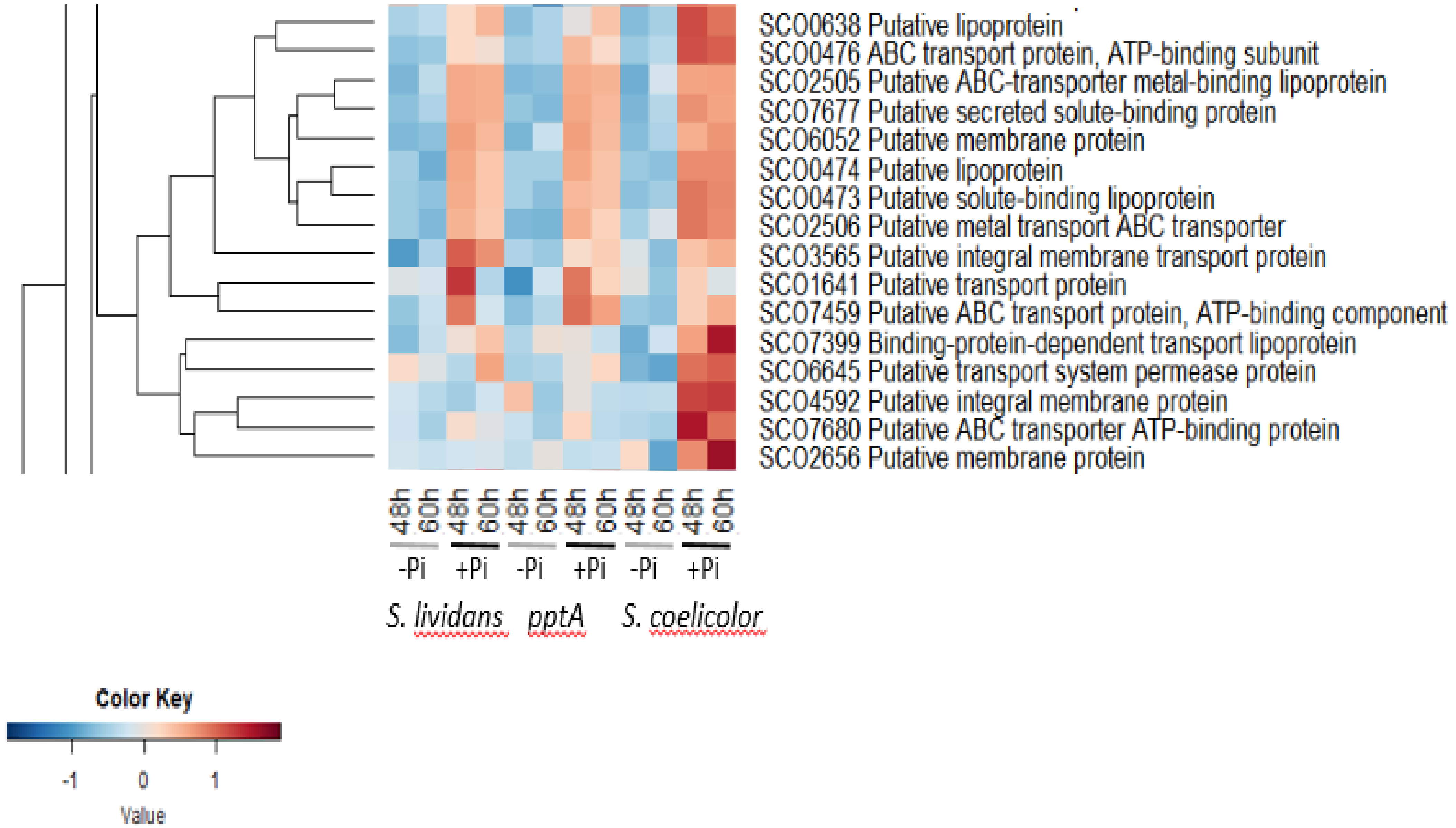

Figure S16B TRANSPORT SYSTEMS INDUCED IN PHOSPHATE PROFICIENCY MAINLY IN THE *S. LIVIDANS* STRAINS

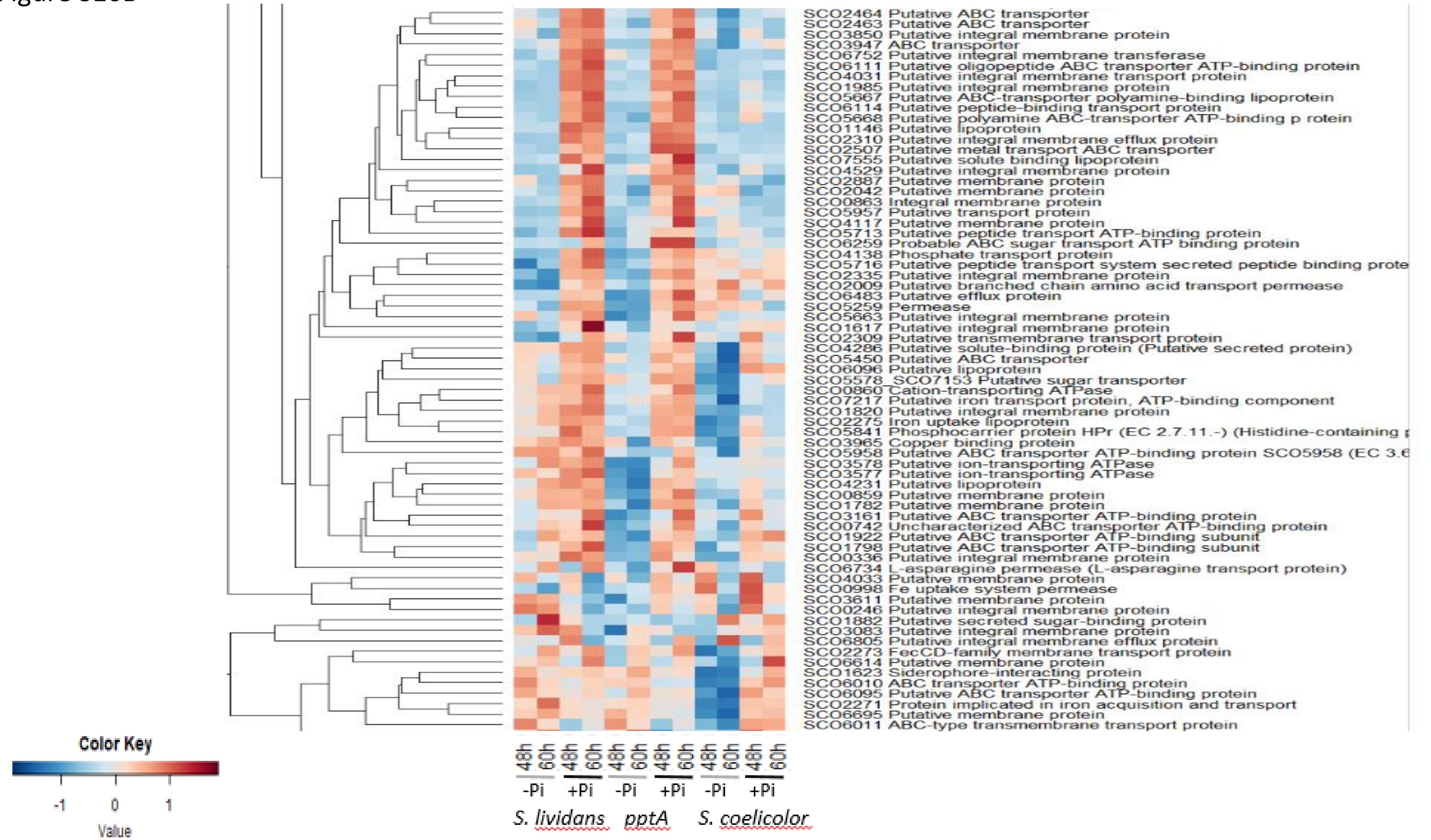

Figure S17A

TRANSPORTS SYSTEMS HIGHLY ABUNDANT IN BOTH PHOSPHATE CONDITIONS MAINLY THE *S. LIVIDANS* STRAINS

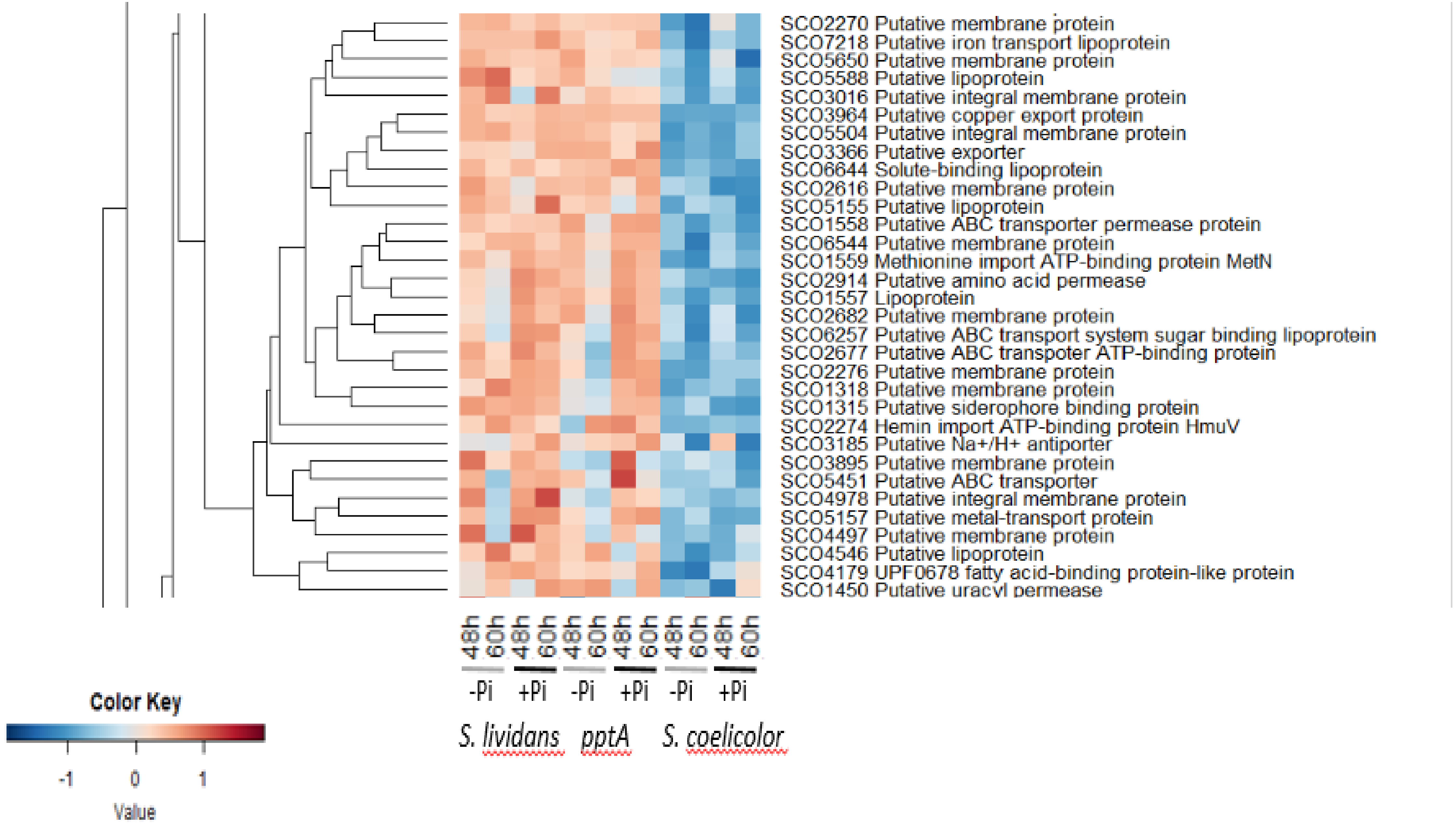

TRANSPORTS SYSTEMS HIGHLY ABUNDANT IN BOTH PHOSPHATE CONDITIONS, MAINLY IN *S. COELICOLOR*

Figure S17B

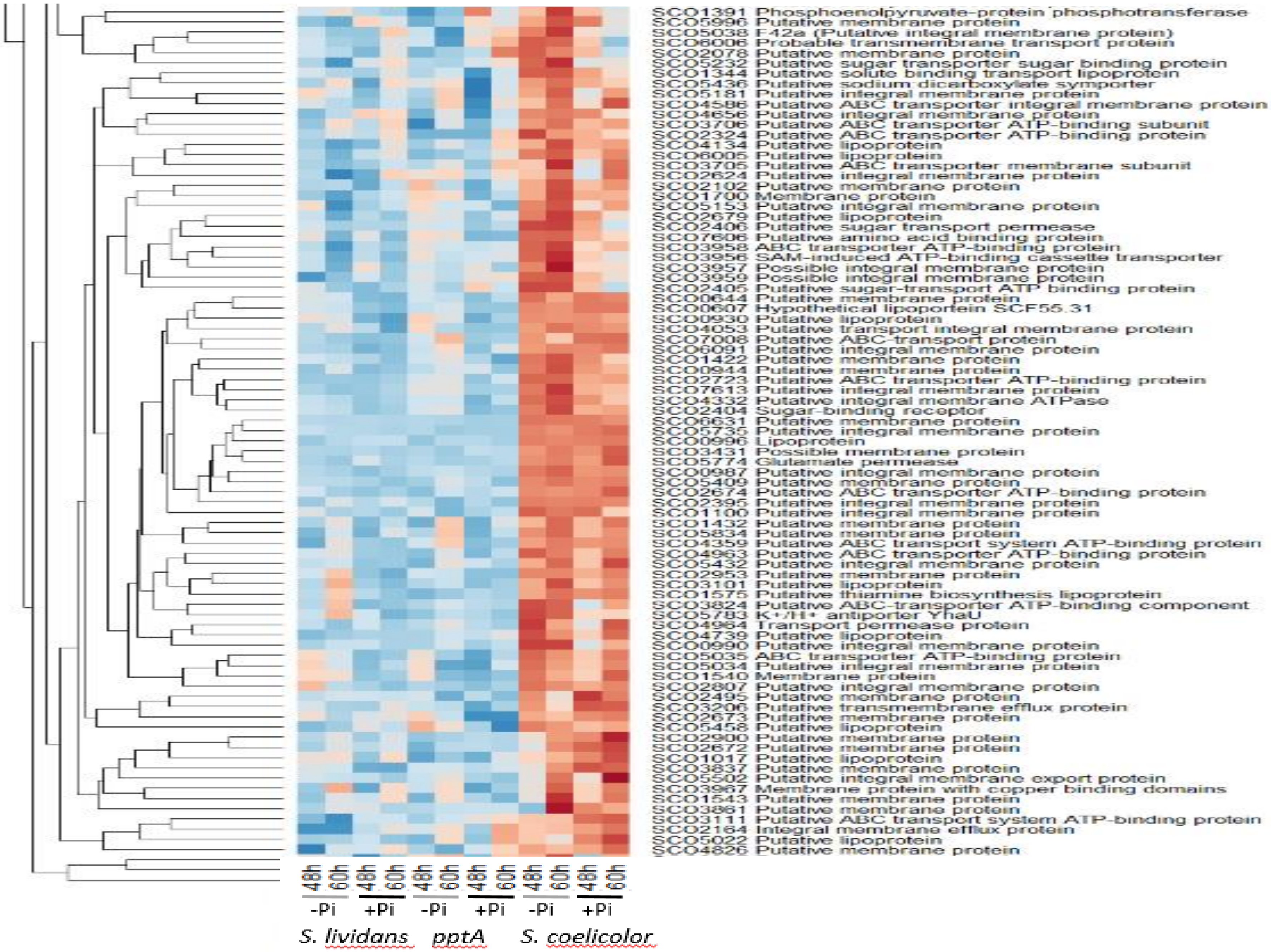

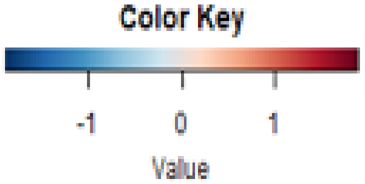

Figure S18A

TRANSPORT SYSTEMS POORLY ABUNDANT IN PHOSPHATE LIMITATION IN THE *PPTA* MUTANT OF *S. LIVIDANS.*

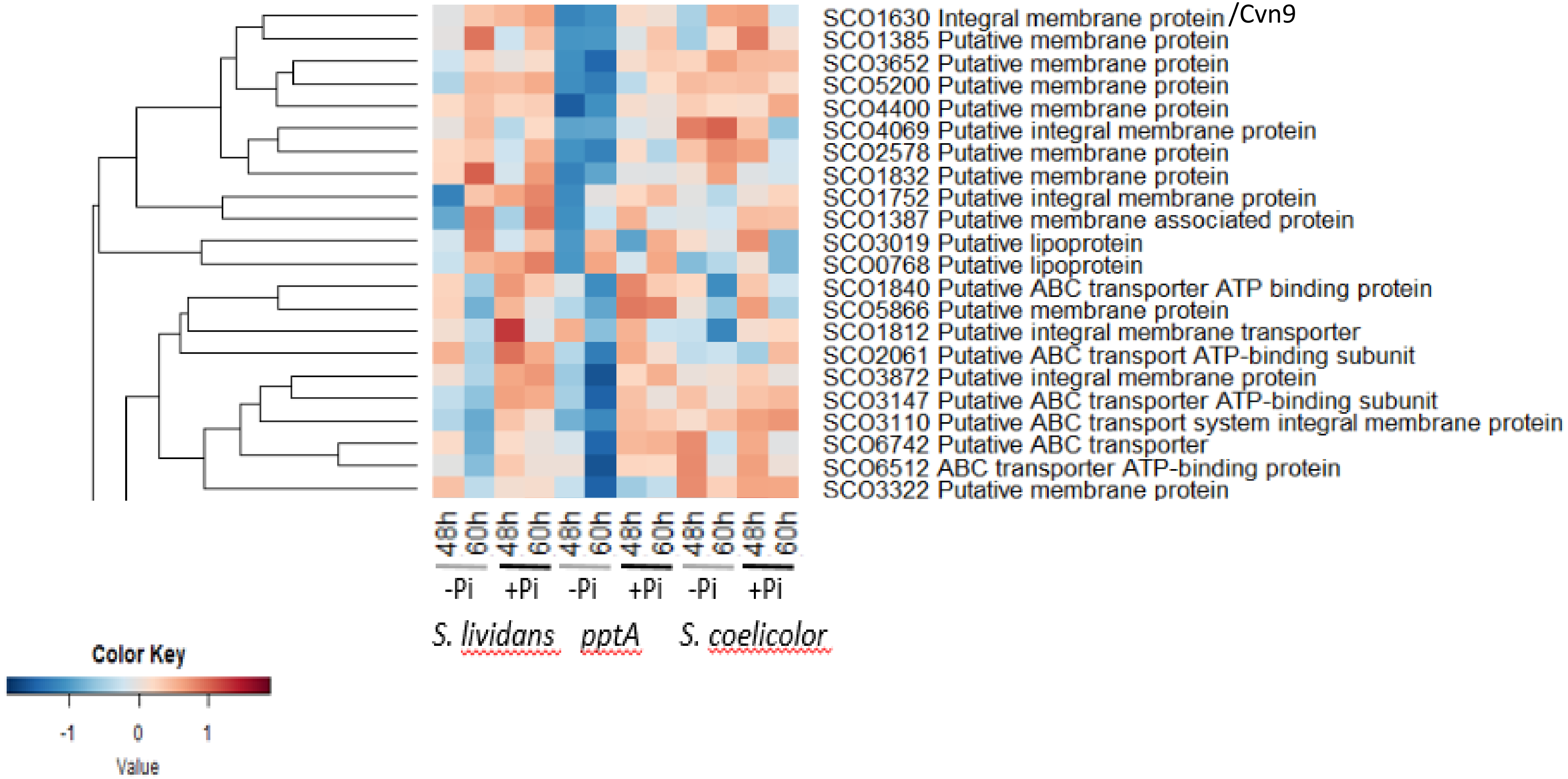

Figure S18B

TRANSPORT SYSTEMS POORLY ABUNDANT IN PHOSPHATE LIMITATION IN THE *PPTA* MUTANT OF *S. LIVIDANS*

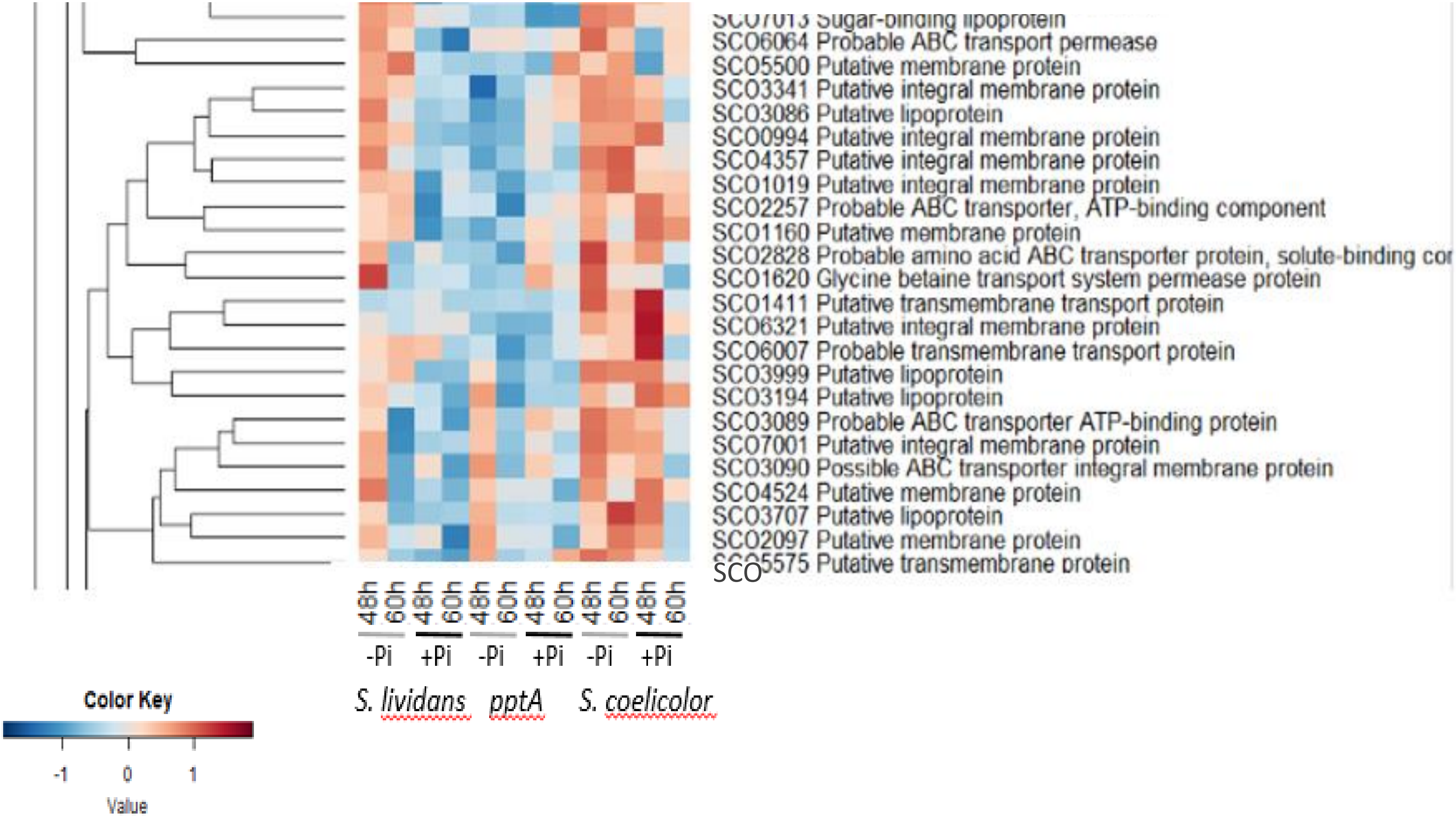

Figure S19

TRANSPORT SYSTEMS POORLY ABUNDANT IN PHOSPHATE PROFICIENCY IN *S.COELICOLOR*

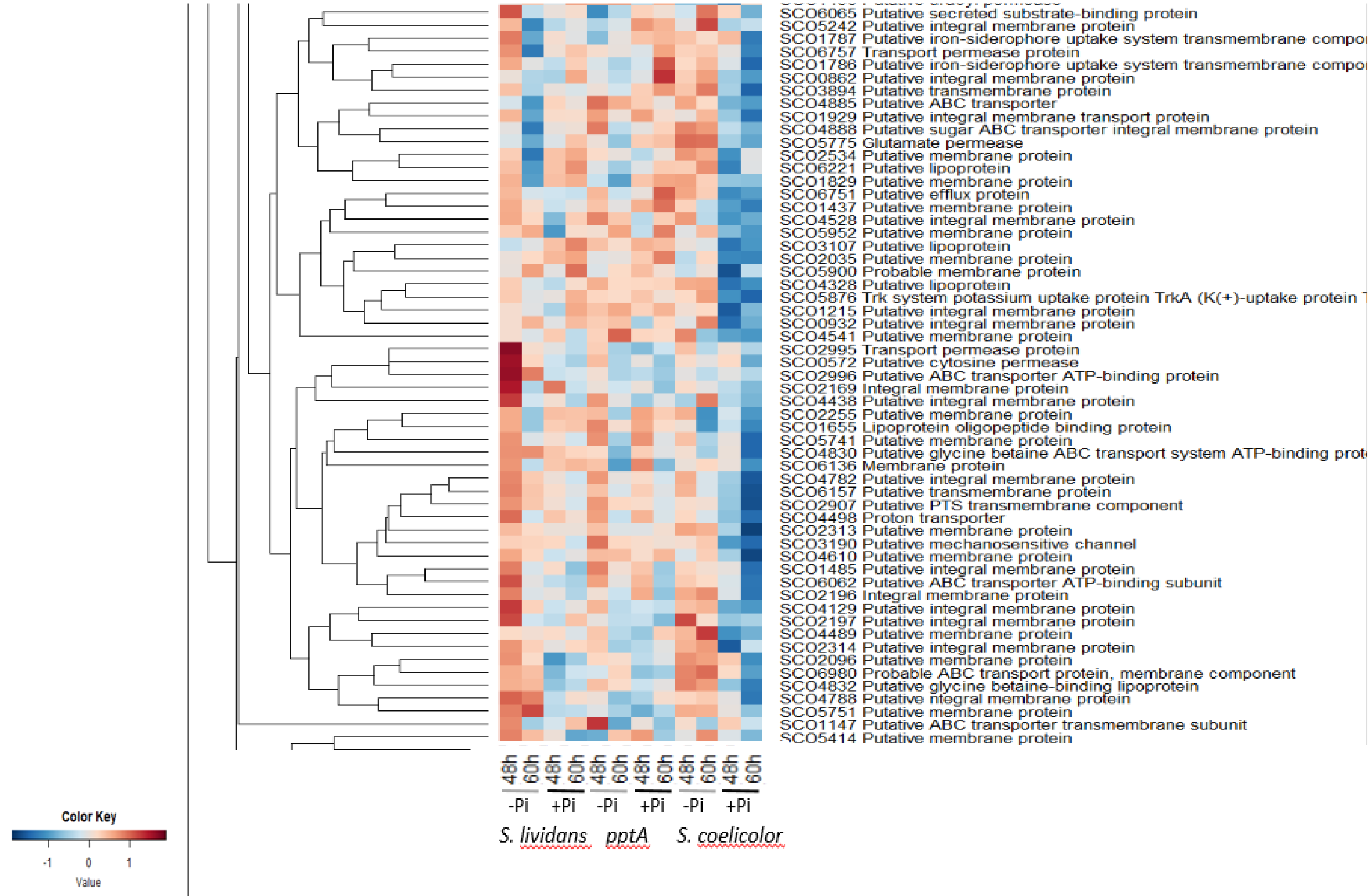

Supplement: Supplementary Figure 1 — Heatmap representation of proteins of pyruvate/acetylCoA and glycerol metabolism. [file Data_Sheet_1.pdf]
